# Mathematical Modeling Quantifies “Just-Right” APC Inactivation for Colorectal Cancer Initiation

**DOI:** 10.1158/0008-5472.CAN-25-0445

**Published:** 2025-12-15

**Authors:** Meritxell Brunet Guasch, Nathalie A. Feeley, Ignacio Soriano, Steve Thorn, Ian P.M. Tomlinson, Michael D. Nicholson, Tibor Antal

**Affiliations:** 1 School of Mathematics and the Maxwell Institute for Mathematical Sciences, University of Edinburgh, Edinburgh, United Kingdom; 2 CRUK Scotland Centre, Institute of Genetics and Cancer, University of Edinburgh, Edinburgh, United Kingdom; 3 Department of Oncology, University of Oxford, Oxford, United Kingdom

## Abstract

Dysregulation of the tumor suppressor gene *APC* is a canonical step in colorectal cancer development by promoting activation of the WNT/β-catenin pathway. Curiously, most colorectal tumors carry biallelic mutations that result in only partial loss of APC function, suggesting that a “just-right” level of APC inactivation, and hence WNT signaling, provides the optimal conditions for tumorigenesis. Mutational processes act variably across the *APC* gene, which could contribute to the bias against complete APC inactivation. Here, we proposed a mathematical model to quantify the tumorigenic effect of biallelic *APC* genotypes, controlling for somatic mutational processes. Analysis of sequence data from >2500 colorectal cancers showed that *APC* genotypes resulting in partial protein function confer about 50 times higher probability of progressing to cancer compared to complete APC inactivation. The optimal inactivation level varied with anatomical location and additional mutations of WNT pathway regulators. Assessment of the regulatory effects of secondary alterations in WNT drivers in combination with APC *in vivo* provided evidence that *AMER1* mutations increase WNT activity in tumors with suboptimal *APC* genotypes. The fitness landscape of APC inactivation was consistent across microsatellite unstable and *POLE*-deficient colorectal cancers and tumors in patients with familial adenomatous polyposis. Together, these findings suggest a general “just-right” optimum for APC inactivation and WNT signaling, pointing to WNT hyperactivation as a potential vulnerability in cancer.

## Abbreviations

*APC*
: Adenomatous Polyposis Coli
CRC: colorectal cancer
20AARs: 20 amino-acid-repeats
CID: β-Catenin Inhibitory Domain
FAP: familial adenomatous polyposis
NMD: nonsense mediated decay
MSS: microsatellite stable
WGD: whole genome duplication
CL-LOH: copy-loss loss of heterozygosity
CN-LOH: copy-neutral loss of heterozygosity
MSI: micro-satellite instability
SBS: single-base-substitutions
CMS: Consensus Molecular Subtypes
indels: small insertions or deletions
*POLE*
: DNA polymerase epsilon
100kGP: 100,000 Genomes Project

## Introduction

Colorectal cancer (CRC) is one of the most common and deadly cancers, with 1.9 million new cases diagnosed and 935,000 associated deaths in 2020 worldwide [1]. The Adenomatous Polyposis Coli (*APC*) gene is a canonical tumor suppressor, with loss-of-function mutations present in over 80% of sporadic CRCs [2,3]. *APC* mutations are one of the earliest, if not the earliest, genetic events in the development of CRC [4]. By dysregulating the WNT signalling pathway, biallelic inactivation of APC in healthy colonic cells leads to the formation of adenomatous polyps, which can progress to carcinoma [5,6].

**Figure 1:**
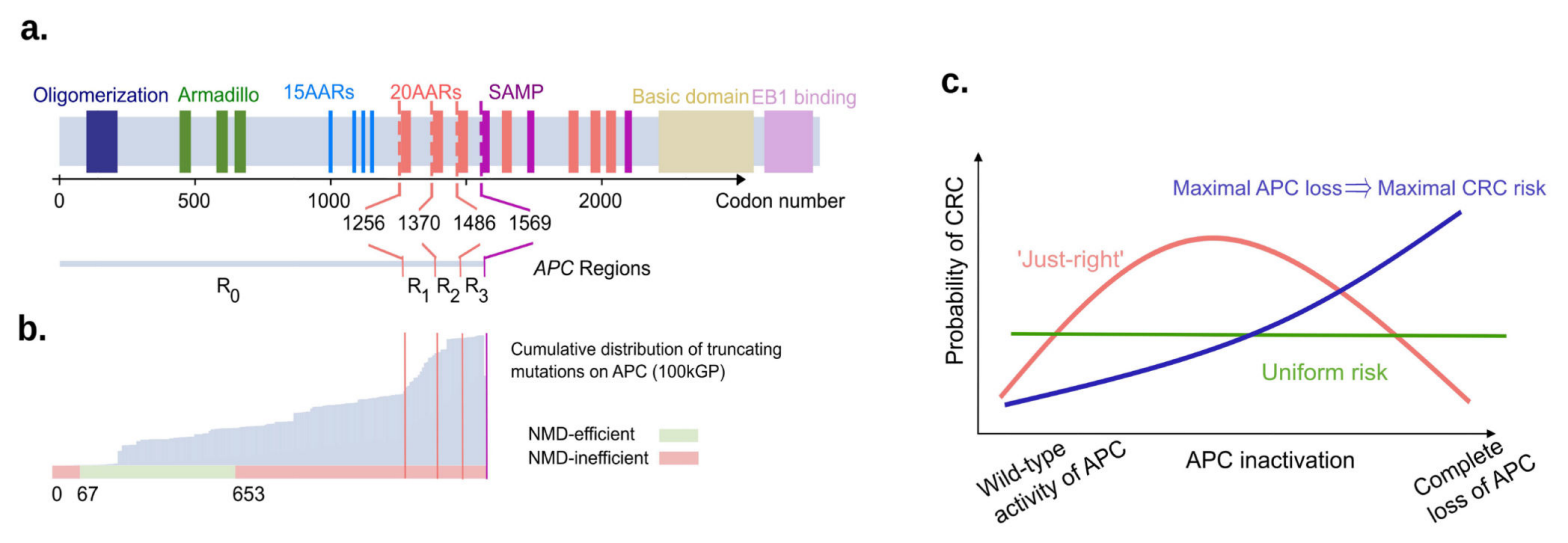
Evidence for ‘just-right’ in sporadic CRC. (a) Schematic showing the functional domains and regions of interest of *APC*, and their corresponding codon position. (b) In grey, the cumulative distribution of truncating mutations of *APC* in the 100kGP cohort of CRCs. Below, classification of codons by efficiency of NMD. Truncating mutations affecting codons in red are expected to evade Nonsense Mediated Decay as they occur either between the start codon and upstream of the 200th nucleotide or downstream of the last exon-exon junction [14]. Notably, most truncating mutations occur downstream codon 653, which are expected to evade NMD. (c) Schematic of the ‘just-right’ hypothesis which posits that an intermediate level of APC inactivation maximises CRC risk, in contrast with all genotypes conferring equal risk (‘Uniform risk’) and maximal APC loss conferring the maximal CRC risk.

Wild type APC acts as a scaffold protein for the β-catenin destruction complex, functioning as a tumor suppressor via regulation of the WNT pathway [7,8]. This activity involves several protein domains, including: short repeat sequences known as 20 amino-acid-repeats (20AARs), which bind to β-catenin; the β-Catenin Inhibitory Domain (CID); and the first SAMP domain, which acts as a binding site for AXIN (Figure 1a). *APC* is a classical tumor suppressor gene, requiring both alleles to be mutated for loss of function. Upon biallelic inactivation, APC loss leads to the stabilisation and accumulation of β-catenin in the cytoplasm, which, upon translocation to the nucleus, upregulates the WNT pathway and feeds the affected cells with a permanent mitogenic signal [6].

Most sporadic CRCs carry mutations occurring upstream of the first SAMP repeat (codon 1569), but retain some of the 20AARs [9, 10]. A similar pattern has been observed in tumors of patients with familial adenomatous polyposis (FAP), where germline mutations removing all 20AARs are typically followed by somatic second-hit mutations that retain at least one 20AAR [11–13]. The 20AAR domains are within the large final translated exon of *APC* (codons 653-2843), in which stop-gain or frameshift mutations evade nonsense mediated decay (NMD) [14], resulting in the synthesis of truncated proteins with attenuated β-catenin binding activity [15] (Figure 1b). Progressive retention of APC regulatory repeat sequences has been associated with a successive decrease in WNT signalling in various experimental model systems [16,17]. In particular, mutations upstream of the first 20AAR (codons 0-1256) result in maximal constitutive WNT activity [18,19], but are rarely observed in colorectal tumors. Though the naive expectation is that complete loss of a tumor suppressor gene’s function should be optimal for tumorigenesis, in most lesions APC is not fully inactivated.

These observations have led to the ‘just-right’ signalling hypothesis (Figure 1c), which states that both *APC* alleles are selected to retain sufficient β-catenin regulatory activity to generate an optimal WNT signalling level for tumor growth [12,13]. While the genetic data is consistent with the ‘just-right’ hypothesis [9,10,13], context-specific mutational processes - which could not be evaluated in the original ‘just-right’ studies - shape the mutation distribution observed in tumors and can cause mutation hotspots. For example, genomic regions with mononucleotide repeats are particularly susceptible to insertions and deletions [20], thus the 7-base thymine repeat starting at codon 1554 in *APC* might explain the enrichment for truncating mutations downstream of the 20AAR domains. Hence, the selective consequences of different *APC* genotypes *in vivo* cannot be directly concluded from their prevalence in sequence data. To resolve this, we propose a mathematical approach that allows us to quantify the probability of CRC progression of colonic stem cells with different *APC* genotypes, controlling for the underlying mutational processes in the colon. We apply our method to analyse the cohort of CRCs in the UK 100,000 Genomes Project [3], hereafter 100kGP, which includes allele-specific copy number alterations, providing an unprecedented opportunity to study the role of APC inactivation (n=1,366). Our findings are corroborated by an independent dataset (cBioPortal cohort n=1,305). We quantitatively test ‘just-right’ against the null ‘Uniform risk’ model, in which all *APC* genotypes provide the same selective advantage, and a model in which ‘maximal APC loss implies maximal risk’ (Figure 1b). Furthermore, we investigate tumor heterogeneity in relation to WNT activity based on the anatomical site of the lesion and the presence of additional mutations of secondary WNT regulators. Finally, the generality of the ‘just-right’ effect is examined by comparison with hypermutant CRCs and tumors from FAP patients.

## Materials and methods

### 1 Classification of APC-driven colorectal tumors

#### 1.1 Genomics England 100,000 Genomes Project

We analysed version 5 of the UK 100,000 Genomes Project, which performed whole genome sequencing on 2,023 paired cancer (~100x average depth) and normal (blood, 33x) samples from 2,017 CRC patients (median age 69, range 23–94; 59.4% male), as previously described by Cornish *et al*. [3]. *We included primary tumors with somatic pathogenic truncating APC* mutations upstream of the SAMP repeat (amino acid position 1569) that had not received radiotherapy prior to surgery, and did not harbor germline pathogenic mutations in *APC* or Lynch syndrome genes. Unless otherwise stated, we considered microsatellite stable (MSS) tumors with no pathogenic DNA polymerase epsilon (*POLE*) mutations (n=1,370, Supplementary Table 1).

#### 1.2 APC genotype classification

Truncating mutations (stop-gain and frameshift) were classified by regions relative to the 20 amino acid repeats (20AARs) as follows:

- Region *R_0_*: codons [0, 1256] (upstream the first 20AAR).
- Region *R_1_*: codons [1257, 1370] (downstream the first 20AAR and upstream the second 20AAR).
- Region *R_2_*: codons [1371, 1486] (downstream the second 20AAR and upstream the third 20AAR).
- Region *R_3_*: codons [1487, 1569] (downstream the third 20AAR and upstream the SAMP repeat).

Truncating mutations in regions *R*_1_, *R_2_* and *R_3_* were assumed to evade nonsense-mediated decay (NMD) [14], resulting in retention of 1, 2 and 3 20AARs, respectively; mutations in *R*_0_ were assumed to result in no 20AARs.

*APC* ploidy at tumor initiation was inferred using allele-specific copy number and whole genome duplication (WGD) status from Cornish *et al*. [3], *assuming that WGD occurred after APC inactivation. Given copy number at APC* site [a,b] (‘a’ for major allele, and ‘b’ from the minor allele), samples with no WGD were classified at initiation as: diploid if [1,1], copy-loss loss of heterozygosity (CL-LOH) if [1,0], and copy-neutral loss of heterozygosity (CN-LOH) if [a≥2,0]. Samples with WGD were classified as diploid at initiation if [a>0,b>0], CL-LOH if [1–2,0], or CN-LOH if [a>2,0].

For each sample, given *APC* truncating mutations and ploidy, we assign a biallelic genotype (*M,N*) where where *M*∈{0,1,2,3} denotes the region of the furthest upstream truncating mutation, and *N*∈{0,1,2,3,-,x2} is either the region of a second truncating mutation, a CL-LOH (denoted by -), or a CN-LOH (denoted by x2) (Table 1, Supplementary Table 2). If more than one clonal truncating mutation per allele was detected, we considered only the most upstream. We excluded samples that could not be classified to any of the above (Supplementary Table 1). For each genotype, the total number of retained 20AARs at initiation was inferred as in Table M2.

#### 1.3 Tumour site and additional driver mutations

Tumours were classified as proximal (n = 359) or distal (including rectum) (n = 620); others were excluded from site-specific analyses. Clonal driver mutations in WNT pathway genes (*AMER1, AXIN1, AXIN2, BCL9, BCL9L, CTNNB1, FBXW7, JUN, RNF43, SOX9, TCF7L2, ZNRF3, RSPO*) and major CRC drivers (*KRAS, TP53*) were determined as in Cornish *et al*. [3]

#### 1.4 MSI and POLE samples

Micro-satellite instability (MSI) and pathogenic *POLE* mutations were classified as in Cornish *et al*. [3]. We further evaluated *POLE* samples as hypermutant if they harboured >100 mutations/megabase. We considered samples with biallelic truncating *APC* mutations (n = 95 from a total of 360 MSI samples, and n=18 from 18 *POLE* samples).

#### 1.5 Independent CRC Cohorts

An independent cohort of *APC*-mutant CRCs from cBioPortal was processed analogously. Primary tumors with two pathogenic mutations in *APC* and no copy-number alterations at *APC* locus other than WGD were considered, resulting in n=1,305 samples (Supplementary Table 3). We also analysed the protein position of stop-gain and frameshift *APC* mutations in the cohort of CRCs reported in Christie *et al*. [21] (n =630).

#### 1.6 FAP Cohorts

Samples in published FAP datasets (including 93 adenomas from Sieber et al [22], 55 adenomas from Miyaki et al [11,12] and 86 cancer samples from Lamlum et al [12]) with known germline and somatic *APC* mutation data were classified by *APC* genotype using the same criteria. CN-LOH and CL-LOH were both classified as LOH due to resolution limitations.

#### 1.7 CMS Classification

Consensus Molecular Subtypes (CMS) were assigned to 509 TCGA CRCs based on log2_scaled Gene Expression Profiles using the CMSclassifier R package [23].

#### 1.8 AXIN2 Expression

*AXIN2* RNA expression was assessed for a subset of CRCs in the TCGA cohort (n=509, reported in RSEM normalized counts) and the 100kGP cohort (n=89, reported in Transcripts Per Million). We defined the weighted difference in *AXIN2* expression as the average difference in *AXIN2* expression between tumors with additional driver mutation in a specific WNT pathway and tumors with only *APC* mutations that retained the same number of 20AARs, weighted by the number of samples with each number of 20AARs.

### 2 Mathematical Model of APC-driven CRC Initiation

We propose a mathematical model of *APC*-driven CRC initiation to estimate the relative probability of a given *APC* genotype resulting in tumor progression. We first outline the model, before detailing how it is parameterised and used for inference. Greater mathematical detail of the model can be found in Supplementary Notes 1-2, but is not required for the analysis presented in the main text.

#### 2.1 Defining the CRC progression probability of APC genotypes

##### 2.1.1 Mutation accumulation

We consider all major types of mutations leading to APC inactivation: stop gained or frameshift mutations in regions *R_0_, R_1_, R_2_* or *R_3_* (referred to as type 0, 1, 2, or 3 mutations, respectively), copy loss of an allele (CL-LOH, denoted by ‘-’); and copy neutral loss of an allele (CN-LOH, denoted by type ‘x2’). Consider a colonic cell with both wild-type alleles for *APC*, labeled by [W,W]. Given that it gets a first *APC* mutation, it is of type *i* with probability *m_i_*. We label the cell with a single mutation of type *i* by [i,W]. The first *APC* mutation is fixed in the crypt with a probability that is independent of its type. If a second *APC* mutation occurs, it is of type *j* with probability *m_j_*, where we assume that the second mutation has the same probabilities of being type *j* as the first mutation, and that this occurs independently of the type of the first mutation. Thus, given that a cell has accumulated two *APC* mutations, these are of type *i* and type *j* respectively, with probability *m_i_ m_j_*. We label the biallelic mutant cell by the ordered pair [i,j]. This two-step process can be summarised as:

$$[\text{W},\text{W}]\to [\text{i},\text{W}]\text{with}\;\text{probability}m_{i},[\text{i},\text{W}]\to [\text{i},\text{j}]\text{with}\;\text{probability}m_{j}.$$

In principle, arbitrarily long mutation paths leading to APC inactivation might exist. However, due to the mutation rates being small (*μ_APC_* ≈ 6.22 10^-6^, Methods), long paths are unlikely, and so we consider only mutation paths of length 2. Moreover, we disregard: CN-LOH as the first mutation event; CN-LOH following CL-LOH; and, double CL-LOH, i.e. [-,-] as these genotypes are unobserved in cancer data. For other mutation events we ignore the order of mutations. Thus, we assign the unordered pair (*M,N*) as the genotype label as in Table M2, calculate the probabilities of the genotype (*M,N*) in terms of *m_i_ m_j_*, and normalise these to sum to one. Specifically, the probability that that a cell with bi-allelic inactivation of *APC* has genotype (*M,N*) is:

(Equation M1) $$m_{\left(M,N\right)}\propto Km_{M}m_{N},$$

where K=1 if *N*∈{*M*,x2} and K=2 otherwise due to genotype labelling and the proportionality is due to normalising. Under the assumption that the first hit’s effect is independent of its mutation type, Equation M1 holds independently of the population dynamics, and is true both for the first arrival of a double mutant cell in the population and for any subsequent double mutant *APC* arrival.

##### 2.1.2 CRC progression

We assume that cells with biallelic APC inactivation can stochastically progress to become a detectable CRC with a probability that depends on the *APC* genotype (*M,N*), denoted by *p_(M,N)_*. Then, the probability that a detected CRC has genotype (*M,N*) is

(Equation M2) $$f_{\left(M,N\right)}=\frac{1}{c}m_{\left(M,N\right)}p_{\left(M,N\right)}$$

where *C* is constant across genotypes, although it could be age dependent (more detail in Supplementary Note 1). The constant *C* cancels out when calculating the relative CRC progression probability of *APC* genotype (*M,N*), defined as

(Equation M3) $${\tilde{p}}_{\left(M,N\right)}=\frac{p_{\left(M,N\right)}}{\sum_{i,j}p_{\left(i,j\right)}}=\frac{f_{\left(M,N\right)}/m_{\left(M,N\right)}}{\sum_{i,j}f_{\left(i.j\right)}/m_{\left(i,j\right)}}.$$

Similarly, the relative progression probability of *X* retained 20AARs is given by

(Equation M4) $${\tilde{p}}_{X}=\frac{f_{X}/m_{X}}{\Sigma_{y=0}^{6}f_{y}/m_{y}}$$,

where *m*_*X*_ *=* ∑_(*i, j*):*X*_ *m*_(*i, j*)_ and *f*_*X*_ *=* ∑_(*i, j*):*X*_ *f*_(*i, j*)_*f*are calculated by summing over genotypes that retain *X* 20AARs (determined as in Table M2), and in the denominator we summed over all possible values of 20AARs retained, from 0 to 6. The same arguments allow us to calculate the relative progression probability of retaining the first 15AAR repeat (Supplementary Note 3).

##### 2.1.3 CRC progression probabilities in FAP patients

FAP patients harbour germline mutations in APC, thus CRC progression requires a single mutation on the non-mutated allele. Under the model above, in patients with germline mutation in region R_M_, the probability that, if they develop CRC, this has APC genotype (M,N) is

(Equation M5) $$m_{N}p_{\left(M,N\right)}/(\sum_{i=0,1,2,3}m_{i}p_{\left(M,i\right)}+m_{-}p_{\left(M,-\right)}+m_{x2}p_{\left(M,x2\right)})$$

##### 2.1.4 Progression-weighted mean 20AARs number

For a subset of tumors *A*, we define the progression-weighted mean 20AARs number as

(Equation M6) $$\Sigma_{x=0}^{6}\;x{\tilde{p}}_{x,A}$$

where ${\tilde{p}}_{{\mathrm{X,}}_{A}}$ denotes the relative progression probability of *X* retained 20AARs calculated using the mutational processes and genotype frequencies of a subset *A* of tumors. We compare disjoint subtypes of tumors *A* and *B* by computing the difference

(Equation M7) $${\text{Δ}}_{A-B}:=\Sigma x=06\;x{\tilde{p}}_{x,A}-\Sigma x=06x{\tilde{p}}_{x,B}.$$

#### 2.2 Estimating mutation and genotype probabilities

We now outline our method to estimate the probability that cells with biallelic APC inactivation have genotype (*M,N*) under mutational processes alone, *m*_(M,N)_. As Eq. M1 holds to a normalization factor, it is enough to estimate *m*_i_/*m*_0_ for i=0,1,2,3,-,x2. More technical details can be found in Supplementary Note 4.

##### 2.2.1 Truncating mutations in region R_j_

To estimate the relative probability of truncating mutations across *APC* regions in MSS samples, we first assessed the likelihood of a truncating mutation being a stop-gain versus a frameshift. Based on healthy colonic crypt data [24] we assumed a ratio of 24:1 single-base-substitutions (SBS) to small insertions or deletions (indels), and estimated that ~5.2% of SBSs produced stop-gain mutations and 88% of indels caused frameshifts. This yields a stop-gain to frameshift ratio of approximately 15:11, giving p_stop-gain_=0.58, p_frameshift_=0.42. Next, we used COSMIC-defined [25] mutation types to compute the probability that a new mutation falls in each *APC* region for stop-gain and frameshifts separately. SBS mutations were classified into standard COSMIC 96 types; indels were grouped into 71 COSMIC types (excluding microhomology types, which represent <5% of indels in colonic crypts [24]). To obtain the probability of each mutation type occurring, we used mutational signature exposures in healthy colonic crypts from Lee-Six *et al*. [24], including only ubiquitous signatures present in >85% of samples (SBS1, SBS5, SBS18 for SBS; ID1, ID2, ID5 for indels). The per-sample signature exposures were normalized to sum to 1, and we then averaged across samples for the average exposure frequencies. For each mutation type, we divided its probability by the total number of compatible loci in APC, defined as the loci in which the mutation type could occur. Summing over all compatible loci and mutation types in region *R_j_* that would result in a truncating mutation, we used signatures and the average exposure frequencies to estimate P(stop-gain in *R_j_*) and P(frameshift in *R_j_*) for each region. These were summed weighted by p_stop-gain_ and p_frameshift_ to obtain the overall regional weight *m_j_* that a truncating mutation falls in region *R_j_*. Taking the ratios of the regions *R_j_* and *R_0_* we get *m_j_/m_0_* for *j*=0,1,2,3.

##### 2.2.2 Copy number alterations

We assumed that genotypes (0,0), (0,-) and (0,x2) have the same progression probability. From Eq. M2, the probabilities *m*_*(0,-)*_, *m*_*(0,x2)*_ can be obtained from the ratios of the probabilities of CRCs with the corresponding genotypes, which we estimate as the frequencies of cancers with the given genotype from cohort sequence data to obtain

(Equation M8) $$\frac{m_{\left(0,-\right)}}{m_{\left(0,0\right)}}=\frac{2m_{-}}{m_{0}}=\frac{f_{\left(0,-\right)}}{f_{\left(0,0\right)}}\approx 1.86\text{and}\frac{m_{\left(0,\times 2\right)}}{m_{\left(0,0\right)}}=\frac{m_{x2}}{m_{0}}=\frac{f_{\left(0,\times 2\right)}}{f_{\left(0,0\right)}}\approx 1.43,$$

where we used Eq. M1 to relate the *m*_(*M,N*)_ and *m_i_* terms. This gives *m*_i_/*m*_0_ for *i*∈{-,x2}, and thus all parameters needed to calculate the mutation probabilities *m_(M,N)_* for all *APC* genotypes.

##### 2.2.3 Mutation probabilities in proximal-distal comparison and hypermutant samples

We stratified the healthy crypt signature data in [24] by proximal and distal colon to calculate anatomical-site specific mutation probabilities *m*_j_ for *j*=0,1,2,3. To estimate the mutation probabilities in *POLE*-deficient tumors, we used signature data from individuals with germline *POLE* mutations [26] and set *p*_frameshift_ = 0 since none of the *POLE*- deficient CRCs in our cohort had frameshift mutations in *APC*. For MSI, we used the signatures present in >85% of MSI CRCs in the 100kGP (Supplementary Tables 4-5), determined by [3]. As the ratio of SBS to indels was 10:1 in MSI CRCs, resulting in 5:9 stop-gain to frameshifts, we estimated *p*_stop-gain_ = 3/14, and *p*_frameshift_ = 9/14.

##### 2.2.4 Mutation rates

Using an estimated rate of 1.45 · 10^-8^ substitutions per base-pair per year in colonic cells [24], 4,717 base pairs in *APC* upstream the SAMP repeat, with ~5.2% of SBSs producing stop codons and a stop-gain to frameshift ratio of 15:11, we computed the rate of truncating *APC* mutations as

$$\mu_{APC}=1.4510^{-8}\cdot 4717\cdot 0.052\cdot (1+11/15)\approx 6.22\cdot 10^{-6}\text{per}\;\text{cell}\;\text{per}\;\text{year}.$$

For region *R_0_*, this gives *μ_0_* ≈ 5.28 · 10^-6^. Using the frequencies of tumors with complete APC loss in Eq. M8, the rates of copy-loss LOH and copy-neutral LOH in APC in healthy crypts are given by

$$\mu_{-}=\frac{f_{\left(0,-\right)}}{f_{\left(0,0\right)}}\cdot \frac{\mu_{0}}{2}\approx 4.72\cdot 10^{-6}\text{and}\mu_{x2}=\frac{f_{\left(0,x2\right)}}{f_{\left(0,0\right)}}\cdot \mu_{0}\approx 7.18\cdot 10^{-6}\text{per}\;\text{cell}\;\text{per}\;\text{year}.$$

These are compared to previous estimates in the literature in Supplementary Note 5. Assuming a Poisson process model of mutation accumulation, these rates allow estimation of the expected number of arrivals of double *APC* mutant cells by time *t*, which follows

(Equation M9) $$\text{Λ}(t)=n_{s}^{2}Np_{f}t^{2}(\mu_{APC}^{2}+2\mu_{-}\mu_{APC}+\mu_{x2}\mu_{APC})/2$$

where *N* is the total number of crypts in the colon, *n_s_* is the number of stem cells per crypt and *p_f_* the fixation probability of a single mutation in the crypt. The derivation is detailed in Supplementary Note 2.

### 3 Statistical analysis

Statistical analysis was performed using Python 3.9 and R 4.4.0. Standard statistical tests were performed and are described in the main text and figure legends, with confidence level 95% unless otherwise stated. The following tests were designed to test the competing models for APC inactivation.

- The **“Uniform risk”** model proposes that all *APC* genotypes have the same risk of driving CRC upon correction for mutational biases. To test this, we simulate CRC cohorts of the same size as the data drawing the *APC* genotypes from the relative mutation probabilities and infer the relative progression probabilities on the simulated data. We do this K=10^4^ times and find the proportion in which the maximal difference between the inferred progression probabilities is larger than the maximal difference inferred from the data, yielding a P-value.
- The “**Maximal APC loss implies Maximal CRC risk”** model proposes that *APC* genotypes resulting in maximal APC inactivation have the largest progression probability. To test this, upon rejection of the ‘Uniform risk’, we find the 95% bootstrap CI of the number of 20AARs that result in maximal progression probability (i.e. the mode of the distribution by total number of 20AARs repeats), and reject if ‘0’ is outside the 95% CI of the mode. This assumes a single mode in the distribution of progression probabilities.

## Results

### Mathematical framework to test the ‘just-right’ hypothesis

**Figure 2:**
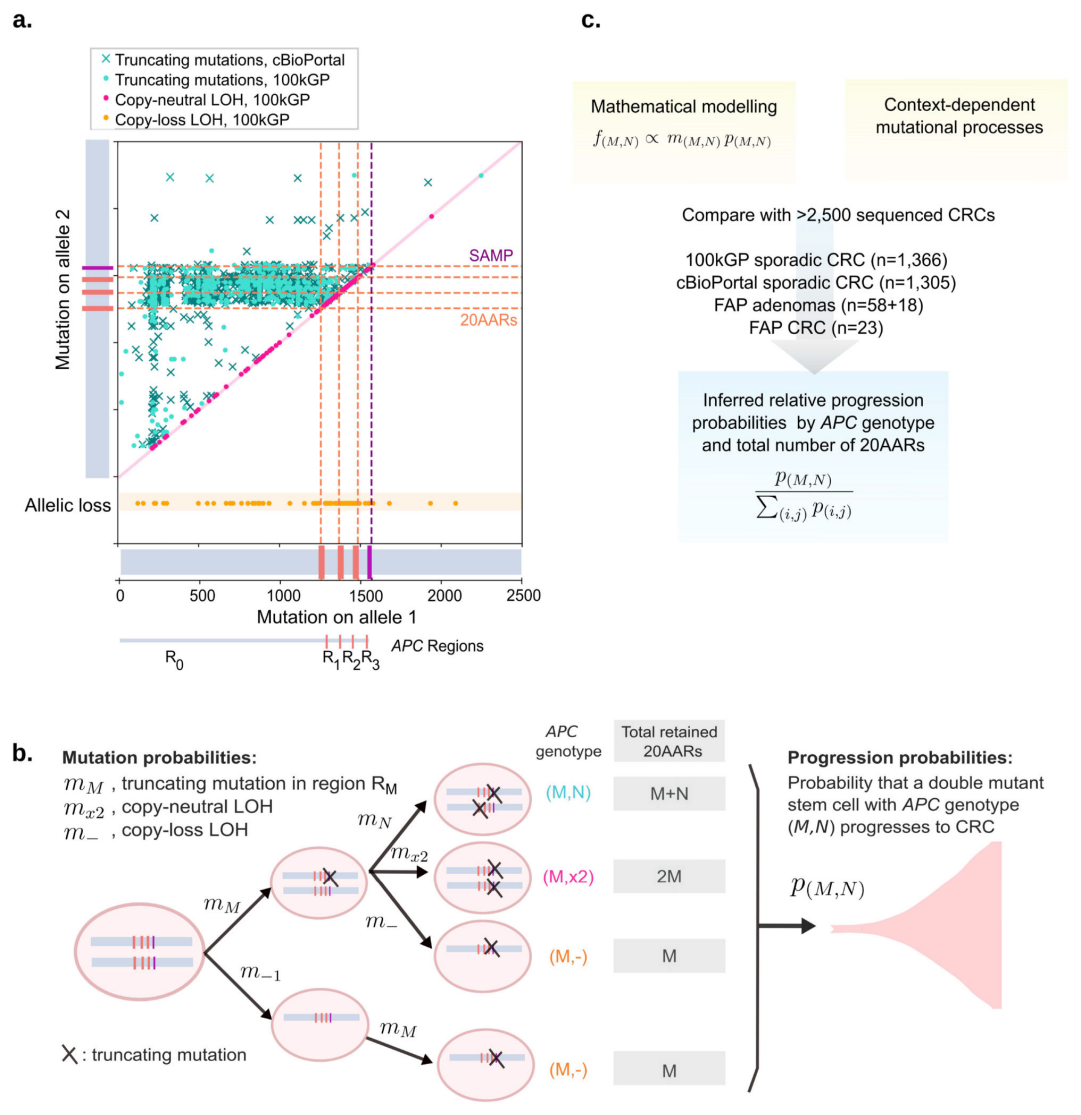
Mathematical approach to testing the ‘just-right’ hypothesis. (a) Location of *APC* truncating mutations across cBioPortal (crosses, n=1,305) and 100kGP (dots, n=1,366) CRCs with biallelic *APC* loss. Mutation closest to 5’ gene end denoted as on Allele 1 with the other mutation denoted as being on Allele 2. For cBioPortal, only tumors without copy number alterations in *APC* were considered. For 100kGP, tumors with loss of heterozygosity of *APC* via copy-neutral alteration and copy-loss of an allele, are plotted in pink and orange, respectively. The location of 20AARs and SAMP repeats is marked in dashed lines. The data displays a two-dimensional hotspot: tumors with mutations in region *R_0_* of allele 1 tend to have mutations between regions *R_1_* and *R_2_* of allele 2, and points to the 20AARs limiting the regions of interest. (b) Mathematical model of CRC initiation, in which cells accumulate truncating mutations of *APC* in region *R_M_* with probability *m_M_*, copy-loss LOH with probability *m*_-_ or copy-neutral LOH with probability *m*_*x2*_. Biallelic *APC* mutant cells are classified by the position and class of the two hits, and the corresponding total number *X* of 20AARs retained across the two alleles. Once a stem cell has lost both copies of *APC*, the cell progresses to cancer with a probability that depends on the *APC* genotype, *p_(M,N)_*. From the model, the expected frequency of cancers with a given genotype, *f_(M,N)_*, can be derived, which is comparable to cancer sequencing data. (c) Schematic of the strategy developed to infer the relative probability of progression of genotype (*M,N*), ${\tilde{p}}_{\left(M,N\right)}$, by combining mathematical modelling with sequence data from sporadic and familial *APC*-driven CRC.

We firstly profiled the distribution of mutations across the two alleles of *APC* in primary CRCs in the 100kGP and cBioPortal cohorts, which revealed a two-dimensional mutational hotspot (Figure 2a), in agreement with previous work [10]. The hotspot suggests interdependence between the first and second hit (Supplementary Figure 1), with most tumors retaining at least one 20AAR across both alleles. However, as discussed, the signal could be driven by mutational processes. To test and quantify the ‘just-right’ hypothesis for APC inactivation, and disentangle selection and mutation, we propose a mathematical framework characterising the initial stages of colorectal tumorigenesis. We first outline our mutation classification system, before detailing the mathematical model.

The *APC* genotype of colonic cells is defined by the position and class of the mutations in the two alleles. We consider all major mutation classes underlying APC inactivation: stop-gain mutations, frameshifts, and copy number alterations separated into copy-loss of heterozygosity CL-LOH, caused by the loss of the wild-type allele, and copy-neutral loss of heterozygosity, CN-LOH, where the wild-type allele is lost and the mutated is duplicated. Motivated by the observed mutational hotspots [21] and the role of the 20AARs [18,21], we classify stop-gain and frameshift mutations by regions relative to these domains and ignore mutations downstream of the first SAMP repeat (Figure 1a and Figure 2a, Methods, Supplementary Figure 2). In particular, a single truncating mutation in region *R_i_* leaves *i* intact 20AAR repeats, where *i* can be 0,1,2 or 3. *APC* genotypes are then denoted by (*M,N*), where *M* denotes a truncating mutation in region *R_M_* in one allele, and *N* either refers to a truncating mutation in region *R*_*N*_ in the other allele, or it denotes a copy-loss LOH if *N*=“-” or a copy-neutral LOH if *N*=“x2” (Figure 2b, Table 1). When there are several clonal truncating mutations, we first predict the diploid genotype of the ancestral tumor initiating cell, and then order the mutations in increasing order (*M*≤*N*) and only consider the two most upstream mutations. For example, genotype (1,2) refers to an *APC* genotype with two truncating mutations such that proteins synthesised from one allele will carry a single 20AAR, while proteins stemming from the other allele carry two 20AARs (Figure 2b).

In our model, we consider how mutation accumulation in the large bowel leads to biallelic *APC* mutated cells which, in turn, can progress to cancer (Figure 2c). To disentangle mutation and selection, we first estimate the probability *m_(M,N)_* that a biallelic *APC* mutant cell appears with genotype (*M,N*) in the absence of selection, using mutational signature data specific to the context under consideration, e.g. signatures active in healthy colonic crypts (Methods). For a given context, we assume that frameshifts and stop-gain mutations occur independently of each other, which is consistent with the genomic data (Supplementary Figure 3). The progression probability *p_(M,N)_* of *APC* genotype (*M,N*) is defined as the probability that a cell which acquired *APC* genotype (*M,N*) progresses into a detectable CRC during the patient’s lifetime. We neglect the accumulation of further mutations in *APC* after double allelic inactivation, so-called ‘third hits’ [27], as these are rare in the cohorts of study (Supplementary Figure 4). Under this framework, we show (Methods) that the expected frequency of *APC* genotype (*M,N*) in colorectal cancers is given by

$$f_{\left(M,N\right)}=Cm_{\left(M,N\right)}p_{\left(M,N\right)}$$

where *C* is independent of *APC* genotype. We estimate *f_(M,N)_* from cohort data of sequenced primary CRCs, providing access to the *relative progression probabilities*

(Equation 1) $${\tilde{p}}_{\left(M,N\right)}=\frac{p_{\left(M,N\right)}}{\Sigma_{i,j}\;p_{\left(i,j\right)}}=\frac{f_{\left(M,N\right)}/m_{\left(M,N\right)}}{\Sigma_{i,j}\;f_{\left(i,j\right)}/m_{\left(i,j\right)}}$$

which enable assessment of the tumorigenic effect of different *APC* genotypes, while controlling for mutational processes (Figure 2d). Note that we only focus on the relative probabilities as we were unable to estimate the constant C, which conveniently cancels out in Equation 1. To relate genotypes to a measure of residual APC activity, we determine the total number *X* of 20AARs retained across the two alleles for each genotype (Figure 2b) and estimate the relative progression probability of genotypes with *X* retained 20AARs, ${\tilde{p}}_{X}$.

### ‘Just-right’ APC inactivation for CRC initiation

To test and quantify the effect of different APC inactivation levels for cancer initiation, we applied our mathematical model to the 100kGP cohort [3]. Initially, we considered microsatellite-stable (MSS) primary tumors with double allelic inactivation of APC, and without pathogenic mutations of DNA polymerase epsilon (*POLE*) (n=1,037, filtering details are in Methods).

**Figure 3:**
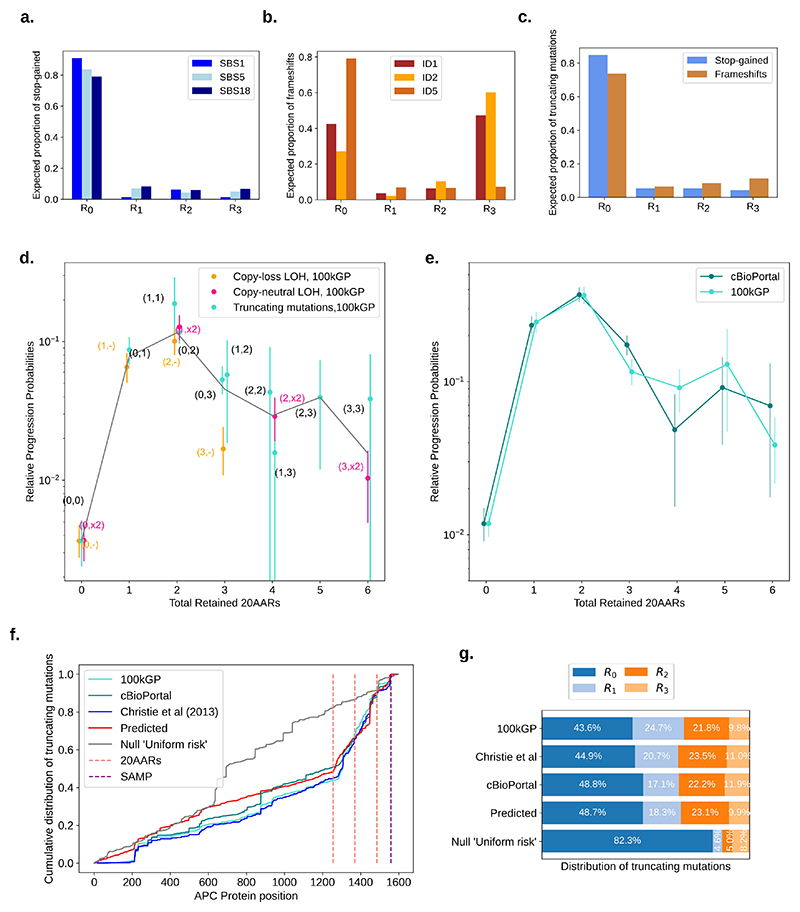
Optimal number of 20AARs for CRC progression. (a, b) The proportion of stop-gain and indels, respectively, expected to fall in different regions of *APC*, estimated by considering the ubiquitous mutational signatures found in healthy colon crypts [24]. (c) The expected proportion of truncating mutations in each region which is used to estimate the rates of truncating mutations in each region. (d) The relative progression probability of different *APC* biallelic genotypes, ${\tilde{p}}_{\left(M,N\right)}$, is plotted against the total number of 20AARs retained across both alleles. The frequencies of genotypes were calculated from sequence data of MSS primary CRCs in the 100kGP cohort (n=1,037, Methods). Whiskers represent 95% confidence intervals (bootstrapping). The grey line is the average of the progression probability over all genotypes resulting in a given number of retained 20AARs, weighted by the number of samples. (e) The relative progression probability of different total number X of 20AARs retained across both alleles of *APC*, $\tilde{p}x$, with frequencies calculated from sequence data of MSS primary CRCs in 100kGP (n=1,037, Methods) and cBioPortal (n=1,041, Methods). (f) The cumulative distribution of truncating mutations in *APC*, as observed in independent MSS CRC cohorts (cyan for 100kGP n=1,037, green for cBioPortal n=1,041 and dark blue for the cohort reported in Christie *et al*. [21] *n=630), predicted by the model with selection on the total 20AARs (red) and the null ‘Uniform risk’ model, which only considers mutational processes (grey). (g) The distribution of truncating mutations per region of APC*, as observed in CRC cohorts, and predicted by the model with selection by 20AAR and the null ‘Uniform risk’ model.

First, we parametrized the model using mutational signatures active in healthy colonic crypts [24] to estimate the probabilities of truncating mutations in different regions of *APC* under neutral evolution (Figure 3a-c). The naive expectation is that these would be proportional to the lengths of the regions. Indeed, the majority of variants are expected to occur within *R_0_*, which is the longest region (Figure 3a). However, frameshift mutations are relatively biassed to *R_3_*, due to the activity of indel signature ID2 acting on a 7-base thymine mononucleotide repeat starting at codon 1554 (Figure 3b). Assuming that genotypes retaining 0 copies of 20AARs are equally tumorigenic, the proportion of those that have copy-number alterations is informative of the relative rates of CL-LOH and CN-LOH compared to single base substitutions (SBS). By using a SBS rate in healthy colonic crypts of 1.45·10^-8^ [24], and noting that CN-LOH can only occur as a second hit, we find rates of 4.72·10^-6^/cell/year for *APC* CL-LOH and 7.18·10^-6^/cell/year for *APC* CN-LOH (Methods). Under a Poisson process model of mutation accumulation, the above rates allow estimation of the expected number of independent times in which a new colonic stem cell with biallelic APC inactivation appears, which we find to be on the order of hundreds by 80 years (Equation M9 in Methods, Supplementary Note 2).

Combining the mutation probability estimates of different *APC* genotypes with the corresponding frequencies in MSS CRCs in the 100kGP cohort, we estimated the relative progression probabilities of *APC* genotypes, ${\tilde{p}}_{\left(M,N\right)}$. Remarkably, we found that genotypes (1,1), (1,x2), (0, 2) and (2,-) have around 50 times higher progression probabilities than genotype (0,0) (Figure 3d, Supplementary Table 6). As confidence intervals were obtained by bootstrapping, large confidence intervals were obtained for rare genotypes in the cohort, i.e. for (1,2), (2,2), (1,3), (3,3). Similar results were found when analysing primary MSS CRCs in the cBioPortal cohort [2,28], where we considered biallelic diploid *APC* mutant samples (Supplementary Figure 5). For both cohorts, we rejected the ‘uniform CRC risk’ hypothesis that all genotypes have the same cancer progression risk (P=3.4·10^*-4*^, P=1.2·10^*-4*^, respectively, Methods) and the hypothesis that maximal loss of APC provides maximal CRC risk (0 not in 95% CI of mode). We found that the total number of 20AARs explains a considerable degree of variability in the relative progression probabilities between genotypes (*R^2^*=0.82 for 100kGP, *R^2^*=0.89 for cBioPortal), with 1-2 copies of 20AARs providing maximal tumorigenic effect (Figure 3e). That 1-2 20AARs provide maximal cancer risk was also found to be robust to presence or absence of *TP53, KRAS, PIK3CA* and *SMAD4* - canonical MSS drivers that are not primarily involved in WNT pathway regulation (Supplementary Table 7). Further discussion of the role of different AAR repeats and comparison with previous literature can be found in Supplementary Notes 3 and 6.

While our primary focus is on *APC* genotypes stratified by 20AAR regions, we also evaluated the degree to which our model explains CRC *APC* mutations throughout the gene. To do so, we calculated the predicted distribution of mutations in *APC* using the mutational processes model and the 20AAR progression probabilities that we estimated using the 100kGP cohort. Comparing the predicted distribution to independent CRC cohorts (Figure 3f, Supplementary Figure 6), we found that the proposed model explains most variability in the distribution of *APC* mutations observed in MSS CRCs (*R^2^*=0.942 for 100kGP, *R^2^*=0.963 for cBioPortal, *R^2^*=0.913 for the cohort in Christie *et al*. [21]), and accurately predicts the distribution of mutations by *APC* region (Figure 3g). Notably, this supports a model in which the landscape of *APC* mutations observed in MSS CRCs is primarily the result of accumulation of mutations in the healthy colon combined with selection on the total number of 20AARs in biallelic mutant cells.

Since our analysis indicates that the total number of 20AARs underlies most genotypic variability in CRCs, henceforth we focus on this as a measure of APC inactivation. To further investigate the association between APC inactivation and WNT activity ^21–24,32^, we assessed AXIN2 RNA expression level for a subset of samples of both cohorts [29]. In both cases, we found that AXIN2 expression was negatively correlated with the total number of 20AARs retained (100kGP: n=56, Pearson correlation cor=-0.34, P=3.1·10^-2^; cBioPortal: n=168, cor=-0.23, P=2.6·10^-3^, Supplementary Figure 7, Supplementary Tables 8-9), in concordance with previous work *in vitro* [16,18]. Combined with the genotypic findings provided above, these results support that a “just-right” level of WNT dysregulation leads to maximal cancer risk. However, a considerable proportion of tumors develop through ‘non-optimal’ APC inactivation levels (e.g. 14.5% of tumors in 100kGP retain 0 copies of 20AARs). Next, we studied other factors that influence WNT activity to understand the variability in CRC progression risk amongst lesions with the same APC genotypes.

### APC inactivation varies across anatomical sites

**Figure 4:**
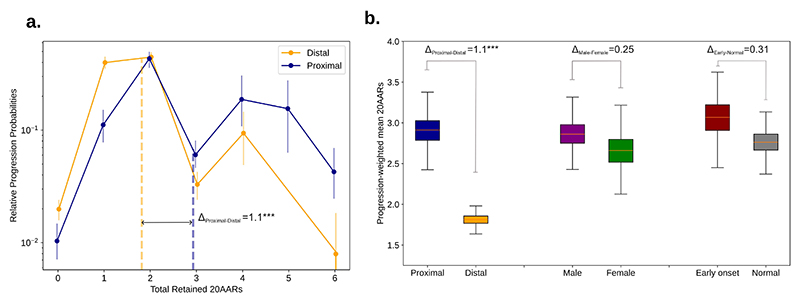
‘Just-right’ APC inactivation is higher in the distal colon. (a) The relative progression probability versus total number of 20AARs retained over both alleles, controlling for site-specific mutational processes, for proximal (blue) and distal (orange) cancers, with genotype frequencies calculated from bulk sequence data of MSS primary CRCs in the 100kGP cohort (n=313 proximal, n=574 distal/rectum). Whiskers on points indicate 95% confidence intervals (bootstrapping). Dashed vertical lines indicate the progression-weighted mean 20AARs number retained, representing the optimal level of WNT activation contributed by APC loss. Proximal tumors are under selection for a higher number of 20AARs. (b) The progression-weighted mean 20AARs number retained in different tumor stratifications, whiskers on points indicate 95% confidence intervals (bootstrapping). We find a significant difference of Δ_P-D_=1.1 between proximal and distal tumors (P<0.001, permutation test), but no statistically significant differences between tumors in male versus female patients (P=0.25, permutation test), nor in patients with early onset (<50 years old at resection) versus normal onset (>50 years old at resection) (P=0.31, permutation test).

Molecular differences between lesions in different colonic sites have been identified [21,30], which could contribute to variability in ‘just-right’ WNT levels. Mutational signature burden differs significantly across anatomical locations in both healthy crypts [24] and CRCs [3,21]. However, we found that the difference in signature proportion was relatively minor in healthy crypts (Supplementary Figure 8, Supplementary Table 10), hinting that site-specific mutational processes are unlikely to play a major role in location-specific *APC* genotype patterns. To isolate-out variability in selection, we accounted for site-specific mutational processes, and calculated the relative progression probability curves separately for both proximal and distal (including rectum) CRCs, finding significant differences between anatomical sites (Figure 4a).

To quantify differences in selection and relate them to WNT activity, we computed the progression-weighted mean 20AARs number, defined as the average number of 20AARs weighted by the corresponding progression probabilities (Methods, Equation M6). This can be interpreted as a proxy for the optimal level of WNT activation contributed by APC loss. We can then compare two subtypes of cancers A and B by calculating the difference Δ_A-B_ between their respective progression-weighted mean 20AAR number (Methods, Equation M7). If Δ_A-B_>0, the shift suggests that tumors in subtype A “prefer” to retain a higher number of 20AARs and thus require lower WNT activity, and vice versa for Δ_A-B_<0.

Using the Δ measure, we found a significant difference when stratifying tumors by anatomical site, with the progression-weighted mean 20AARs number being higher amongst proximal tumors compared to distal (Δ_Proximal-Distal_=1.1, P<0.001, permutation test) (Figure 4a). This suggests that tumors in the proximal colon have lower WNT activation due to APC loss. We considered other clinical features reported in the 100kGP cohort that could underlie variability of *APC* genotypes, but found no significant differences (permutation test, P>0.05) upon stratifying by sex or early onset cancers, defined as <50 years old at resection (Figure 4b, Supplementary Figure 9-11).

Finally, for a subset of CRCs (TCGA, n=509), we stratified tumors by consensus molecular subtype (CMS) [23]. We found no major differences across subtypes, except for a higher progression-weighted mean 20AAR within the CMS3 subtype (associated with KRAS signalling) compared to CMS2 (the canonical WNT subtype) (Δ_CMS2-CMS3_=-0.55, P=0.0048, Supplementary Figure 12, Supplementary Table 11). A potential interpretation is that CRCs with suboptimal APC inactivation progress via alternative pathways, e.g. increased KRAS signalling, in line with recent work suggesting pathway reciprocity between APC–MYC and KRAS pathways [31].

### Secondary WNT drivers can combine with APC inactivation to achieve ‘just-right’ WNT signalling

**Figure 5:**
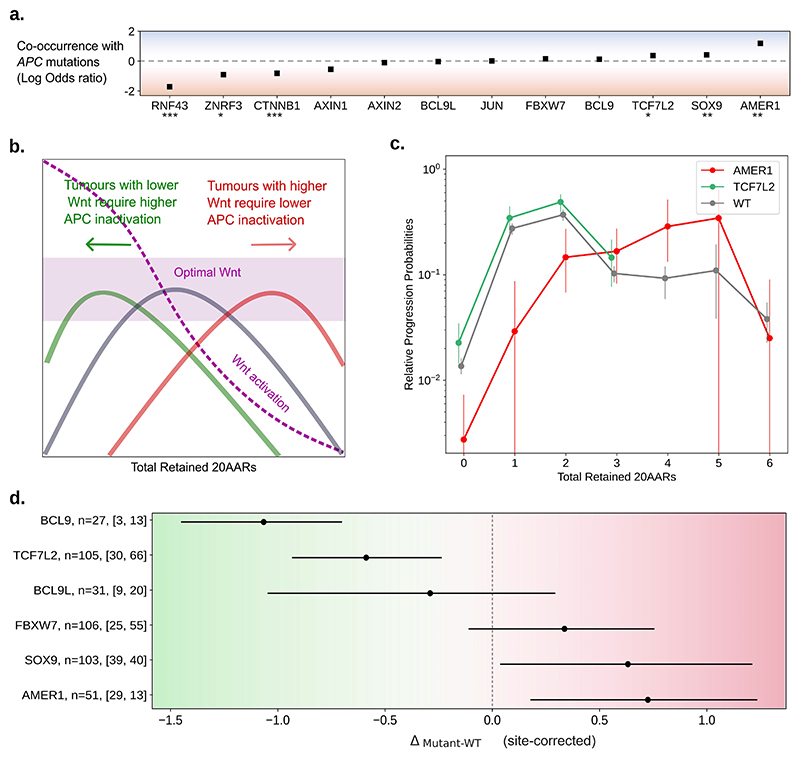
‘Just-right’ WNT activity at the pathway level. (a) Odds-ratio between APC inactivation and pathogenic mutations in other WNT related genes, in the 100kGP MSS cohort (n=1,639, Supplementary Table 12, Fisher’s test,*: P<0.05, **: P<0.01,***: P<0.001). (b) Schematic of the effect of additional mutations in WNT pathway regulators. Assuming that the cancer progression probabilities of *APC* mutant cells are due to the corresponding level of WNT pathway activation, tumors with WNT upregulating mutations will require a smaller WNT contribution from *APC* mutations, and so will have relative progression probability curves shifted to the right, and vice-versa. (c) Relative progression probabilities as a function of the total number of retained 20AARs, using sequence data of MSS primary CRCs with pathogenic *AMER1* mutations (n=51, red), with *TCF7L2* mutations (n=105, green), and tumors without mutations in non-*APC* WNT regulators (n=825 grey). Whiskers for 95% CI (bootstrapping), thick dashed vertical lines indicate the progression-weighted mean 20AARs number retained. (d) Difference in progression-weighted mean 20AARs number for tumors with pathogenic mutations in different WNT genes, Δ_mutant-WT_, corrected by the effect of anatomical site by taking the weighted average of the difference conditioned on the anatomical site of the tumors. Horizontal bars for 95% confidence intervals (bootstrapping). Numbers next to the gene labels indicate the total number of tumors with mutations in the WNT driver, and the number of which were classified as proximal and distal colon, respectively. We excluded AXIN1, AXIN2 and JUN as there were not enough samples to perform the site-correction (Supplementary Table 12 and 13).

While *APC* is the main WNT driver in CRC, other genes also dysregulate the WNT pathway when mutated [32,33]. Thus, we next investigated tumors with additional mutations in WNT drivers to study the ‘just-right’ hypothesis at the pathway level. Primary WNT drivers, such as *RNF43* or *CTNNB1*, have similarly drastic effects as APC inactivation on WNT [34,35]. In the 100kGP cohort, alterations of *RNF43* or *CTNNB1* are found in a minority of sporadic CRCs, mostly microsatellite unstable (MSI), and are mutually exclusive with APC inactivation in MSS tumors (OR=0.019, P=3.94·10^-24^ and OR=0.15, P=1.46·10^-5^ respectively, Figure 5a, Supplementary Table 12), suggesting an upper bound on WNT activity. Other WNT drivers with smaller effects on WNT activity can co-occur with primary WNT drivers - these are referred to as secondary WNT drivers [36]. In 100kGP, driver mutations of *AMER1, SOX9* and *TCF7L2* co-occur with *APC* in MSS tumors (OR=15.53, P=2.34·10^-5^; OR=2.62, P=7.20·10^-4^ and OR=2.35, P=1.47·10^-3^, respectively, Figure 5a, Supplementary Table 12). These genes have also been found mutated in precancerous lesions [37,38], emphasising their tumorigenic effect in combination with canonical WNT drivers. However, how they act in concert with *APC* alterations to attain just-right WNT, and even their directional effect - i.e. whether they increase or decrease WNT activity - is unclear.

By comparing MSS CRCs with and without secondary WNT driver mutations, we reasoned that the effect of the secondary WNT drivers could be measured under the following rationale. Under the ‘just-right’ model for WNT activity, secondary WNT driver mutations that cause increased WNT are expected to be more frequent in combination with *APC* genotypes that lead to relatively low WNT, i.e. those that retain more 20AARs, resulting in a rightward shift in the relative progression probability curve, Δ_mutant-WT_>0 (Figure 5b). Similarly, secondary drivers that cause reduced WNT would be more common with WNT high *APC* genotypes, which retain fewer 20AARs, hence a left-ward shift in the relative risk curve is expected, with Δ_mutant-WT_<0 (Figure 5b).

In agreement with the theoretical expectation, a clear shift to the right was observed in tumors with driver (loss of function) mutations in *AMER1* (Figure 5c). This shift indicates that *AMER1* mutations tend to occur in tumors with lower than average APC inactivation, potentially increasing WNT activity to the ‘just-right’ window, in accordance with both *in vitro* and *in vivo* experiments showing that wild type AMER1 reduces WNT signalling [39]. Conversely, a shift to the left was observed in tumors with driver mutations in *TCF7L2* (Figure 5c), which all retain 0-3 copies of 20AARs.

To quantitatively classify genes into WNT up or down-regulators, we computed Δ_mutant-WT_ weighted by the proportion of mutations occurring in proximal or distal tumors, thus obtaining a metric that is independent of anatomical site-specific biases (Methods). Using this measure, we predicted mutated *AMER1* and *SOX9* as WNT up-regulators (Δ_mutant-WT_>0, 95% CI, bootstrapping), and mutations of *TCF7L2* and *BCL9*, as WNT downregulators (Δ_mutant-WT_<0, 95% CI, bootstrapping) in tumors with APC inactivation, relative to the wild type protein. Considering that most driver mutations in the genes above result in loss of function [3,28] (Supplementary Table 13), the findings are consistent with the current understanding of the wild-type proteins functions, e.g. AMER1 and SOX9 promote APC activity, acting as WNT repressors in healthy tissue, whilst TCF7L2 and BCL9L promote β-catenin transcription [40]. Mutations of *FBXW7* and *BCL9L* were consistent with no effect on the cancer progression risk of APC genotypes, although this may be due to low sample size, and variant-specific functional consequences. For *AXIN1, AXIN2*, and *JUN*, site-correction was not possible due to the limited number of samples. Seeking orthogonal evidence, we examined the change in AXIN2 expression for the collection of MSS CRCs for which both mutational and RNA-seq data was available (n=89 samples from 100kGP, n=243 samples from TCGA). For the 4 genes for which our genetic analysis identified significant associations with 20AAR number (AMER1, SOX9, TCF7L2, BCL9) we find the same direction of association in 4/4 cases using TCGA RNA seq, and 3/4 using 100kGP RNA seq; however without significance due to the small number of tumors with secondary WNT mutations (Supplementary Figures 7 and 13, Supplementary Tables 8-9). A caveat of the expression analysis is that our proposed role for the secondary WNT regulators is at tumor initiation, whilst WNT activity likely varies considerably during cancer progression [38].

### APC inactivation in hypermutant tumors

**Figure 6:**
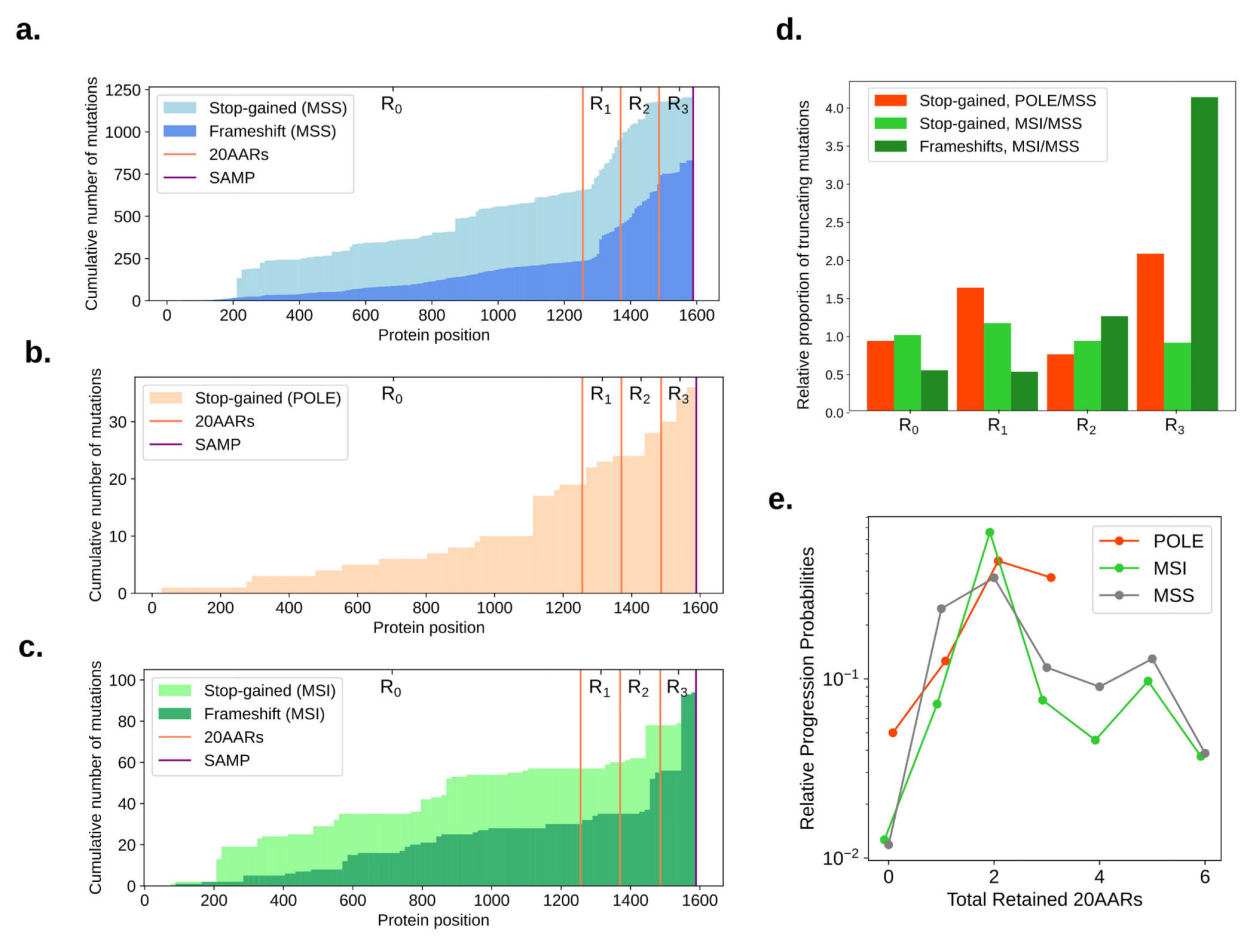
‘Just-right’ APC inactivation in POLE and MSI CRCs. (a-c) Cumulative number of stop-gain and frameshift mutations detected per codon position of *APC* in MSS (a), *POLE*-mutant (b) and MSI (c) primary CRCs in the 100kGP cohort. Vertical lines indicate the locations of the 20AAR domains and the SAMP repeat. (d) Expected proportion of mutations of different regions of *APC* in *POLE*-mutant and MSI relative to MSS, calculated using the mutational signatures detected in healthy colonic crypts [24], *POLE*-mutant crypts [26], and MSI colorectal cancers [3] (Supplementary Tables 4,5,10). (e) The relative progression probabilities by total number of 20AARs retained in MSS, *POLE*-mutant and MSI CRCs in the 100kGP cohort.

Thus far we have focused our analysis on MSS CRCs, and excluded hypermutant CRCs - that is CRCs with mutations affecting the proofreading capability of DNA polymerase epsilon (*POLE*), and microsatellite unstable (MSI) CRCs. These tumors not only have an increased mutational burden, but are also characterised by distinct mutational processes [41,42]. Thus, it is not surprising that the landscape of *APC* mutations in *POLE* and MSI CRCs in the 100kGP cohort differs from MSS CRCs (Figure 6a-c). To assess whether hypermutant CRCs comply with the ‘just-right’ distribution observed for MSS cancers (Figure 4), we first studied how the intrinsic mutational processes active in hypermutant cancers affect the distribution of *APC* genotypes, assuming that *POLE* mutations and mismatch repair deficiency precedes APC inactivation [43].

Integrating data on *POLE* mutational signatures [26] with the sequence context of *APC* (Methods), we found that *POLE*-mutant associated signatures result in an expected increased proportion of stop-gain mutations in *APC* regions *R_1_* and *R_3_* compared to healthy crypts (Figure 6d, Supplementary Table 10). Since no frameshifts in *APC* were observed in the *POLE* CRCs in 100kGP (Figure 6b), we omitted the indel analysis for these tumors. To analyse the distribution of *APC* genotypes in lesions with microsatellite instability (MSI), we used the genome-wide mutational signatures found in >85% of MSI CRCs in the 100kGP cohort [3], n=364). Notably, the combined MSI indel signature results in a 5-fold bias for frameshifts in region *R_3_* compared to MSS (Figure 6d, Supplementary Table 10). The consequence of this bias can be observed in Figure 6c, which displays a sharp increase in the number of frameshift mutations in region *R_3_*, and should lead to more retained 20AARs in MSI compared to MSS. As we have shown that proximal lesions are more likely to progress if they retain more 20AARs, the bias could explain, in part, why *APC*-driven MSI lesions tend to occur relatively often in the proximal colon compared to MSS (63:22 and 313:574 proximal:distal ratios in the 100kGP cohort, respectively).

We analysed the distribution of *APC* mutations in CRCs in 100kGP with pathogenic *POLE* mutations or MSI, mirroring the analysis carried out for MSS CRCs (Methods). We excluded lesions with copy-number alterations, resulting in n=17 *POLE* samples and n=64 MSI CRCs. While subtle differences in the relative progression probability curves were observed (Figure 6e, Supplementary Figure 14), we again reject the hypothesis that complete APC loss provides maximal CRC risk, with 2 copies of 20AARs providing maximal risk in both in POLE-deficient and MSI CRCs (2 20AARs in the 95% CI of the mode, bootstrapping). Notably, we found no significant differences in the progression-weighted mean 20AARs number compared to MSS tumors (Δ_MSS-POLE_=-0.22, 95% CI=[-0.62, 0.15], Δ_MSS-MSI_=-0.29, 95% CI=[-0.67, 0.08], bootstrapping), whilst, without adequately correcting for the characteristic mutational signatures of *POLE* and MSI, the differences were larger, and statistically significant in the case of MSI tumors (Δ_MSS-POLE,nc_=-0.31, 95% CI=[-0.78, 0.2], Δ_MSS-MSI,nc_=-1.87, 95% CI=[-2.51, -0.81], Supplementary Figure 14). This finding emphasises the importance of mutational bias analysis, and suggests that repair deficiencies are indeed acquired prior to APC inactivation.

### ‘Just-right’ APC mutations in FAP

Evidence for the ‘just-right’ hypothesis for *APC* mutations initially came from familial adenomatous polyposis (FAP) patients, carrying germline APC mutations. FAP patients present with large numbers of polyps at early ages, and in most cases develop CRC unless treated. In agreement with the ‘just-right’ hypothesis, the somatic hit depends on the germline *APC* mutation, with most lesions retaining 1-3 copies of 20AARs [12,13]. Moreover, FAP patients show variable polyp burdens and time of cancer onset depending on the specific germline mutation [12,13].

**Figure 7:**
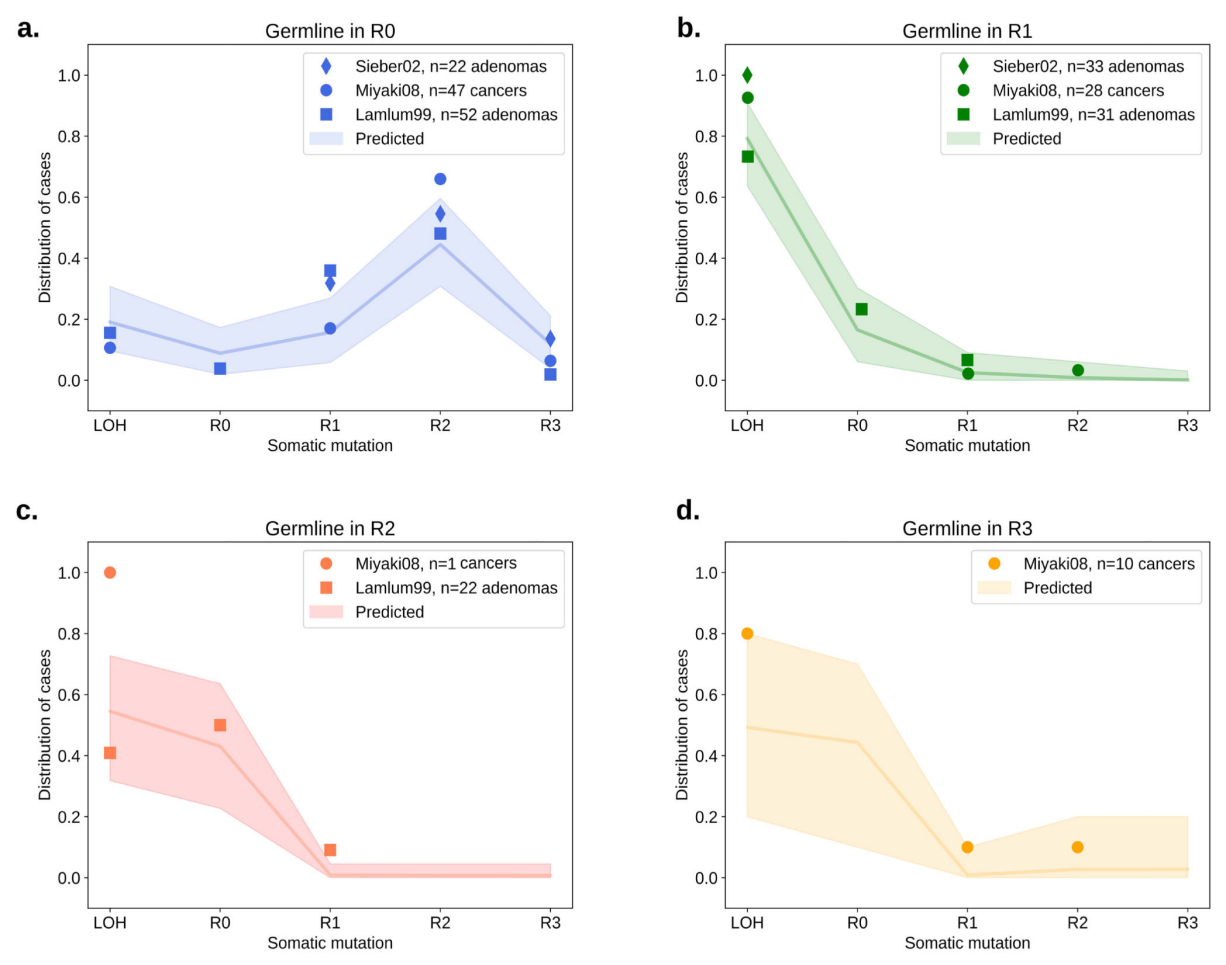
‘Just-right’ in FAP patients. (a-d) The distribution of the *APC* somatic hit in tumors of FAP patients with germline mutations in different regions. Points indicate the observed distribution in FAP patients from different studies [11,12,22], which collected 93 adenomas, 86 cancer and 55 adenomas samples from 53, 23 and 18 FAP patients, respectively. The line indicates the expected distribution calculated using the mutation and cancer progression probabilities estimated from healthy crypts and sporadic CRC data, with shaded regions for a conservative 95% CI, obtained by performing multinomial simulations with number of trials given by the maximal number of patients across the studies in each germline group.

We used the progression probabilities inferred from the analysis of sporadic MSS CRC to assess the concordance between sporadic and FAP tumors. In Figure 7, the expected distribution of the somatic *APC* hit is compared to the observed distribution in FAP patients from public datasets reported in Sieber *et al. [22]*, Miyaki *et al*. [11] *and Lamlum et al*. [12] *(Methods). For FAP patients with germline mutations in regions R_1_, R_2_* and *R_3_*, the FAP data is consistent with the expected distribution, with most adenomas and CRCs developing via LOH or mutations of region *R_0_*, (Figure 7b-d). However, for patients with germline mutations in *R*_*0*,_ the sporadic CRC distribution underestimates the proportion of lesions that develop via mutations in *R_2_*, whilst the proportion of tumors with LOH is underestimated in patients with germline in *R_1_* or *R_2_*. The discordance might exist for several reasons. A recent study suggests that cooperation between founder clones with different *APC* mutations can achieve ‘just-right’ conditions for tumor development [31], hence disagreement with the ‘just-right’ model might be due to polyclonality of some polyps in FAP. We also note unmeasured factors in the FAP data including anatomical site of the lesion or mutations in secondary WNT drivers, which both affect optimal *APC* genotypes as discussed above; and that different selective pressures might exist in FAP patients further enhancing the selective pressure for 1-2 retained 20AARs, e.g. due to intercrypt competition. However, the overall concordance between the predicted and observed distribution of the somatic hit suggests similar selection for intermediate APC inactivation in FAP tumors.

## Discussion

Repression of APC function is the canonical tumorigenic event in colorectal cancer, resulting in the WNT pathway dysregulation that is a pervasive feature of this cancer type. The tumor suppressor activity of APC relies crucially on its 20 amino acid repeat domains (20AARs), which binds and targets β-catenin as an essential part of the β-catenin destruction complex. By integrating somatic and cancer datasets with mathematical modelling we find a quantitatively consistent signal that biallelic *APC* genotypes which retain intermediate β-catenin binding activity confer maximal tumorigenic effect. This signal holds across microsatellite stable (MSS) and hypermutant sporadic CRCs, as well as FAP patient tumors. While a degree of variability in the fitness conferred by specific mutations of oncogenes is expected, e.g. G12D variants compared to A146T in *KRAS* [44], that complete loss of the β-catenin-binding 20AARs in tumor suppressive APC does not lead to maximal cancer risk is remarkable.

We find that the majority of variability in *APC* mutations results from context-dependent mutational processes in combination with selection on the total number of 20AARs across both alleles. For MSS CRCs, we found that cells with biallelic *APC* genotypes resulting in 2 retained 20AARs are at 50 times higher probability of progressing to CRC than those in which all binding domains are lost. Although prior experimental work has found that β-catenin binding strengths differ across the 20AAR domains [45],[18], we propose that *APC* mutations primarily influence fitness through the cumulative number of 20AARs. Moreover the negative correlation that we observed between retained 20AAR number and AXIN2 expression, suggests that the *APC* genotype acquired early in tumorigenesis has lasting consequences on WNT levels throughout tumor progression.

Quantitatively strengthening prior observations [21], we found that proximal and distal tumors are under selection for different levels of APC inactivation, with an effect independent of site-specific mutational processes. In particular, proximal tumors retain more 20AARs, suggesting that lower WNT pathway activation is sufficient for progression, agreeing with prior murine studies [30]. We further showed that microsatellite-instability (MSI) associated mutational processes favour *APC* alleles with higher 20AAR counts; given the reduced WNT requirements of proximal tumors, this could explain the enrichment of APC-driven MSI tumors in the proximal colon. The selection for different levels of WNT across the colon could be partly explained by the higher baseline expression of WNT genes in the proximal colon [30,46], and/or might indicate that lesions in the distal colon require higher WNT to progress, e.g. due to enhanced immune surveillance [47].

Our WNT pathway-level analysis suggests that secondary WNT drivers contribute to ‘just-right’ by increasing or decreasing the WNT dysregulation induced by *APC* mutations. In particular, we find that pathogenic mutations of *AMER1* are associated with *APC* genotypes that result in lower WNT activation, suggesting they upregulate WNT, consistent with experimental work [39,48]. Conversely, on aggregate, mutations in *TCF7L2* are associated with *APC* mutations that result in high WNT activation, suggesting they act to downregulate WNT. Thus, the combined genotype over these WNT drivers fine-tunes the necessary pathway activity, in line with the mini-driver model [49]. Whilst genetic and expression data support the proposed role of the secondary WNT regulators, experimental models combining biallelic APC inactivation and secondary WNT mutations are required to confirm this.

Recent efforts to exploit hyperactivation of cancer pathways for therapeutics [50] underscore the importance of quantifying mutation-specific effects on pathway dysregulation. By integrating somatic and cancer mutation data, we have quantified the relative fitness of ‘just-right’ APC loss. However, the ‘just-right’ effect should not be interpreted entirely deterministically; APC inactivation outside the optimal range can still promote tumorigenesis, albeit at reduced probability, or via compensatory changes in secondary WNT drivers or other oncogenic pathways [31]. The mechanisms underlying why biallelic APC-mutant cells often do not progress to detectable cancers, and the precise stage at which ‘just-right’ WNT signalling favours tumor progression, remain unclear. Comparative analyses of pre-cancerous colorectal tissue should shed light on these questions.

## Supplementary Material

Supplementary Figure 1

Supplementary Figure 2

Supplementary Figure 3

Supplementary Figure 4

Supplementary Figure 5

Supplementary Figure 6

Supplementary Figure 7

Supplementary Figure 8

Supplementary Figure 9

Supplementary Figure 10

Supplementary Figure 11

Supplementary Figure 12

Supplementary Figure 13

Supplementary Figure 14

Supplementary Notes

Supplementary Table 1

Supplementary Table 2

Supplementary Table 3

Supplementary Table 4

Supplementary Table 5

Supplementary Table 6

Supplementary Table 7

Supplementary Table 8

Supplementary Table 9

Supplementary Table 10

Supplementary Table 11

Supplementary Table 12

Supplementary Table 13

## Figures and Tables

**Table 1:** Classification of *APC* genotypes and 20AARs

Allele 1	Allele 2	Genotype	Total 20AARs
*Truncating mutation in R_M_*	*Truncating mutation in R_N_*	*(M,N)*	M+N
*Truncating mutation in R_M_*	*CL-LOH*	*(M,-)*	M
*Truncating mutation in R_M_*	*CN-LOH*	*(M, x2)*	2 M

## Data Availability

For the 100,000 Genomics England (100kGP) cohort, access to full de-identified patient data is restricted to users of the Genomics England Research Environment, and subject to a collaborative agreement that adheres to patient-led governance. For more information about accessing the data, interested readers may contact research-network@genomicsengland.co.uk. Summary tables are provided in the Supplementary Material and GitHub repository https://github.com/xellbrunet/APC_Analysis_Public. The rest of the analysis was performed on publicly available data. cBioPortal cohort data were obtained from https://bit.ly/447lpyA; Christie *et al*. cohort data was obtained from https://doi.org/10.1038/onc.2012.486 Supplementary Table 1; FAP cohort data were obtained from https://doi.org/10.1002/ijc.23390, https://doi.org/10.1038/12511 Table 1 and https://doi.org/10.1073/pnas.012679099 Table 1; mutational signature of healthy crypts data were obtained from https://doi.org/10.1038/s41586-019-1672-7 Supplementary Table 2; AXIN2 expression was obtained from cBioPortal https://bit.ly/45Eo6ZI; Gene Expression Profiles for CMS classification were obtained from TCGA via R package TCGAbiolinks. Data files and scripts are available at https://github.com/xellbrunet/APC_Analysis_Public. All other raw data are available upon request from the corresponding author.

## Significance statement

Mathematical modeling of tumor development with different APC genotypes substantiates the “just-right” APC inactivation model and suggests alterations in secondary WNT regulators enhance WNT activity in colorectal cancers with suboptimal APC genotypes.
