# Mathematical Modeling Quantifies “Just-Right” APC Inactivation for Colorectal Cancer Initiation

**DOI:** 10.1158/0008-5472.CAN-25-0445

**Published:** 2025-10-15

**Authors:** Meritxell Brunet Guasch, Nathalie A. Feeley, Ignacio Soriano, Steve Thorn, Ian P.M. Tomlinson, Michael D. Nicholson, Tibor Antal

**Affiliations:** 1 School of Mathematics and the Maxwell Institute for Mathematical Sciences, University of Edinburgh, Edinburgh, United Kingdom.; 2 CRUK Scotland Centre, Institute of Genetics and Cancer, University of Edinburgh, Edinburgh, United Kingdom.; 3 Department of Oncology, University of Oxford, Oxford, United Kingdom.

## Abstract

Dysregulation of the tumor suppressor gene adenomatous polyposis coli (*APC*) is a canonical step in colorectal cancer development by promoting activation of the WNT/β-catenin pathway. Curiously, most colorectal tumors carry biallelic mutations that result in only partial loss of APC function, suggesting that a “just-right” level of APC inactivation, and hence WNT signaling, provides the optimal conditions for tumorigenesis. Mutational processes act variably across the *APC* gene, which could contribute to the bias against complete APC inactivation. In this study, we propose a mathematical model to quantify the tumorigenic effect of biallelic *APC* genotypes, controlling for somatic mutational processes. Analysis of sequence data from >2,500 colorectal cancers showed that *APC* genotypes resulting in partial protein function confer about 50 times higher probability of progressing to cancer compared with complete APC inactivation. The optimal inactivation level varied with anatomic location and additional mutations of WNT pathway regulators. Assessment of the regulatory effects of secondary alterations in WNT drivers in combination with APC *in vivo* provided evidence that *AMER1* mutations increase WNT activity in tumors with suboptimal *APC* genotypes. The fitness landscape of APC inactivation was consistent across microsatellite unstable and polymerase epsilon–deficient colorectal cancers and tumors in patients with familial adenomatous polyposis. Together, these findings suggest a general “just-right” optimum for APC inactivation and WNT signaling, pointing to WNT hyperactivation as a potential vulnerability in cancer.

**Significance:**

Mathematical modeling of tumor development with different *APC* genotypes substantiates the “just-right” APC inactivation model and suggests alterations in secondary WNT regulators enhance WNT activity in colorectal cancers with suboptimal *APC* genotypes.

*This article is part of a special series: Driving Cancer Discoveries with Computational Research, Data Science, and Machine Learning/AI* .

## Introduction

Colorectal cancer is one of the most common and deadly cancers, with 1.9 million new cases diagnosed and 935,000 associated deaths in 2020 worldwide (1). The adenomatous polyposis coli (*APC*) gene is a canonical tumor suppressor, with loss-of-function mutations present in more than 80% of sporadic colorectal cancers (2, 3). *APC* mutations are one of the earliest, if not the earliest, genetic events in the development of colorectal cancer (4). By dysregulating the WNT signaling pathway, biallelic inactivation of APC in healthy colonic cells leads to the formation of adenomatous polyps, which can progress to carcinoma (5, 6).

**Figure 1.**
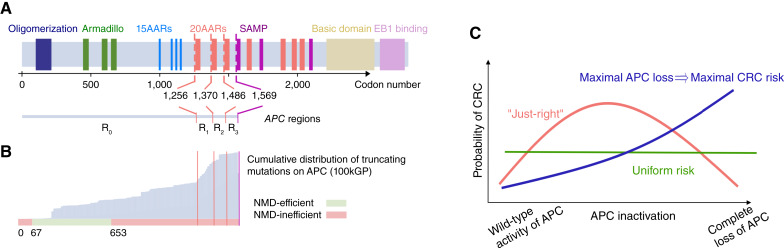
Evidence for “just-right” in sporadic colorectal cancer. **A,** Schematic showing the functional domains and regions of interest of *APC* and their corresponding codon positions. **B,** In gray, the cumulative distribution of truncating mutations of *APC* in the 100kGP cohort of colorectal cancers. Bottom, classification of codons by the efficiency of nonsense-mediated decay (NMD). Truncating mutations affecting codons in red are expected to evade NMD, as they occur either between the start codon and upstream of the 200th nucleotide or downstream of the last exon–exon junction (14). Notably, most truncating mutations occur downstream of codon 653, which are expected to evade NMD. **C,** Schematic of the “just-right” hypothesis, which posits that an intermediate level of APC inactivation maximizes colorectal cancer risk, in contrast with all genotypes conferring equal risk (“uniform risk”) and maximal APC loss conferring the maximal colorectal cancer (CRC) risk.

Wild-type APC acts as a scaffold protein for the β-catenin destruction complex, functioning as a tumor suppressor via regulation of the WNT pathway (7, 8). This activity involves several protein domains, including short repeat sequences known as 20 amino acid repeats (20AAR), which bind to β-catenin, the β-catenin inhibitory domain, and the first SAMP domain, which acts as a binding site for AXIN (Fig. 1A). *APC* is a classic tumor suppressor gene, requiring both alleles to be mutated for loss of function. Upon biallelic inactivation, APC loss leads to the stabilization and accumulation of β-catenin in the cytoplasm, which, upon translocation to the nucleus, upregulates the WNT pathway and feeds the affected cells with a permanent mitogenic signal (6).

Most sporadic colorectal cancers carry mutations occurring upstream of the first SAMP repeat (codon 1569) but retain some of the 20AARs (9, 10). A similar pattern has been observed in tumors of patients with familial adenomatous polyposis (FAP), in which germline mutations removing all 20AARs are typically followed by somatic second-hit mutations that retain at least one 20AAR (11–13). The 20AAR domains are within the large final translated exon of *APC* (codons 653–2,843), in which stop-gain or frameshift mutations evade nonsense-mediated decay (14), resulting in the synthesis of truncated proteins with attenuated β-catenin binding activity (Fig. 1B; ref. 15). Progressive retention of APC regulatory repeat sequences has been associated with a successive decrease in WNT signaling in various experimental model systems (16, 17). In particular, mutations upstream of the first 20AAR (codons 0–1,256) result in maximal constitutive WNT activity (18, 19) but are rarely observed in colorectal tumors. Though the naïve expectation is that complete loss of a tumor suppressor gene’s function should be optimal for tumorigenesis, in most lesions, APC is not fully inactivated.

These observations have led to the “just-right” signaling hypothesis (Fig. 1C), which states that both *APC* alleles are selected to retain sufficient β-catenin regulatory activity to generate an optimal WNT signaling level for tumor growth (12, 13). Although the genetic data are consistent with the “just-right” hypothesis (9, 10, 13), context-specific mutational processes—which could not be evaluated in the original “just-right” studies—shape the mutation distribution observed in tumors and can cause mutation hotspots. For example, genomic regions with mononucleotide repeats are particularly susceptible to insertions and deletions (indel; ref. 20); thus, the seven-base thymine repeat starting at codon 1554 in *APC* might explain the enrichment for truncating mutations downstream of the 20AAR domains. Hence, the selective consequences of different *APC* genotypes *in vivo* cannot be directly concluded from their prevalence in sequence data. To resolve this, we propose a mathematical approach that allows us to quantify the probability of colorectal cancer progression of colonic stem cells with different *APC* genotypes, controlling for the underlying mutational processes in the colon. We apply our method to analyze the cohort of colorectal cancers in the UK 100,000 Genomes Project (3), hereafter 100kGP, which includes allele-specific copy-number alterations, providing an unprecedented opportunity to study the role of APC inactivation (*n* = 1,366). Our findings are corroborated by an independent dataset (cBioPortal cohort, *n* = 1,305). We quantitatively test “just-right” against the null “uniform risk” model, in which all *APC* genotypes provide the same selective advantage, and a model in which “maximal APC loss implies maximal risk” (Fig. 1B). Furthermore, we investigate tumor heterogeneity in relation to WNT activity based on the anatomic site of the lesion and the presence of additional mutations of secondary WNT regulators. Finally, the generality of the “just-right” effect is examined by comparison with hypermutant colorectal cancers and tumors from patients with FAP.

## Materials and Methods

### Classification of APC-driven colorectal tumors

#### Genomics England 100kGP

We analyzed version 5 of the UK 100kGP, which performed whole-genome sequencing on 2,023 paired cancer (∼100× average depth) and normal (blood, 33×) samples from 2,017 patients with colorectal cancer (median age 69; range, 23–94; 59.4% male), as previously described by Cornish and colleagues (3). We included primary tumors with somatic pathogenic truncating *APC* mutations upstream of the SAMP repeat (amino acid position 1569) that had not received radiotherapy prior to surgery and did not harbor germline pathogenic mutations in *APC* or Lynch syndrome genes. Unless otherwise stated, we considered microsatellite stable (MSS) tumors with no pathogenic DNA polymerase epsilon (*POLE*) mutations (*n* = 1,370, Supplementary Table S1).

#### APC genotype classification

Truncating mutations (stop-gain and frameshift) were classified by regions relative to the 20AARs as follows:

- Region *R*_*0*_: codons [0, 1256] (upstream of the first 20AAR)
- Region *R*_*1*_: codons [1257, 1370] (downstream of the first 20AAR and upstream of the second 20AAR)
- Region *R*_*2*_: codons [1371, 1486] (downstream of the second 20AAR and upstream of the third 20AAR)
- Region *R*_*3*_: codons [1487, 1569] (downstream of the third 20AAR and upstream of the SAMP repeat)

Truncating mutations in regions *R*_1_, *R*_*2*_, and *R*_*3*_ were assumed to evade nonsense-mediated decay (14), resulting in the retention of 1, 2, and 3 20AARs, respectively; mutations in *R*_0_ were assumed to result in no 20AARs.

*APC* ploidy at tumor initiation was inferred using allele-specific copy number and whole-genome duplication (WGD) status from Cornish and colleagues (3), assuming that WGD occurred after APC inactivation. Given copy number at the *APC* site [a, b] (“a” for major allele and “b” for the minor allele), samples with no WGD were classified at initiation as diploid if [1,1], copy-loss loss of heterozygosity (CL-LOH) if [1,0], and copy-neutral loss of heterozygosity (CN-LOH) if [a ≥ 2,0]. Samples with WGD were classified as diploid at initiation if [a > 0, b > 0], CL-LOH if [1–2, 0], or CN-LOH if [a > 2,0].

For each sample, given *APC* truncating mutations and ploidy, we assign a biallelic genotype (*M,N*), where *M* ∊ {0,1,2,3} denotes the region of the furthest upstream truncating mutation and *N* ∊ {0,1,2,3,-,×2} is the region of a second truncating mutation, a CL-LOH (denoted by -), or a CN-LOH (denoted by ×2; Table 1; Supplementary Table S2). If more than one clonal truncating mutation per allele was detected, we considered only the most upstream. We excluded samples that could not be classified into any of the above (Supplementary Table S1). For each genotype, the total number of retained 20AARs at initiation was inferred as outlined above.

**Table 1.** Classification of *APC* genotypes and 20AARs.

Allele 1	Allele 2	Genotype	Total 20AARs
Truncating mutation in *R*_*M*_	Truncating mutation in *R*_*N*_	*(M,N)*	M + N
Truncating mutation in *R*_*M*_	*CL-LOH*	*(M,-)*	M
Truncating mutation in *R*_*M*_	*CN-LOH*	*(M, ×2)*	2 M

#### Tumor site and additional driver mutations

Tumors were classified as proximal (*n* = 359) or distal (including rectum; *n* = 620); others were excluded from site-specific analyses. Clonal driver mutations in WNT pathway genes (*AMER1*, *AXIN1*, *AXIN2*, *BCL9*, *BCL9L*, *CTNNB1*, *FBXW7*, *JUN*, *RNF43*, *SOX9*, *TCF7L2*, *ZNRF3*, *RSPO*) and major colorectal cancer drivers (*KRAS*, *TP53*) were determined as in Cornish and colleagues (3).

#### Microsatellite instability and POLE samples

Microsatellite instability (MSI) and pathogenic *POLE* mutations were classified as in Cornish and colleagues (3). We further evaluated *POLE* samples as hypermutant if they harbored >100 mutations/megabase. We considered samples with biallelic truncating *APC* mutations (*n* = 95 from a total of 360 MSI samples and *n* = 18 from 18 *POLE* samples).

#### Independent colorectal cancer cohorts

An independent cohort of *APC*-mutant colorectal cancers from cBioPortal was processed analogously. Primary tumors with two pathogenic mutations in *APC* and no copy-number alterations at the *APC* locus other than WGD were considered, resulting in *n* = 1,305 samples (Supplementary Table S3). We also analyzed the protein position of stop-gain and frameshift *APC* mutations in the cohort of colorectal cancers reported by Christie and colleagues (*n* = 630; ref. 21).

#### FAP cohorts

Samples in published FAP datasets [including 93 adenomas from Sieber and colleagues (22), 55 adenomas from Miyaki and colleagues (11, 12), and 86 cancer samples from Lamlum and colleagues (12)] with known germline and somatic *APC* mutation data were classified by *APC* genotype using the same criteria. CN-LOH and CL-LOH were both classified as LOH due to resolution limitations.

#### Consensus molecular subtype classification

Consensus molecular subtypes (CMS) were assigned to 509 The Cancer Genome Atlas (TCGA) colorectal cancers based on log_2_-scaled gene expression profiles using the CMSclassifier R package (23).

#### *AXIN2* expression

*AXIN2* RNA expression was assessed for a subset of colorectal cancers in the TCGA cohort (*n* = 509, reported in RNA-Seq by Expectation-Maximization, RSEM, normalized counts) and the 100kGP cohort (*n* = 89, reported in transcripts per million). We defined the weighted difference in *AXIN2* expression as the average difference in *AXIN2* expression between tumors with additional driver mutations in a specific WNT pathway and tumors with only *APC* mutations that retained the same number of 20AARs, weighted by the number of samples with each number of 20AARs.

### Mathematical model of APC-driven colorectal cancer initiation

We propose a mathematical model of *APC*-driven colorectal cancer initiation to estimate the relative probability of a given *APC* genotype resulting in tumor progression. We first outline the model before detailing how it is parameterized and used for inference. Greater mathematical detail of the model can be found in Supplementary Notes S1 and S2 but is not required for the analysis presented in the main text.

#### Defining the colorectal cancer progression probability of APC genotypes

##### Mutation accumulation

We consider all major types of mutations leading to APC inactivation: stop-gain or frameshift mutations in regions *R*_*0*_, *R*_*1*_, *R*_*2*_, or *R*_*3*_ (referred to as type 0, 1, 2, or 3 mutations, respectively), CL of an allele (CL-LOH, denoted by “-”), and CN loss of an allele (CN-LOH, denoted by type “×2”). A colonic cell is considered to have both wild-type alleles for *APC*, labeled as [W,W]. Given that it gets a first *APC* mutation, it is of type *i* with probability *m*_*i*_. We label the cell with a single mutation of type *i* as [i,W]. The first *APC* mutation is fixed in the crypt with a probability that is independent of its type. If a second *APC* mutation occurs, it is of type *j* with probability *m*_*j*_, where we assume that the second mutation has the same probabilities of being type *j* as the first mutation and that this occurs independently of the type of the first mutation. Thus, given that a cell has accumulated two *APC* mutations, these are of type *i* and type *j*, respectively, with probability *m*_*i*_*m*_*j*_. We label the biallelic mutant cell by the ordered pair [i,j]. This two-step process can be summarized as follows: [W,W] → [i,W] with probability *m*_*i*_ and [i,W] → [i,j] with probability *m*_*j*_.

In principle, arbitrarily long mutation paths leading to APC inactivation might exist. However, due to the mutation rates being small (*μ*_*APC*_ ≈ 6.22 × 10^−6^; Materials and Methods: Mutation rates), long paths are unlikely, and so we consider only mutation paths of length 2. Moreover, we disregard CN-LOH as the first mutation event, CN-LOH following CL-LOH, and double CL-LOH, that is, [-,-], as these genotypes are unobserved in cancer data. For other mutation events, we ignore the order of mutations. Thus, we assign the unordered pair (*M,N*) as the genotype label as in Table 1, calculate the probabilities of the genotype (*M,N*) in terms of *m*_*i*_*m*_*j*_, and normalize these to sum to one. Specifically, the probability that that a cell with biallelic inactivation of *APC* has genotype (*M,N*) is

$${\;m}_{\left(M,N\right)}\propto K\;m_{M\;}m_{N},$$ (A)

where K = 1 if *N* ∊ {*M*,×2} and K = 2 otherwise due to genotype labeling and the proportionality is due to normalizing. Under the assumption that the first hit’s effect is independent of its mutation type, Eq. A holds independently of the population dynamics and is true both for the first arrival of a double-mutant cell in the population and for any subsequent double-mutant *APC* arrival.

##### Colorectal cancer progression

We assume that cells with biallelic APC inactivation can stochastically progress to become a detectable colorectal cancer with a probability that depends on the *APC* genotype (*M,N*), denoted by *p*_*(M,N)*_. Then, the probability that a detected colorectal cancer has genotype (*M,N*) is

$$f_{\left(M,N\right)}=C{\;m}_{\left(M,N\right)}p_{\left(M,N\right)},$$ (B)

where *C* is constant across genotypes although it could be age-dependent (more detail in Supplementary Note S1). The constant *C* cancels out when calculating the relative colorectal cancer progression probability of *APC* genotype (*M,N*), defined as

$$\;{\tilde{p}}_{\left(M,N\right)}=\frac{p_{\left(M,N\right)}}{\underset{i,j}{\sum }p_{\left(i,j\right)}}\;=\frac{f_{\left(M,N\right)}/m_{\left(M,N\right)}}{\underset{i,j}{\sum }\;f_{\left(i.j\right)}/m_{\left(i,j\right)}}$$ (C)

Similarly, the relative progression probability of *X* retained 20AARs is given by

$$\;{\tilde{p}}_{X}\;=\;\frac{\;f_{X}/m_{X}}{\overset{6}{\underset{y=0}{\sum }}f_{y}/m_{y}},$$ (D)

$$\text{where}\;{\;m}_{X}=\sum_{\left(i,j\right)\;:X}m_{\left(i,j\right)}\text{ and }\;{\;f}_{X}=\sum_{\left(i,j\right)\;:X}f_{\left(i,j\right)}$$

are calculated by summing over genotypes that retain *X* 20AARs (determined as in Table 1), and in the denominator, we summed over all possible values of 20AARs retained from 0 to 6. The same arguments allow us to calculate the relative progression probability of retaining the first 15AAR (Supplementary Note S3).

##### Colorectal cancer progression probabilities in patients with FAP

Patients with FAP harbor germline mutations in *APC*; thus, colorectal cancer progression requires a single mutation on the nonmutated allele. Under the above model, if patients with germline mutation in region R_M_ develop colorectal cancer, then the cancer has *APC* genotype (*M,N*) with probability.

$$m_{N}p_{\left(M,N\right)}/\left(\underset{i=0,1,2,3}{}m_{i}{\;p}_{\left(M,i\right)}+m_{-}\;p_{\left(M,-\right)}\;+m_{\times 2}{\;p}_{\left(M,\times 2\right)}\right)$$ (E)

##### Progression-weighted mean 20AAR number

For a subset of tumor *A*, we define the progression-weighted mean 20AAR number as

$$\overset{6}{\underset{x=0}{\sum }}x{\;\tilde{p}}_{x,A},$$ (F)

where $\tilde{p}$_*X,A*_ denotes the relative progression probability of *X* retained 20AARs calculated using the mutational processes and genotype frequencies of a subset *A* of tumors. We compare disjoint subtypes of tumors *A* and *B* by computing the difference:

$$\Delta_{A-B}:=\overset{6}{\underset{x=0}{\sum }}\;x{\;\tilde{p}}_{x,A}\;-\;\overset{6}{\underset{x=0}{\sum }}x\;{\tilde{p}}_{x,B}$$ (G)

#### Estimating mutation and genotype probabilities

We now outline our method to estimate the probability that cells with biallelic APC inactivation have genotype (*M,N*) under mutational processes alone, *m*_(M,N)_. As Eq. A holds to a normalization factor, it is enough to estimate *m*_i_/*m*_0_ for i = 0,1,2,3,-,×2. More technical details can be found in Supplementary Note S4.

##### Truncating mutations in region *R*_***j***_

To estimate the relative probability of truncating mutations across *APC* regions in MSS samples, we first assessed the likelihood of a truncating mutation being a stop-gain versus a frameshift. Based on healthy colonic crypt data (24), we assumed a ratio of 24:1 single-base substitutions (SBS) to small indels and estimated that ∼5.2% of SBSs produced stop-gain mutations and 88% of indels caused frameshifts. This yields a stop-gain/frameshift ratio of approximately 15:11, giving *P*_stop-gain_ = 0.58 and *P*_frameshift_ = 0.42. Next, we used the Catalogue of Somatic Mutations in Cancer (COSMIC) defined (25) mutation types to compute the probability that a new mutation falls in each *APC* region for stop-gain and frameshifts separately. SBS mutations were classified into standard COSMIC 96 types, whereas indels were grouped into 71 COSMIC types [excluding microhomology types, which represent <5% of indels in colonic crypts (24)]. To obtain the probability of each mutation type occurring, we used mutational signature exposures in healthy colonic crypts from Lee-Six and colleagues (24), including only ubiquitous signatures present in >85% of samples (SBS1, SBS5, and SBS18 for SBS; ID1, ID2, and ID5 for indels). The per-sample signature exposures were normalized to sum to 1, and we then averaged across samples for the average exposure frequencies. For each mutation type, we divided its probability by the total number of compatible loci in *APC*, defined as the loci in which the mutation type could occur. Summing over all compatible loci and mutation types in region *R*_*j*_ that would result in a truncating mutation, we used signatures and the average exposure frequencies to estimate *P*_(stop-gain in *Rj*)_ and *P*_(frameshift in *Rj*)_ for each region. These were summed, weighted by *P*_stop-gain_ and *P*_frameshift_, to obtain the overall regional weight *m*_*j*_ that a truncating mutation falls in region *R*_*j*_. Taking the ratios of the regions *R*_*j*_ and *R*_*0*_, we get *m*_*j*_*/m*_*0*_ for *j* = 0,1,2,3.

##### Copy-number alterations

We assumed that genotypes (0,0), (0,-), and (0,×2) have the same progression probability. From Eq. B, the probabilities *m*_*(0,-)*_ and *m*_*(0,×2)*_ can be obtained from the ratios of the probabilities of colorectal cancers with the corresponding genotypes, which we estimate as the frequencies of cancers with the given genotype from cohort sequence data to obtain

$$\;\frac{m_{\left(0,-\right)}}{m_{\left(0,0\right)}}=\frac{{2\;m}_{-}}{m_{0}}=\frac{f_{\left(0,-\right)}}{f_{\left(0,0\right)}}\approx 1.86\;\text{and}\;\;\frac{m_{\left(0,\times 2\right)}}{m_{\left(0,0\right)}}=\;\frac{m_{\times 2}}{m_{0}}=\;\frac{f_{\left(0,\times 2\right)}}{f_{\left(0,0\right)}}\approx 1.43,$$ (H)

where we used Eq. A to relate the *m*_(*M,N*)_ and *m*_*i*_ terms. This gives *m*_i_/*m*_0_ for *i* ∊ {*-*,×2}, and thus, all parameters are needed to calculate the mutation probabilities *m*_*(M,N)*_ for all *APC* genotypes.

##### Mutation probabilities in proximal–distal comparison and hypermutant samples

We stratified the healthy crypt signature data in ref. 24 by the proximal and distal colon to calculate anatomic site–specific mutation probabilities *m*_j_ for *j* = 0,1,2,3. To estimate the mutation probabilities in *POLE*-deficient tumors, we used signature data from individuals with germline *POLE* mutations (26) and set *p*_frameshift_ = 0 as none of the *POLE*-deficient colorectal cancers in our cohort had frameshift mutations in *APC*. For MSI, we used the signatures present in >85% of MSI colorectal cancers in the 100kGP (Supplementary Tables S4 and S5), determined by ref. 3. As the ratio of SBS to indels was 10:1 in MSI colorectal cancers, resulting in a 5:9 stop-gain/frameshifts ratio, we estimated *P*_stop-gain_ = 3/14 and *P*_frameshift_ = 9/14.

##### Mutation rates

Using an estimated rate of 1.45 × 10^−8^ substitutions per base pair per year in colonic cells (24), 4,717 base pairs in *APC* upstream of the SAMP repeat, with ∼5.2% of SBSs producing stop codons and a stop-gain/frameshift ratio of 15:11, we computed the rate of truncating *APC* mutations as

$\mu_{APC}=1.45\;\cdot 10^{-8}\cdot 4717\cdot 0.052\cdot \left(1+11/15\right)\approx 6.22\cdot 10^{-6}$ per cell per year.

For region *R*_*0*_, this gives *μ*_*0*_ ≈ 5.28 × 10^−6^. Using the frequencies of tumors with complete APC loss in Eq. H, the rates of CL-LOH and CN-LOH in APC in healthy crypts are given by

$\mu_{-}=\frac{f_{\left(0,-\right)}}{f_{\left(0,0\right)}}\cdot \frac{\mu_{0}}{2}\approx 4.72\;\cdot 10^{-6}$ and $\mu_{\times 2}=\;\frac{f_{\left(0,\times 2\right)}}{f_{\left(0,0\right)}}\cdot \mu_{0}\;\approx \;7.18\;\cdot 10^{-6}$ per cell per year.

These are compared with previous estimates in the literature in Supplementary Note S5. Assuming a Poisson process model of mutation accumulation, these rates allow the estimation of the expected number of arrivals of double *APC*-mutant cells by time *t*, which follows

$${\;\Lambda \left(t\right)\;=n}_{s}^{2}\;N\;\;p_{f}\;t^{2}\;\left(\mu_{APC}^{2\;}+2\;\mu_{-}\;\mu_{APC}\;+\mu_{\times 2}\;\mu_{APC}\right)/2,$$ (I)

where *N* is the total number of crypts in the colon, *n*_*s*_ is the number of stem cells per crypt, and *p*_*f*_ is the fixation probability of a single mutation in the crypt. The derivation is detailed in Supplementary Note S2.

### Statistical analysis

Statistical analysis was performed using Python 3.9 and R 4.4.0. Standard statistical tests were performed and are described in the main text and figure legends, with a confidence level of 95% unless otherwise stated. The following tests were designed to test the competing models for APC inactivation.

- The “uniform risk” model proposes that all *APC* genotypes have the same risk of driving colorectal cancer upon correction for mutational biases. To test this, we simulate colorectal cancer cohorts of the same size as the data, drawing the *APC* genotypes from the relative mutation probabilities, and infer the relative progression probabilities from the simulated data. We do this K = 10^4^ times and find the proportion in which the maximal difference between the inferred progression probabilities is larger than the maximal difference inferred from the data, yielding a *P* value.
- The “maximal APC loss implies maximal colorectal cancer risk” model proposes that *APC* genotypes resulting in maximal APC inactivation have the largest progression probability. To test this, upon rejection of the “uniform risk,” we find the 95% bootstrap confidence interval (CI) of the number of 20AARs that result in maximal progression probability (i.e., the mode of the distribution by the total number of 20AAR repeats) and reject it if “0” is outside the 95% CI of the mode. This assumes a single mode in the distribution of progression probabilities.

## Results

### Mathematical framework to test the “just-right” hypothesis

**Figure 2.**
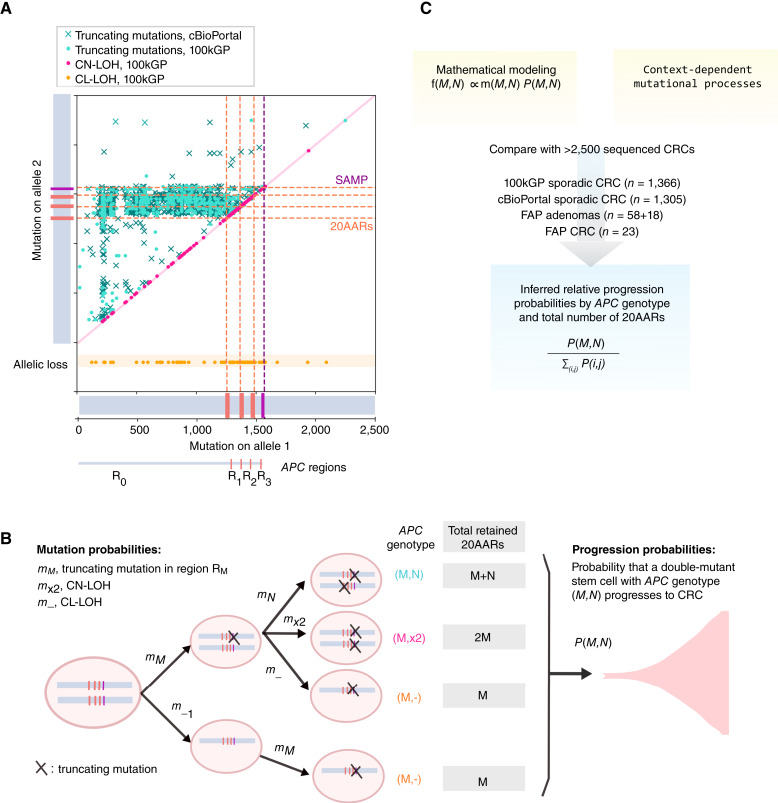
Mathematical approach to testing the “just-right” hypothesis. **A,** Location of *APC* truncating mutations across cBioPortal (crosses, *n* = 1,305) and 100kGP (dots, *n* = 1,366) colorectal cancers with biallelic *APC* loss. The mutation closest to the 5′ gene end is denoted as being on allele 1, with the other mutation denoted as being on allele 2. For cBioPortal, only tumors without copy-number alterations in *APC* were considered. For 100kGP, tumors with LOH of *APC* via copy-neutral alteration and copy-loss alteration of an allele are plotted in pink and orange, respectively. The location of 20AARs and SAMP repeats is marked with dashed lines. The data display a two-dimensional hotspot: Tumors with mutations in region *R*_*0*_ of allele 1 tend to have mutations between regions *R*_*1*_ and *R*_*2*_ of allele 2, pointing to the 20AARs limiting the regions of interest. **B,** Mathematical model of colorectal cancer (CRC) initiation, in which cells accumulate truncating mutations of *APC* in region *R*_*M*_ with probability *m*_*M*_, CL-LOH with probability *m*_*-*_, or CN-LOH with probability *m_×2_*. Biallelic *APC*-mutant cells are classified by the position and class of the two hits, and the corresponding total number *X* of 20AARs retained across the two alleles. Once a stem cell has lost both copies of *APC*, the cell progresses to cancer with a probability that depends on the *APC* genotype, *p*_*(M,N)*_. From the model, the expected frequency of cancers with a given genotype, *f*_*(M,N)*_, can be derived, which is comparable with cancer sequencing data. **C,** Schematic of the strategy developed to infer the relative probability of progression of genotype (*M,N*), $\tilde{p}$_*(M,N)*_, by combining mathematical modeling with sequence data from sporadic and familial *APC*-driven colorectal cancer.

We first profiled the distribution of mutations across the two alleles of *APC* in primary colorectal cancers in the 100kGP and cBioPortal cohorts, which revealed a two-dimensional mutational hotspot (Fig. 2A), in agreement with previous work (10). The hotspot suggests interdependence between the first and second hit (Supplementary Fig. S1), with most tumors retaining at least one 20AAR across both alleles. However, as discussed, the signal could be driven by mutational processes. To test and quantify the “just-right” hypothesis for APC inactivation and disentangle selection and mutation, we propose a mathematical framework characterizing the initial stages of colorectal tumorigenesis. We first outline our mutation classification system before detailing the mathematical model.

The *APC* genotype of colonic cells is defined by the position and class of the mutations in the two alleles. We consider all major mutation classes underlying APC inactivation: stop-gain mutations, frameshifts, and copy-number alterations, separated into CL-LOH, caused by the loss of the wild-type allele, and CN-LOH, where the wild-type allele is lost and the mutated allele is duplicated. Motivated by the observed mutational hotspots (21) and the role of the 20AARs (18, 21), we classify stop-gain and frameshift mutations by regions relative to these domains and ignore mutations downstream of the first SAMP repeat (Figs. 1A and 2A; Materials and Methods; Supplementary Fig. S2). In particular, a single truncating mutation in region *R*_*i*_ leaves *i* intact 20AARs, where *i* can be 0, 1, 2, or 3. *APC* genotypes are then denoted by (*M,N*), where *M* denotes a truncating mutation in region *R*_*M*_ in one allele and *N* either refers to a truncating mutation in region *R*_*N*_ in the other allele or denotes a CL-LOH if *N* = “-” or a CN-LOH if *N* = “×2” (Fig. 2B; Table 1). When there are several clonal truncating mutations, we first predict the diploid genotype of the ancestral tumor-initiating cell and then order the mutations in increasing order (*M* ≤ *N*) and only consider the two most upstream mutations. For example, genotype (1,2) refers to an *APC* genotype with two truncating mutations such that proteins synthesized from one allele will carry a single 20AAR, whereas proteins stemming from the other allele carry two 20AARs (Fig. 2B).

In our model, we consider how mutation accumulation in the large bowel leads to biallelic *APC*-mutated cells, which, in turn, can progress to cancer (Fig. 2C). To disentangle mutation and selection, we first estimate the probability *m*_*(M,N)*_ that a biallelic *APC*-mutant cell appears with genotype (*M,N*) in the absence of selection, using mutational signature data specific to the context under consideration, for example, signatures active in healthy colonic crypts (Materials and Methods). For a given context, we assume that frameshifts and stop-gain mutations occur independently of each other, which is consistent with the genomic data (Supplementary Fig. S3). The progression probability *p*_*(M,N)*_ of *APC* genotype (*M,N*) is defined as the probability that a cell, which acquired *APC* genotype (*M,N*), progresses into a detectable colorectal cancer during the patient’s lifetime. We neglect the accumulation of further mutations in *APC* after double allelic inactivation, so-called third hits (27), as these are rare in the cohorts of study (Supplementary Fig. S4). Under this framework, we show (Materials and Methods) that the expected frequency of the *APC* genotype (*M,N*) in colorectal cancers is given by

$$\;f_{\left(M,N\right)}=C\;m_{\left(M,N\right)}p_{\left(M,N\right)},$$

where *C* is independent of the *APC* genotype. We estimate *f*_*(M,N)*_ from the cohort data of sequenced primary colorectal cancers, providing access to the relative progression probabilities:

$$\;{\tilde{p}}_{\left(M,N\right)}=\frac{p_{\left(M,N\right)}}{\underset{i,j}{\sum }p_{\left(i,j\right)}}\;=\;\frac{\;f_{\left(M,N\right)}/m_{\left(M,N\right)}\;}{\underset{i,j}{\sum }\;f_{\left(i.j\right)}/m_{\left(i,j\right)}},$$ (J)

which enable the assessment of the tumorigenic effect of different *APC* genotypes while controlling for mutational processes (Fig. 2D). Note that we only focus on the relative probabilities as we were unable to estimate the constant C, which conveniently cancels out in Eq. J. To relate genotypes to a measure of residual APC activity, we determine the total number *X* of 20AARs retained across the two alleles for each genotype (Fig. 2B) and estimate the relative progression probability of genotypes with *X* retained 20AARs, $\;{\tilde{p}}_{X}$.

### “Just-right” APC inactivation for colorectal cancer initiation

To test and quantify the effect of different APC inactivation levels for cancer initiation, we applied our mathematical model to the 100kGP cohort (3). Initially, we considered MSS primary tumors with double allelic inactivation of APC and without pathogenic mutations of DNA *POLE* (*n* = 1,037; filtering details are in Materials and Methods).

**Figure 3.**
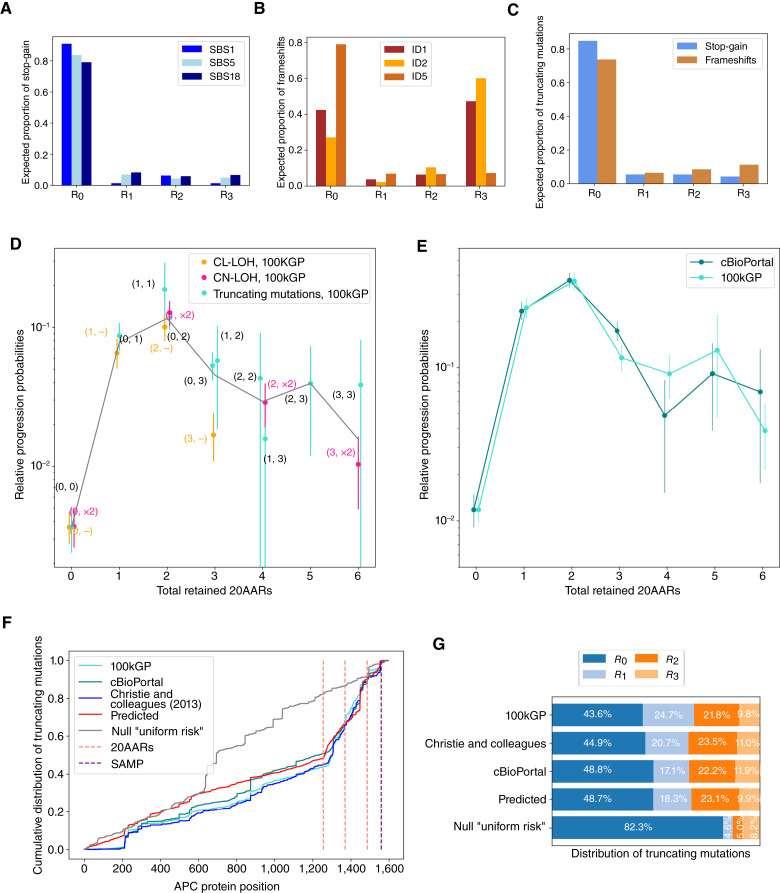
Optimal number of 20AARs for colorectal cancer progression. **A** and **B,** The proportion of stop-gain and indels, respectively, expected to fall in different regions of *APC*, estimated by considering the ubiquitous mutational signatures found in healthy colon crypts (24). **C,** The expected proportion of truncating mutations in each region is used to estimate the rates of truncating mutations in each region. **D,** The relative progression probability of different *APC* biallelic genotypes, $\tilde{p}$_*(M,N)*_, is plotted against the total number of 20AARs retained across both alleles. The frequencies of genotypes were calculated from sequence data of MSS primary colorectal cancers in the 100kGP cohort (*n* = 1,037; Materials and Methods). Whiskers represent 95% CIs (bootstrapping). The gray line is the average of the progression probability over all genotypes resulting in a given number of retained 20AARs, weighted by the number of samples. **E,** The relative progression probability of different total numbers X of 20AARs retained across both alleles of *APC*, ${\tilde{p}}_{X}$, with frequencies calculated from sequence data of MSS primary colorectal cancers in 100kGP (*n* = 1,037; Materials and Methods) and cBioPortal (*n* = 1,041; Materials and Methods). **F,** The cumulative distribution of truncating mutations in *APC*, as observed in independent MSS colorectal cancer cohorts (cyan, 100kGP, *n* = 1,037; green, cBioPortal, *n* = 1,041; and dark blue for the cohort reported in Christie and colleagues, ref. 21, *n* = 630), predicted by the model with selection on the total 20AARs (red) and the null “uniform risk” model, which only considers mutational processes (gray). **G,** The distribution of truncating mutations per region of *APC*, as observed in colorectal cancer cohorts, and predicted by the model with selection by 20AAR and the null “uniform risk” model.

First, we parametrized the model using mutational signatures active in healthy colonic crypts (24) to estimate the probabilities of truncating mutations in different regions of *APC* under neutral evolution (Fig. 3A–C). The naïve expectation is that these would be proportional to the lengths of the regions. Indeed, the majority of variants are expected to occur within *R*_*0*_, which is the longest region (Fig. 3A). However, frameshift mutations are relatively biased toward *R*_*3*_, due to the activity of indel signature ID2 acting on a seven-base thymine mononucleotide repeat starting at codon 1554 (Fig. 3B). Assuming that genotypes retaining 0 copies of 20AARs are equally tumorigenic, the proportion of those that have copy-number alterations is informative of the relative rates of CL-LOH and CN-LOH compared with SBS. By using an SBS rate in healthy colonic crypts of 1.45 × 10^−8^ (24) and noting that CN-LOH can only occur as a second hit, we find rates of 4.72 × 10^−6^/cell/year for *APC* CL-LOH and 7.18 × 10^−6^/cell/year for *APC* CN-LOH (Materials and Methods). Under a Poisson process model of mutation accumulation, the above rates allow the estimation of the expected number of independent times in which a new colonic stem cell with biallelic APC inactivation appears, which we find to be on the order of hundreds by 80 years (Eq. I; Supplementary Note S2).

Combining the mutation probability estimates of different *APC* genotypes with the corresponding frequencies in MSS colorectal cancers in the 100kGP cohort, we estimated the relative progression probabilities of *APC* genotypes, $\;{\tilde{p}}_{\left(M,N\right)}$. Remarkably, we found that genotypes (1,1), (1,×2), (0, 2), and (2,-) have around 50 times higher progression probabilities than genotype (0,0; Fig. 3D; Supplementary Table S6). As CIs were obtained by bootstrapping, large CIs were obtained for rare genotypes in the cohort, that is, for (1,2), (2,2), (1,3), and (3,3). Similar results were found when analyzing primary MSS colorectal cancers in the cBioPortal cohort (2, 28), where we considered biallelic diploid *APC*-mutant samples (Supplementary Fig. S5). For both cohorts, we rejected the “uniform colorectal cancer risk” hypothesis that all genotypes have the same cancer progression risk (*P* = 3.4 × 10^−4^; *P* = 1.2 × 10^−4^, respectively; Materials and Methods) and the hypothesis that maximal loss of APC provides maximal colorectal cancer risk (0 not in 95% CI of mode). We found that the total number of 20AARs explains a considerable degree of variability in the relative progression probabilities between genotypes (*R*^*2*^ = 0.82 for 100kGP; *R*^*2*^ = 0.89 for cBioPortal), with 1 to 2 copies of 20AARs providing maximal tumorigenic effect (Fig. 3E). That 1 to 2 20AARs provide maximal cancer risk was also found to be robust to the presence or absence of *TP53*, *KRAS*, *PIK3CA*, and *SMAD4*—canonical MSS drivers that are not primarily involved in WNT pathway regulation (Supplementary Table S7). Further discussion of the role of different AARs and comparison with previous literature can be found in Supplementary Notes S3 and S6.

Although our primary focus is on *APC* genotypes stratified by 20AAR regions, we also evaluated the degree to which our model explains colorectal cancer *APC* mutations throughout the gene. To do so, we calculated the predicted distribution of mutations in *APC* using the mutational processes model and the 20AAR progression probabilities that we estimated using the 100kGP cohort. Comparing the predicted distribution with independent colorectal cancer cohorts (Fig. 3F; Supplementary Fig. S6), we found that the proposed model explains most of the variability in the distribution of *APC* mutations observed in MSS colorectal cancers (*R*^*2*^ = 0.942 for 100kGP, *R*^*2*^ = 0.963 for cBioPortal, and *R*^*2*^ = 0.913 for the cohort in Christie and colleagues, ref. 21) and accurately predicts the distribution of mutations by *APC* region (Fig. 3G). Notably, this supports a model in which the landscape of *APC* mutations observed in MSS colorectal cancers is primarily the result of the accumulation of mutations in the healthy colon combined with selection on the total number of 20AARs in biallelic mutant cells.

As our analysis indicates that the total number of 20AARs underlies most genotypic variability in colorectal cancers, henceforth, we focus on this as a measure of APC inactivation. To further investigate the association between APC inactivation and WNT activity (21–24, 32), we assessed the AXIN2 RNA expression level for a subset of samples from both cohorts (29). In both cases, we found that AXIN2 expression was negatively correlated with the total number of 20AARs retained (100kGP: *n* = 56, Pearson correlation cor = −0.34, *P* = 3.1 × 10^−2^; cBioPortal: *n* = 168, cor = −0.23, *P* = 2.6 × 10^−3^, Supplementary Fig. S7; Supplementary Tables S8 and S9) in concordance with previous work *in vitro* (16, 18). Combined with the genotypic findings provided above, these results support that a “just-right” level of WNT dysregulation leads to maximal cancer risk. However, a considerable proportion of tumors develop through “nonoptimal” APC inactivation levels (e.g., 14.5% of tumors in 100kGP retain 0 copies of 20AARs). Next, we studied other factors that influence WNT activity to understand the variability in colorectal cancer progression risk among lesions with the same *APC* genotypes.

### APC inactivation varies across anatomic sites

**Figure 4.**
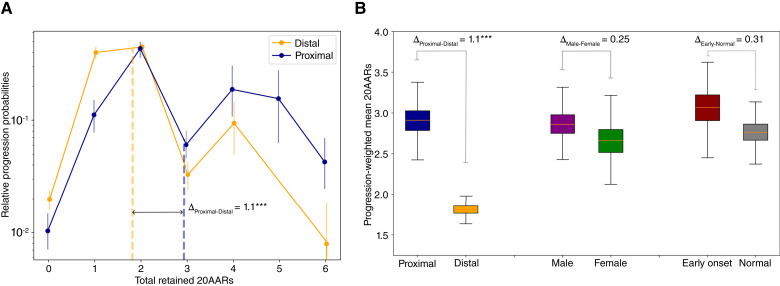
“Just-right” APC inactivation is higher in the distal colon. **A,** The relative progression probability versus total number of 20AARs retained over both alleles, controlling for site-specific mutational processes, for proximal (blue) and distal (orange) cancers, with genotype frequencies calculated from bulk sequence data of MSS primary colorectal cancers in the 100kGP cohort (*n* = 313 proximal; *n* = 574 distal/rectum). Whiskers on points indicate 95% CIs (bootstrapping). Dashed vertical lines indicate the progression-weighted mean 20AARs number retained, representing the optimal level of WNT activation contributed by APC loss. Proximal tumors are under selection for a higher number of 20AARs. **B,** The progression-weighted mean 20AARs number retained in different tumor stratifications; whiskers on points indicate 95% CIs (bootstrapping). We found a significant difference of Δ_P-D_ = 1.1 between proximal and distal tumors (*P* < 0.001, permutation test) but no statistically significant differences between tumors in male vs. female patients (*P* = 0.25, permutation test) nor in patients with early onset (<50 years old at resection) vs. normal onset (>50 years old at resection; *P* = 0.31, permutation test).

Molecular differences between lesions in different colonic sites have been identified (21, 30), which could contribute to variability in “just-right” WNT levels. Mutational signature burden differs significantly across anatomic locations in both healthy crypts (24) and colorectal cancers (3, 21). However, we found that the difference in signature proportion was relatively minor in healthy crypts (Supplementary Fig. S8; Supplementary Table S10), hinting that site-specific mutational processes are unlikely to play a major role in location-specific *APC* genotype patterns. To isolate variability in selection, we accounted for site-specific mutational processes and calculated the relative progression probability curves separately for both proximal and distal (including rectum) colorectal cancers, finding significant differences between anatomic sites (Fig. 4A).

To quantify differences in selection and relate them to WNT activity, we computed the progression-weighted mean 20AAR number, defined as the average number of 20AARs weighted by the corresponding progression probabilities (Eq. F). This can be interpreted as a proxy for the optimal level of WNT activation contributed by APC loss. We can then compare two subtypes of cancers A and B by calculating the difference Δ_A-B_ between their respective progression-weighted mean 20AAR numbers (Eq. G). If Δ_A-B_ > 0, the shift suggests that tumors in subtype A “prefer” to retain a higher number of 20AARs and thus require lower WNT activity, and vice versa for Δ_A-B_ < 0.

Using the Δ measure, we found a significant difference when stratifying tumors by anatomic site, with the progression-weighted mean 20AAR number being higher among proximal tumors compared with distal (Δ_Proximal-Distal_ = 1.1; *P* < 0.001; permutation test; Fig. 4A). This suggests that tumors in the proximal colon have lower WNT activation due to APC loss. We considered other clinical features reported in the 100kGP cohort that could underlie variability of *APC* genotypes but found no significant differences (permutation test, *P* > 0.05) upon stratifying by sex or early onset cancers, defined as <50 years old at resection (Fig. 4B; Supplementary Figs. S9–S11).

Finally, for a subset of colorectal cancers (TCGA, *n* = 509), we stratified tumors by CMS (23). We found no major differences across subtypes, except for a higher progression-weighted mean 20AAR within the CMS3 subtype (associated with KRAS signaling) compared with CMS2 (the canonical WNT subtype; Δ_CMS2-CMS3_ = −0.55; *P* = 0.0048; Supplementary Fig. S12; Supplementary Table S11). A potential interpretation is that colorectal cancers with suboptimal APC inactivation progress via alternative pathways, for example, increased KRAS signaling, in line with recent work suggesting pathway reciprocity between APC–MYC and KRAS pathways (31).

### Secondary WNT drivers can combine with APC inactivation to achieve “just-right” WNT signaling

**Figure 5.**
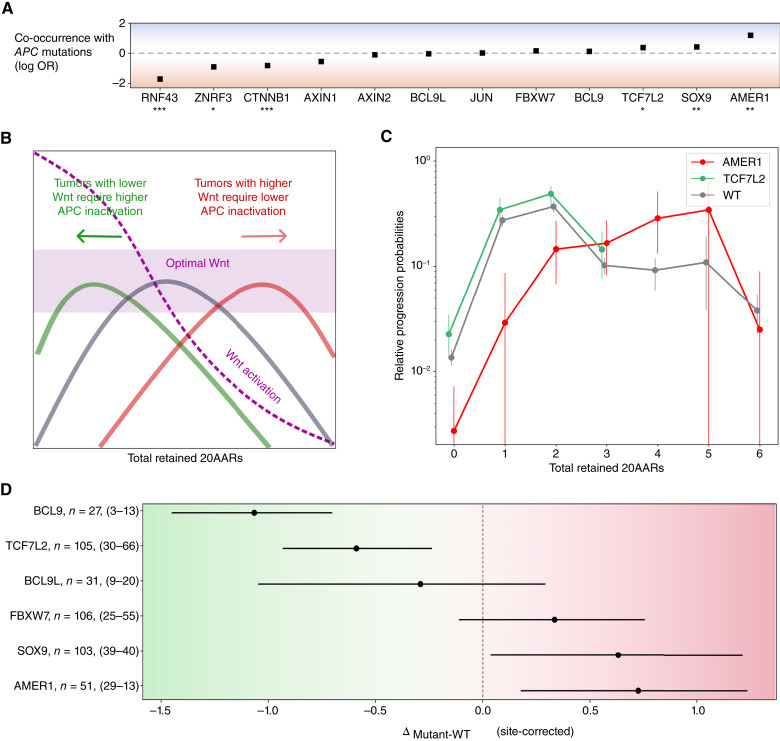
“Just-right” WNT activity at the pathway level. **A,** OR between APC inactivation and pathogenic mutations in other WNT-related genes in the 100kGP MSS cohort (*n* = 1,639; Supplementary Table S12). Fisher test; *, *P* < 0.05; **, *P* < 0.01; ***, *P* < 0.001. **B,** Schematic of the effect of additional mutations in WNT pathway regulators. Assuming that the cancer progression probabilities of *APC*-mutant cells are due to the corresponding level of WNT pathway activation, tumors with WNT-upregulating mutations will require a smaller WNT contribution from *APC* mutations and will thus have relative progression probability curves shifted to the right, and vice versa. **C,** Relative progression probabilities as a function of the total number of retained 20AARs using sequence data of MSS primary colorectal cancers with pathogenic *AMER1* mutations (*n* = 51; red), with *TCF7L2* mutations (*n* = 105; green), and tumors without mutations in non-*APC* WNT regulators (*n* = 825; gray). Whiskers represent 95% CI (bootstrapping); thick dashed vertical lines indicate the progression-weighted mean 20AARs number retained. **D,** Difference in progression-weighted mean 20AARs number for tumors with pathogenic mutations in different WNT genes, Δ_mutant-WT_, corrected by the effect of the anatomic site by taking the weighted average of the difference conditioned on the anatomic site of the tumors. Horizontal bars represent 95% CI (bootstrapping). Numbers next to the gene labels indicate the total number of tumors with mutations in the WNT driver and the number of tumors classified as proximal and distal colon, respectively. We excluded AXIN1, AXIN2, and JUN, as there were not enough samples to perform the site correction (Supplementary Tables S12 and S13). WT, wild-type.

Although *APC* is the main WNT driver in colorectal cancer, other genes also dysregulate the WNT pathway when mutated (32, 33). Thus, we next investigated tumors with additional mutations in WNT drivers to study the “just-right” hypothesis at the pathway level. Primary WNT drivers, such as *RNF43* or *CTNNB1*, have similarly drastic effects as APC inactivation on WNT (34, 35). In the 100kGP cohort, alterations of *RNF43* or *CTNNB1* are found in a minority of sporadic colorectal cancers, mostly microsatellite unstable (MSI), and are mutually exclusive with APC inactivation in MSS tumors (OR = 0.019, *P* = 3.94 × 10^−24^ and OR = 0.15, *P* = 1.46 × 10^−5^, respectively, Fig. 5A; Supplementary Table S12), suggesting an upper bound on WNT activity. Other WNT drivers with smaller effects on WNT activity can co-occur with primary WNT drivers—these are referred to as secondary WNT drivers (36). In 100kGP, driver mutations of *AMER1*, *SOX9*, and *TCF7L2* co-occur with *APC* in MSS tumors (OR = 15.53, *P* = 2.34 × 10^−5^; OR = 2.62, *P* = 7.20 × 10^−4^; and OR = 2.35, *P* = 1.47 · 10^−3^, respectively, Fig. 5A; Supplementary Table S12). These genes have also been found mutated in precancerous lesions (37, 38), emphasizing their tumorigenic effect in combination with canonical WNT drivers. However, how they act in concert with *APC* alterations to attain just-right WNT and even their directional effect—that is, whether they increase or decrease WNT activity—is unclear.

By comparing MSS colorectal cancers with and without secondary WNT driver mutations, we reasoned that the effect of the secondary WNT drivers could be measured under the following rationale. Under the “just-right” model for WNT activity, secondary WNT driver mutations that cause increased WNT are expected to be more frequent in combination with *APC* genotypes that lead to relatively low WNT, that is, those that retain more 20AARs, resulting in a rightward shift in the relative progression probability curve, Δ_mutant-WT_ > 0 (Fig. 5B). Similarly, secondary drivers that cause reduced WNT would be more common with WNT-high *APC* genotypes, which retain fewer 20AARs; hence, a leftward shift in the relative risk curve is expected, with Δ_mutant-WT_ < 0 (Fig. 5B).

In agreement with the theoretical expectation, a clear shift to the right was observed in tumors with driver (loss-of-function) mutations in *AMER1* (Fig. 5C). This shift indicates that *AMER1* mutations tend to occur in tumors with lower-than-average APC inactivation, potentially increasing WNT activity to the “just-right” window, in accordance with both *in vitro* and *in vivo* experiments showing that wild-type AMER1 reduces WNT signaling (39). Conversely, a shift to the left was observed in tumors with driver mutations in *TCF7L2* (Fig. 5C), which all retain 0–3 copies of 20AARs.

To quantitatively classify genes into WNT upregulators or downregulators, we computed Δ_mutant-WT_ weighted by the proportion of mutations occurring in proximal or distal tumors, thus obtaining a metric that is independent of anatomic site-specific biases (Materials and Methods). Using this measure, we predicted mutated *AMER1* and *SOX9* as WNT upregulators (Δ_mutant-WT_ > 0, 95% CI, bootstrapping) and mutations of *TCF7L2* and *BCL9* as WNT downregulators (Δ_mutant-WT_ < 0, 95% CI, bootstrapping) in tumors with APC inactivation, relative to the wild-type protein. Considering that most driver mutations in the genes above result in loss of function (Supplementary Table S13; refs. 3, 28), the findings are consistent with the current understanding of the wild-type protein functions, for example, AMER1 and SOX9 promote APC activity, acting as WNT repressors in healthy tissue, whereas TCF7L2 and BCL9L promote β-catenin transcription (40). Mutations of *FBXW7* and *BCL9L* were consistent with no effect on the cancer progression risk of APC genotypes although this may be due to low sample size and variant-specific functional consequences. For *AXIN1*, *AXIN2*, and *JUN*, site correction was not possible due to the limited number of samples. Seeking orthogonal evidence, we examined the change in AXIN2 expression for the collection of MSS colorectal cancers for which both mutational and RNA sequencing (RNA-seq) data were available (*n* = 89 samples from 100kGP; *n* = 243 samples from TCGA). For the four genes for which our genetic analysis identified significant associations with 20AAR number (AMER1, SOX9, TCF7L2, BCL9), we find the same direction of association in 4 out of 4 cases using TCGA RNA-seq and 3 out of 4 using 100kGP RNA-seq; however, this is without significance due to the small number of tumors with secondary WNT mutations (Supplementary Figs. S7 and S13; Supplementary Tables S8 and S9). A caveat of the expression analysis is that our proposed role for the secondary WNT regulators is at tumor initiation, whereas WNT activity likely varies considerably during cancer progression (38).

### APC inactivation in hypermutant tumors

**Figure 6.**
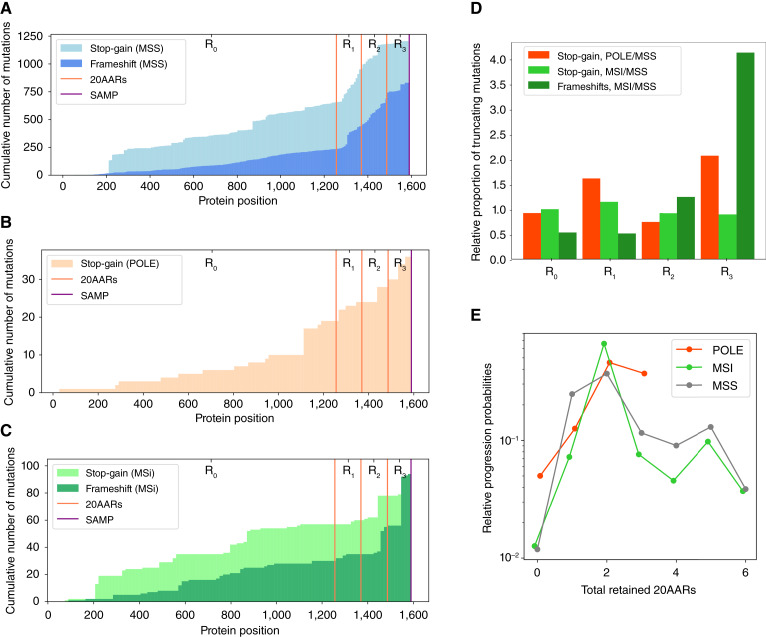
“Just-right” APC inactivation in POLE and MSI colorectal cancers. **A–C,** Cumulative number of stop-gain and frameshift mutations detected per codon position of *APC* in MSS (**A**), *POLE*-mutant (**B**), and MSI (**C**) primary colorectal cancers in the 100kGP cohort. Vertical lines indicate the locations of the 20AAR domains and the SAMP repeat. **D,** Expected proportion of mutations in different regions of *APC* in *POLE*-mutant and MSI relative to MSS, calculated using the mutational signatures detected in healthy colonic crypts (24), *POLE*-mutant crypts (26), and MSI colorectal cancers (Supplementary Tables S4, S5, and S10; ref. 3). **E,** The relative progression probabilities by total number of 20AARs retained in MSS, *POLE*-mutant, and MSI colorectal cancers in the 100kGP cohort.

Thus far, we have focused our analysis on MSS colorectal cancers and excluded hypermutant colorectal cancers, that is, colorectal cancers with mutations affecting the proofreading capability of DNA *POLE*, and microsatellite unstable (MSI) colorectal cancers. These tumors not only have an increased mutational burden but are also characterized by distinct mutational processes (41, 42). Thus, it is not surprising that the landscape of *APC* mutations in *POLE* and MSI colorectal cancers in the 100kGP cohort differs from MSS colorectal cancers (Fig. 6A–C). To assess whether hypermutant colorectal cancers comply with the “just-right” distribution observed for MSS cancers (Fig. 4), we first studied how the intrinsic mutational processes active in hypermutant cancers affect the distribution of *APC* genotypes, assuming that *POLE* mutations and mismatch repair deficiency precede APC inactivation (43).

Integrating data on *POLE* mutational signatures (26) with the sequence context of *APC* (Materials and Methods), we found that *POLE-*mutant–associated signatures result in an expected increased proportion of stop-gain mutations in *APC* regions *R*_*1*_ and *R*_*3*_ compared with healthy crypts (Fig. 6D; Supplementary Table S10). As no frameshifts in *APC* were observed in the *POLE* colorectal cancers in 100kGP (Fig. 6B), we omitted the indel analysis for these tumors. To analyze the distribution of *APC* genotypes in lesions with MSI, we used the genome-wide mutational signatures found in >85% of MSI colorectal cancers in the 100kGP cohort (3), (*n* = 364). Notably, the combined MSI indel signature results in a fivefold bias for frameshifts in region *R*_*3*_ compared with MSS (Fig. 6D; Supplementary Table S10). The consequence of this bias can be observed in Fig. 6C, which displays a sharp increase in the number of frameshift mutations in region *R*_*3*_ and should lead to more retained 20AARs in MSI compared with MSS. As we have shown that proximal lesions are more likely to progress if they retain more 20AARs, the bias could explain, in part, why *APC*-driven MSI lesions tend to occur relatively often in the proximal colon compared with MSS (63:22 and 313:574 proximal/distal ratios in the 100kGP cohort, respectively).

We analyzed the distribution of *APC* mutations in colorectal cancers in 100kGP with pathogenic *POLE* mutations or MSI, mirroring the analysis carried out for MSS colorectal cancers (Materials and Methods). We excluded lesions with copy-number alterations, resulting in *n* = 17 *POLE* samples and *n* = 64 MSI colorectal cancers. Although subtle differences in the relative progression probability curves were observed (Fig. 6E; Supplementary Fig. S14), we again reject the hypothesis that complete APC loss provides maximal colorectal cancer risk, with two copies of 20AARs providing maximal risk in both POLE-deficient and MSI colorectal cancers (two 20AARs in the 95% CI of the mode, bootstrapping). Notably, we found no significant differences in the progression-weighted mean 20AARs number compared with MSS tumors [Δ_MSS-POLE_ = −0.22, 95% CI = (−0.62, 0.15); Δ_MSS-MSI_ = −0.29, 95% CI = (−0.67, 0.08), bootstrapping], whereas without adequately correcting for the characteristic mutational signatures of *POLE* and MSI, the differences were larger and statistically significant in the case of MSI tumors [Δ_MSS-POLE,nc_ = −0.31, 95% CI = (−0.78, 0.2); Δ_MSS-MSI,nc_ = −1.87, 95% CI = (−2.51, −0.81), Supplementary Fig. S14]. This finding emphasizes the importance of mutational bias analysis and suggests that repair deficiencies are indeed acquired prior to APC inactivation.

### “Just-right” APC mutations in FAP

Evidence for the “just-right” hypothesis for *APC* mutations initially came from patients with FAP carrying germline APC mutations*.* Patients with FAP present with large numbers of polyps at early ages and, in most cases, develop colorectal cancer unless treated. In agreement with the “just-right” hypothesis, the somatic hit depends on the germline *APC* mutation, with most lesions retaining 1–3 copies of 20AARs (12, 13). Moreover, patients with FAP show variable polyp burdens and times of cancer onset depending on the specific germline mutation (12, 13).

**Figure 7.**
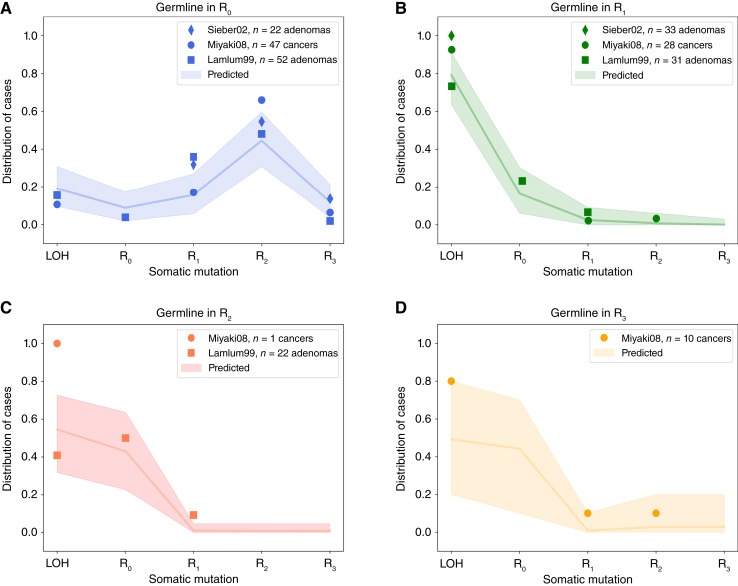
“Just-right” in patients with FAP. **A–D,** The distribution of the *APC* somatic hit in tumors of patients with FAP with germline mutations in different regions. Points indicate the observed distribution in patients with FAP from different studies (11, 12, 22), which collected 93 adenoma, 86 cancer, and 55 adenoma samples from 53, 23, and 18 patients with FAP, respectively. The line indicates the expected distribution calculated using the mutation and cancer progression probabilities estimated from healthy crypts and sporadic colorectal cancer data, with shaded regions for a conservative 95% CI, obtained by performing multinomial simulations with the number of trials given by the maximal number of patients across the studies in each germline group.

We used the progression probabilities inferred from the analysis of sporadic MSS colorectal cancer to assess the concordance between sporadic and FAP tumors. In Fig. 7, the expected distribution of the somatic *APC* hit is compared with the observed distribution in patients with FAP from public datasets reported by Sieber and colleagues (22), Miyaki and colleagues (11), and Lamlum and colleagues (Materials and Methods; ref. 12). For patients with FAP with germline mutations in regions *R*_*1*_, *R*_*2*_, and *R*_*3*_, the FAP data are consistent with the expected distribution, with most adenomas and colorectal cancers developing via LOH or mutations of region *R*_*0*_ (Fig. 7B–D). However, for patients with germline mutations in *R*_*0*_, the sporadic colorectal cancer distribution underestimates the proportion of lesions that develop via mutations in *R*_*2*_, whereas the proportion of tumors with LOH is underestimated in patients with germline mutations in *R*_*1*_ or *R*_*2*_. The discordance might exist for several reasons. A recent study suggests that cooperation between founder clones with different *APC* mutations can achieve “just-right” conditions for tumor development (31); hence, disagreement with the “just-right” model might be due to the polyclonality of some polyps in FAP. We also note unmeasured factors in the FAP data, including the anatomic site of the lesion or mutations in secondary WNT drivers, which both affect optimal *APC* genotypes as discussed above, and that different selective pressures might exist in patients with FAP, further enhancing the selective pressure for 1 to 2 retained 20AARs, for example, due to intercrypt competition. However, the overall concordance between the predicted and observed distribution of the somatic hit suggests similar selection for intermediate APC inactivation in FAP tumors.

## Discussion

Repression of APC function is the canonical tumorigenic event in colorectal cancer, resulting in the WNT pathway dysregulation that is a pervasive feature of this cancer type. The tumor suppressor activity of APC relies crucially on its 20AARs domains, which bind and target β-catenin as an essential part of the β-catenin destruction complex. By integrating somatic and cancer datasets with mathematical modeling, we find a quantitatively consistent signal that biallelic *APC* genotypes, which retain intermediate β-catenin binding activity, confer maximal tumorigenic effect. This signal holds across MSS and hypermutant sporadic colorectal cancers, as well as tumors of patients with FAP. Although a degree of variability in the fitness conferred by specific mutations of oncogenes is expected, for example, G12D variants compared with A146T in *KRAS* (44), that complete loss of the β-catenin–binding 20AARs in tumor-suppressive *APC* does not lead to maximal cancer risk is remarkable.

We find that the majority of variability in *APC* mutations results from context-dependent mutational processes in combination with selection on the total number of 20AARs across both alleles. For MSS colorectal cancers, we found that cells with biallelic *APC* genotypes resulting in two retained 20AARs are at a 50 times higher probability of progressing to colorectal cancer than those in which all binding domains are lost. Although prior experimental work has found that β-catenin binding strengths differ across the 20AAR domains (18, 45), we propose that *APC* mutations primarily influence fitness through the cumulative number of 20AARs. Moreover, the negative correlation that we observed between retained 20AAR number and AXIN2 expression suggests that the *APC* genotype acquired early in tumorigenesis has lasting consequences on WNT levels throughout tumor progression.

Quantitatively strengthening prior observations (21), we found that proximal and distal tumors are under selection for different levels of APC inactivation, with an effect independent of site-specific mutational processes. In particular, proximal tumors retain more 20AARs, suggesting that lower WNT pathway activation is sufficient for progression, agreeing with prior murine studies (30). We further showed that MSI-associated mutational processes favor *APC* alleles with higher 20AAR counts; given the reduced WNT requirements of proximal tumors, this could explain the enrichment of APC-driven MSI tumors in the proximal colon. The selection for different levels of WNT across the colon could be partly explained by the higher baseline expression of WNT genes in the proximal colon (30, 46) and/or might indicate that lesions in the distal colon require higher WNT to progress, for example, due to enhanced immune surveillance (47).

Our WNT pathway–level analysis suggests that secondary WNT drivers contribute to “just-right” by increasing or decreasing the WNT dysregulation induced by *APC* mutations. In particular, we find that pathogenic mutations of *AMER1* are associated with *APC* genotypes that result in lower WNT activation, suggesting that they upregulate WNT, consistent with experimental work (39, 48). Conversely, on aggregate, mutations in *TCF7L2* are associated with *APC* mutations that result in high WNT activation, suggesting that they act to downregulate WNT. Thus, the combined genotype over these WNT drivers fine-tunes the necessary pathway activity, in line with the mini-driver model (49). Although genetic and expression data support the proposed role of the secondary WNT regulators, experimental models combining biallelic APC inactivation and secondary WNT mutations are required to confirm this.

Recent efforts to exploit hyperactivation of cancer pathways for therapeutics (50) underscore the importance of quantifying mutation-specific effects on pathway dysregulation. By integrating somatic and cancer mutation data, we have quantified the relative fitness of “just-right” APC loss. However, the “just-right” effect should not be interpreted entirely deterministically; APC inactivation outside the optimal range can still promote tumorigenesis, albeit at a reduced probability, or via compensatory changes in secondary WNT drivers or other oncogenic pathways (31). The mechanisms underlying why biallelic APC-mutant cells often do not progress to detectable cancers, and the precise stage at which “just-right” WNT signaling favors tumor progression, remain unclear. Comparative analyses of precancerous colorectal tissue should shed light on these questions.

## Supplementary Material

Supplementary Notes Supplementary Notes

Supplementary Table 1 Supplementary Table 1

Supplementary Table 2 Supplementary Table 2

Supplementary Table 3 Supplementary Table 3

Supplementary Table 4 Supplementary Table 4

Supplementary Table 5 Supplementary Table 5

Supplementary Table 6 Supplementary Table 6

Supplementary Table 7 Supplementary Table 7

Supplementary Table 8 Supplementary Table 8

Supplementary Table 9 Supplementary Table 9

Supplementary Table 10 Supplementary Table 10

Supplementary Table 11 Supplementary Table 11

Supplementary Table 12 Supplementary Table 12

Supplementary Table 13 Supplementary Table 13

Supplementary Figure 1 Interdependence between the first and second hit of APC

Supplementary Figure 2 The cumulative distribution of APC mutations and the 15 and 20 AARs.

Supplementary Figure 3 Truncating IDs and SBS on APC occur independently of each other in CRCs

Supplementary Figure 4 Third hits in APC in 100kGP CRCs

Supplementary Figure 5 Progression probabilities of APC genotypes in cBioPortal Cohort

Supplementary Figure 6 The distribution of APC mutations in CRCs.

Supplementary Figure 7 Comparison of total retained 20AAR vs AXIN2 expression

Supplementary Figure 8 Site-specific differences in mutational signatures in the healthy colon.

Supplementary Figure 9 Differences in the progression probabilities by sex or age

Supplementary Figure 10 Age distribution and APC genotypes

Supplementary Figure 11 Arrivals of double APC mutant cells

Supplementary Figure 12 The distribution of CMS subtypes amongst CRCs with different 20AARs.

Supplementary Figure 13 Comparison of classification of secondary Wnt regulators based on retained number 20AARs and expression measures

Supplementary Figure 14 Signature correction in hypermutant CRCs

## Data Availability

For the Genomics England 100kGP cohort, access to full deidentified patient data is restricted to users of the Genomics England Research Environment and is subject to a collaborative agreement that adheres to patient-led governance. For more information about accessing the data, interested readers may contact research-network@genomicsengland.co.uk. Summary tables are provided in the Supplementary Materials and GitHub repository https://github.com/xellbrunet/APC_Analysis_Public. The rest of the analysis was performed on publicly available data. cBioPortal cohort data were obtained from https://bit.ly/447lpyA; cohort data of Christie and colleagues were obtained from ref. 21 (Supplementary Table S1); FAP cohort data were obtained from refs. 11, 12 (Table 1), and ref. 22 (Table 1); mutational signature of healthy crypts data were obtained from ref. 24 (Supplementary Table S2); AXIN2 expression was obtained from cBioPortal (https://bit.ly/45Eo6ZI); and gene expression profiles for CMS classification were obtained from TCGA via the R package TCGAbiolinks. Data files and scripts are available at https://github.com/xellbrunet/APC_Analysis_Public. All other raw data are available from the corresponding author upon request.
