# Supplementary material for: Mathematical Modeling Quantifies “Just-Right” APC Inactivation for Colorectal Cancer Initiation
Source: Cancer Res. 2025 Oct 15;85(24):5113–27. doi: 10.1158/0008-5472.CAN-25-0445 (PMC7618390; doi:10.1158/0008-5472.CAN-25-0445)
Supplement: Supplementary Table 1 [file can-25-0445_supplementary_table_1_suppst1.docx]

## Supplementary Table 1. 100kGP APC CRC cohort

|  | Total | Primary MSS | Primary MSI | Primary POLE |
| --- | --- | --- | --- | --- |
| All | 2023 | 1641 | 364 | 18 |
| APC mutant | 1499 | 1370 | 111 | 18 |
| APC biallelic inactivation | 1118 | 1037 | 64 | 18 |

*Supplementary Table 1.* Number of samples considered from the 100kGP CRC cohort, corresponding to the cohort reported in Cornish *et al*.[[2]](https://paperpile.com/c/CN9ksY/irCCg).
