# Supplementary material for: Mathematical Modeling Quantifies “Just-Right” APC Inactivation for Colorectal Cancer Initiation
Source: Cancer Res. 2025 Oct 15;85(24):5113–27. doi: 10.1158/0008-5472.CAN-25-0445 (PMC7618390; doi:10.1158/0008-5472.CAN-25-0445)
Supplement: Supplementary Table 2 [file can-25-0445_supplementary_table_2_suppst2.docx]

## Supplementary Table 2. APC genotype mapping

| Protein position of clonal frameshift or stop-gain mutations upstream codon 1569, with  i<j<k | | Copy number at APC locus | WGD | Inferred mutant type at initiation | Inferred APC genotype at initiation  M: region of position i  N: region of position j | n | Comments |
| --- | --- | --- | --- | --- | --- | --- | --- |
| i,j |  | [1,1] | False | Bi-allelic mutant | (M,N) | 232 |  |
| i,j | Variants need to be different | [a,b] , b>0 | True | Bi-allelic mutant | (M,N) | 187 |  |
| i |  | [2,0] | False | Copy-neutral LOH | (M, x2) | 104 |  |
| i |  | [1,0] | False | Copy-loss LOH | (M, 0) | 127 |  |
| i |  | [a,0], a>2 | True | Copy-neutral LOH | (M, x2) | 102 |  |
| j |  | [1,0], [2,0] | True | Copy-loss LOH | (M, -) | 150 |  |
| i,j,k | Consider only the two most upstream mutations | [1,1] | False | Bi-allelic mutant | (M,N) | 16 | Three hits |
| i,j | Consider only the most upstream mutation | [2,0] | False | Copy-neutral LOH | (M, x2) | 1 | Three hits |
| i,j | Consider only the most upstream mutation | [1,0] | False | Copy-loss LOH | (M, -) | 5 | Three hits |
| i,j,k | Consider only the two most upstream mutations | [1,1] | True | Bi-allelic mutant | (M,N) | 13 | Three hits |
| i,j | Consider only the most upstream mutation | [2,0] | True | Copy-neutral LOH | (M, x2) | 5 | Three hits |
| i,j | Consider only the most upstream mutation | [1,0] | True | Copy-loss LOH | (M, -) | 16 | Three hits |
| i |  | [a,b] with b>0 | Either | Single mutant | Excluded | 138 |  |
| - |  | unknown or [0,0] |  |  | Excluded | 6 |  |

*Supplementary Table 2.* Classification of *APC* genotypes at initiation. Mapping from *APC* sequence data acquired at tumor sample to *APC* genotype at initiation for all considered combinations of copy number, WGD and annotated variants.
