# Supplementary material for: Mathematical Modeling Quantifies “Just-Right” APC Inactivation for Colorectal Cancer Initiation
Source: Cancer Res. 2025 Oct 15;85(24):5113–27. doi: 10.1158/0008-5472.CAN-25-0445 (PMC7618390; doi:10.1158/0008-5472.CAN-25-0445)
Supplement: Supplementary Table 3 [file can-25-0445_supplementary_table_3_suppst3.docx]

## Supplementary Table 3. cBioPortal datasets

| Study ID | Number of samples |
| --- | --- |
| coad_caseccc_2015 | 7 |
| coad_cptac_2019 | 42 |
| coad_silu_2022 | 123 |
| coadread_dfci_2016 | 105 |
| coadread_genentech | 8 |
| coadread_mskcc | 40 |
| coadread_mskresistance_2022 | 13 |
| coadread_tcga | 96 |
| crc_apc_impact_2020 | 149 |
| crc_dd_2022 | 24 |
| crc_nigerian_2020 | 11 |
| crc_public_genie_bpc | 593 |
| rectal_msk_2019 | 94 |
| Total primary CRC with two truncating mutations on APC | 1,305 |
| Total primary MSS CRC samples with two truncating mutations on APC and no copy number alterations at APC locus | 1,041 |

*Supplementary Table 3.* Public data accessed through cBioPortal [[16,17]](https://paperpile.com/c/CN9ksY/CxwkP+TNLp9) as of 1st of September 2023. Duplicated samples across different studies were removed. Primary tumors with two pathogenic mutations on APC and no copy-number alterations other than WGD were considered. The data and scripts used are available at <https://github.com/xellbrunet/APC_Public>.
