# Supplementary material for: Mathematical Modeling Quantifies “Just-Right” APC Inactivation for Colorectal Cancer Initiation
Source: Cancer Res. 2025 Oct 15;85(24):5113–27. doi: 10.1158/0008-5472.CAN-25-0445 (PMC7618390; doi:10.1158/0008-5472.CAN-25-0445)
Supplement: Supplementary Table 4 [file can-25-0445_supplementary_table_4_suppst4.docx]

## Supplementary Table 4. SBS signatures in MSI CRCs in 100kGP

| Signature | Proportion of samples | Mean exposure | Mean burden |
| --- | --- | --- | --- |
| SBS1 | 0.967 | 0.127 | 12140.95 |
| SBS5 | 0.981 | 0.33 | 32135.3 |
| SBS15 | 0.368 | 0.084 | 10280.01 |
| SBS26 | 0.296 | 0.073 | 9283.43 |
| SBS44 | 0.827 | 0.31 | 34619.09 |
| SBS57 | 0.329 | 0.07 | 8026.49 |

*Supplementary Table 4.* Single-base substitution mutational signatures present in >20% of MSI CRCs in the 100kGP cohort reported in Cornish *et al*.[[2]](https://paperpile.com/c/CN9ksY/irCCg).
