# Supplementary material for: Mathematical Modeling Quantifies “Just-Right” APC Inactivation for Colorectal Cancer Initiation
Source: Cancer Res. 2025 Oct 15;85(24):5113–27. doi: 10.1158/0008-5472.CAN-25-0445 (PMC7618390; doi:10.1158/0008-5472.CAN-25-0445)
Supplement: Supplementary Table 5 [file can-25-0445_supplementary_table_5_suppst5.docx]

## Supplementary Table 5. ID signatures in MSI CRCs in 100kGP

| Signature | Proportion of samples | Mean exposure | Mean burden |
| --- | --- | --- | --- |
| ID1 | 0.989 | 0.134 | 18744.05 |
| ID2 | 1.000 | 0.866 | 123764.01 |

*Supplementary Table 5.* Insertion/deletion mutational signatures present in >20% of MSI CRCs in the 100kGP cohort, determined by 5.
