# Supplementary material for: Mathematical Modeling Quantifies “Just-Right” APC Inactivation for Colorectal Cancer Initiation
Source: Cancer Res. 2025 Oct 15;85(24):5113–27. doi: 10.1158/0008-5472.CAN-25-0445 (PMC7618390; doi:10.1158/0008-5472.CAN-25-0445)
Supplement: Supplementary Table 6 [file can-25-0445_supplementary_table_6_suppst6.docx]

## Supplementary Table 6. Model parameters

| Genotype $\left( M,N \right)$ | Total retained 20AARs | Mutation probability $m_{\left( M,N \right)}$ | Frequency in 100kGP  $f_{\left( M,N \right)}$ | Number in 100kGP | Relative CRC progression probability$\tilde{p}_{\left( M,N \right)}$ | 95 % CI for $\tilde{p}_{\left( M,N \right)}$ | |
| --- | --- | --- | --- | --- | --- | --- | --- |
| (0, 0) | 0 | 0.1733 | 0.0309 | 32 | 0.0036 | 0.0024 | 0.0050 |
| (1, 1) | 2 | 0.0009 | 0.0125 | 13 | 0.1899 | 0.0962 | 0.2868 |
| (2, 2) | 4 | 0.0012 | 0.0039 | 4 | 0.0425 | 0.0000 | 0.0941 |
| (3, 3) | 6 | 0.0014 | 0.0029 | 3 | 0.0373 | 0.0000 | 0.0841 |
| (0, 1) | 1 | 0.0245 | 0.1148 | 119 | 0.0870 | 0.0687 | 0.1070 |
| (1, 2) | 3 | 0.0022 | 0.0039 | 4 | 0.0573 | 0.0170 | 0.1011 |
| (1, 3) | 4 | 0.0023 | 0.0010 | 1 | 0.0158 | 0.0000 | 0.0406 |
| (0, 2) | 2 | 0.0283 | 0.1794 | 186 | 0.1164 | 0.0943 | 0.1386 |
| (2, 3) | 5 | 0.0047 | 0.0039 | 4 | 0.0411 | 0.0136 | 0.0755 |
| (0, 3) | 3 | 0.0314 | 0.0771 | 80 | 0.0531 | 0.0407 | 0.0660 |
| (0,-) | 0 | 0.3318 | 0.0714 | 74 | 0.0037 | 0.0027 | 0.0048 |
| (1,-) | 1 | 0.0244 | 0.0897 | 93 | 0.0654 | 0.0495 | 0.0833 |
| (2,-) | 2 | 0.0281 | 0.1389 | 144 | 0.1010 | 0.0817 | 0.1231 |
| (3,-) | 3 | 0.0301 | 0.0280 | 29 | 0.0168 | 0.0107 | 0.0236 |
| (0, x2) | 0 | 0.2525 | 0.0521 | 54 | 0.0037 | 0.0026 | 0.0048 |
| (1, x2) | 2 | 0.0186 | 0.1331 | 138 | 0.1263 | 0.1016 | 0.1548 |
| (2, x2) | 4 | 0.0214 | 0.0357 | 37 | 0.0289 | 0.0185 | 0.0399 |
| (3, x2) | 6 | 0.0229 | 0.0116 | 12 | 0.0104 | 0.0049 | 0.0163 |

*Supplementary Table 6.* Model parameters for APC-driven CRC initiation in the healthy colon. Mutation probabilities $m_{\left( M,N \right)}$ calculated using the mutational signatures ubiquitous to colonic crypts [[5]](https://paperpile.com/c/CN9ksY/Ikxpy), Methods), frequencies of CRCs $f_{\left( M,N \right)}$ are calculated from the 100kGP cohort of MSS primary CRCs [[2]](https://paperpile.com/c/CN9ksY/irCCg), relative progression probabilities $\tilde{p}_{\left( M,N \right)}$ are calculated using $m_{\left( M,N \right)}$ and $f_{\left( M,N \right)}$ as outlined in Methods. 95% CI obtained by bootstrapping (1,000 iterations).
