# Supplementary material for: Mathematical Modeling Quantifies “Just-Right” APC Inactivation for Colorectal Cancer Initiation
Source: Cancer Res. 2025 Oct 15;85(24):5113–27. doi: 10.1158/0008-5472.CAN-25-0445 (PMC7618390; doi:10.1158/0008-5472.CAN-25-0445)
Supplement: Supplementary Table 7 [file can-25-0445_supplementary_table_7_suppst7.docx]

## Supplementary Table 7. Non-Wnt Canonical drivers

| Cohort | # of 20AARs with maximal progression probability (95% CI) | Progression-weighted mean # 20AARs |
| --- | --- | --- |
| TP53 driver absent (n=275) | 2, [2, 2] | 2.908, [2.305, 3.467] |
| TP53 driver present (n=772) | 2, [2, 2] | 2.104, [1.902, 2.339] |
| KRAS driver absent (n=549) | 1, [1, 3] | 2.777, [2.270, 3.297] |
| KRAS driver present (n=498) | 2, [2, 2] | 2.065, [1.97, 2.169] |
| PIK3CA driver absent (n=848) | 2, [1, 2] | 2.34, [2.036, 2.689] |
| PIK3CA driver present (n=199) | 2, [2, 2] | 2.671, [2.105, 3.342] |
| SMAD4 driver absent (n=961) | 2, [2, 2] | 2.37, [2.048, 2.721] |
| SMAD4 driver present (n=86) | 2, [2, 5] | 2.661, [2.091, 3.174] |

*Supplementary Table 7.* Within MSS CRC samples with biallelic APC inactivation in 100kGP (n=1,047), for cohorts with and without driver mutations on canonical MSS drivers, we report the number of 20AARs with maximal progression probability (corresponding to the mode of the progression probability distribution) and the progression weighted mean number of 20AARs (defined in Methods M2), with the corresponding 95% confidence intervals, obtained by bootstrapping. The canonical drivers were chosen to be those with pathogenic mutations in >9% of MSS CRCs in 100kGP (TP53, KRAS, PIK3CA and SMAD4), where the driver calling was performed by [[2]](https://paperpile.com/c/CN9ksY/irCCg). In all cases, regardless of whether the driver is present or not, we find that 2 20AARs are within the 95% confidence interval for the mode, and the progression weighted mean is between 2 and 3. Hence we reject the “maximal APC loss implies maximal risk” (0 is not within) and the “uniform risk hypothesis”. This suggests that the “just-right” effect is robust to the presence / absence of the aforementioned drivers.
