# Supplementary material for: Mathematical Modeling Quantifies “Just-Right” APC Inactivation for Colorectal Cancer Initiation
Source: Cancer Res. 2025 Oct 15;85(24):5113–27. doi: 10.1158/0008-5472.CAN-25-0445 (PMC7618390; doi:10.1158/0008-5472.CAN-25-0445)
Supplement: Supplementary Table 9 [file can-25-0445_supplementary_table_9_suppst9.docx]

## Supplementary Table 9. AXIN2 mRNA expression in CRCs with Wnt mutations, 100kGP cohort

| **Wnt gene** | **Total retained 20AARs** | **# samples** | **Mean AXIN2 expression (TPM)** | **Weighted difference (Mutant- WT)** |
| --- | --- | --- | --- | --- |
| **WT (only APC)** | 0 | 19 | 423.371 |  |
|  | 1 | 6 | 404.393 |  |
|  | 2 | 21 | 260.954 |  |
|  | 3 | 5 | 212.194 |  |
|  | 4 | 4 | 262.449 |  |
|  | 6 | 1 | 216.994 |  |
| **AMER1** | 0 | 0 | - | 24.447 |
|  | 1 | 0 | - |  |
|  | 2 | 1 | 420.744 |  |
|  | 3 | 0 | - |  |
|  | 4 | 0 | - |  |
|  | 6 | 1 | 106.098 |  |
| **SOX9** | 0 | 3 | 318.1 | 94.309 |
|  | 1 | 2 | 431.6 |  |
|  | 2 | 6 | 425.785 |  |
|  | 3 | 4 | 378.036 |  |
|  | 4 | 0 | - |  |
|  | 6 | 1 | 334.988 |  |
| **FBXW7** | 0 | 2 | 281.299 | 200.758 |
|  | 1 | 1 | 365.811 |  |
|  | 2 | 1 | 1024.405 |  |
|  | 3 | 2 | 594.107 |  |
|  | 4 | 0 | - |  |
|  | 6 | 0 | - |  |
| **BCL9L** | 0 | 1 | 211.365 | 59.06 |
|  | 1 | 0 | - |  |
|  | 2 | 1 | 271.437 |  |
|  | 3 | 1 | 549.776 |  |
|  | 4 | 1 | 362.632 |  |
|  | 6 | 0 | - |  |
| **BCL9** | 0 | 1 | 780.513 | 73.153 |
|  | 1 | 0 | - |  |
|  | 2 | 2 | 192.113 |  |
|  | 3 | 0 | - |  |
|  | 4 | 0 | - |  |
|  | 6 | 0 | - |  |
| **TCF7L2** | 0 | 3 | 398.542 | -25.067 |
|  | 1 | 3 | 187.833 |  |
|  | 2 | 4 | 345.8 |  |
|  | 3 | 1 | 151.687 |  |
|  | 4 | 1 | 406.934 |  |
|  | 6 | 0 | - |  |

*Supplementary Table 9.* Number of samples and mean expression of AXIN2 (TPM) in CRCs with *biallelic APC inactivation* and pathogenic mutations on secondary Wnt regulators (subset of 100kGP), stratified by the total number of 20AARs retained, calculated as detailed in Methods M1. The weighted difference is obtained by weighting the per 20AAR differences (mutant - WT) by their relative frequencies. The colouring of the Wnt gene cells indicates whether the analysis of the genomic data (Figure 5 in the main text) concluded that the secondary Wnt regulator decreases (green) or increases (red) Wnt activity, or was inconclusive (blue). The colouring of the weighted difference cells indicates whether the weighted mean difference is negative (green, meaning mutated samples have lower AXIN2 expression) or positive (red, meaning mutated samples have higher AXIN2 expression).
