# Supplementary material for: Mathematical Modeling Quantifies “Just-Right” APC Inactivation for Colorectal Cancer Initiation
Source: Cancer Res. 2025 Oct 15;85(24):5113–27. doi: 10.1158/0008-5472.CAN-25-0445 (PMC7618390; doi:10.1158/0008-5472.CAN-25-0445)
Supplement: Supplementary Table 10 [file can-25-0445_supplementary_table_10_suppst10.docx]

## Supplementary Table 10. Signature analysis results

|  | Mutation probabilities (stop-gain) | | | | | Mutation probabilities (Frameshifts) | | | |
| --- | --- | --- | --- | --- | --- | --- | --- | --- | --- |
| APC Region | Healthy colon | Healthy right colon | Healthy left colon | POLE-d CRCs | MSI CRCs | Healthy colon | Healthy right colon | Healthy left colon | MSI CRCs |
| *R0* | 0.848 | 0.84758 | 0.84904 | 0.78214 | 0.84827 | 0.73744 | 0.40667 | 0.50181 | 0.50368 |
| *R1* | 0.05476 | 0.05503 | 0.05438 | 0.08898 | 0.06335 | 0.06454 | 0.03586 | 0.04435 | 0.04452 |
| *R2* | 0.05457 | 0.05465 | 0.05444 | 0.04064 | 0.05034 | 0.08539 | 0.03714 | 0.04542 | 0.04558 |
| *R3* | 0.04250 | 0.04274 | 0.04215 | 0.08825 | 0.03804 | 0.11263 | 0.52032 | 0.40843 | 0.40622 |

*Supplementary Table 10.* The relative proportion of stop-gain and frameshifts expected to fall in different regions of *APC*, estimated by considering the ubiquitous mutational signatures found in healthy colon crypts [[5]](https://paperpile.com/c/CN9ksY/Ikxpy), crypts with POLE mutations [[18]](https://paperpile.com/c/CN9ksY/CyO10) and MSI CRCs in the 100kGP cohort (Methods). These are used to estimate the mutation probabilities of different *APC* genotypes.
