# Supplementary material for: Mathematical Modeling Quantifies “Just-Right” APC Inactivation for Colorectal Cancer Initiation
Source: Cancer Res. 2025 Oct 15;85(24):5113–27. doi: 10.1158/0008-5472.CAN-25-0445 (PMC7618390; doi:10.1158/0008-5472.CAN-25-0445)
Supplement: Supplementary Table 11 [file can-25-0445_supplementary_table_11_suppst11.docx]

## Supplementary Table 11. Pairwise comparisons in progression-weighted mean 20AARs across CRCs with different CMS subtypes.

| Cohort | Group 1 | Group 2 | Observed Diff | Raw p-value | Corrected p-value |
| --- | --- | --- | --- | --- | --- |
| TCGA | CMS1 | CMS2 | 0.4658 | 0.0354 | 0.0708 |
|  | CMS1 | CMS3 | -0.0883 | 0.7030 | 0.703 |
|  | CMS1 | CMS4 | 0.3946 | 0.0996 | 0.1494 |
|  | CMS2 | CMS3 | -0.5541 | 0.0008 | 0.0048 |
|  | CMS2 | CMS4 | -0.0712 | 0.6366 | 0.703 |
|  | CMS3 | CMS4 | 0.4829 | 0.0143 | 0.0429 |

*Supplementary Table 11.* Pairwise comparisons in progression-weighted mean 20AARs across CRCs with different CMS subtypes. CMS classification was called for n=509 CRCs in TCGA. p-values corrected for multiple hypothesis testing via Bonferroni correction.
