# Supplementary material for: Mathematical Modeling Quantifies “Just-Right” APC Inactivation for Colorectal Cancer Initiation
Source: Cancer Res. 2025 Oct 15;85(24):5113–27. doi: 10.1158/0008-5472.CAN-25-0445 (PMC7618390; doi:10.1158/0008-5472.CAN-25-0445)
Supplement: Supplementary Table 12 [file can-25-0445_supplementary_table_12_suppst12.docx]

## Supplementary Table 12. Odds ratio between APC and Wnt regulators

| Secondary Wnt | Odds ratio | p-value | Number of samples | | | |
| --- | --- | --- | --- | --- | --- | --- |
|  |  |  | APC only | SecondaryWnt only | Both | Neither |
| RNF43 | 0.019 | 3.938 E-24 | 1375 | 34 | 4 | 226 |
| ZNRF3 | 0.124 | 0.03 | 1377 | 3 | 2 | 257 |
| CTNNB1 | 0.152 | 1.464 E-05 | 1368 | 13 | 11 | 247 |
| AXIN1 | 0.281 | 0.180 | 1376 | 2 | 3 | 258 |
| AXIN2 | 0.788 | 0.590 | 1358 | 5 | 21 | 255 |
| BCL9L | 0.922 | 0.862 | 1325 | 11 | 54 | 249 |
| JUN | 1.037 | 1 | 1368 | 2 | 11 | 258 |
| FBXW7 | 1.441 | 0.155 | 1224 | 21 | 155 | 239 |
| BCL9 | 1.329 | 0.688 | 1337 | 6 | 42 | 254 |
| TCF7L2 | 2.355 | 0.001 | 1216 | 14 | 163 | 246 |
| SOX9 | 2.617 | 0.000720 | 1224 | 12 | 155 | 248 |
| AMER1 | 15.528 | 2.344 E-05 | 1301 | 1 | 78 | 259 |

*Supplementary Table 12*. Odds ratio and number of samples between the number of MSS primary CRCs with APC mutations and mutations in secondary Wnt drivers. P-values determined using Fisher's test. Presence of clonal driver mutations on other Wnt genes (AMER1, AXIN1, AXIN2, BCL9, BCL9L, CTNNB1, FBXW7, JUN, RNF43, SOX9, TCF7L2, ZNRF) as previously determined by Cornish *et al*.[[2]](https://paperpile.com/c/CN9ksY/irCCg).
