# Supplementary material for: Mathematical Modeling Quantifies “Just-Right” APC Inactivation for Colorectal Cancer Initiation
Source: Cancer Res. 2025 Oct 15;85(24):5113–27. doi: 10.1158/0008-5472.CAN-25-0445 (PMC7618390; doi:10.1158/0008-5472.CAN-25-0445)
Supplement: Supplementary Table 13 [file can-25-0445_supplementary_table_13_suppst13.docx]

## Supplementary Table 13. Secondary Wnt variants

| Wnt regulator | Frameshift | Stopgain | Nonsynonymous SNV | Non frameshift insertion/deletion |
| --- | --- | --- | --- | --- |
| AMER1 | 4 | 38 | 3 |  |
| TCF7L2 | 44 | 13 | 35 | 2 |
| SOX9 | 50 | 21 | 10 | 3 |
| BCL9 | 13 |  | 6 | 14 |
| FBXW7 | 5 | 16 | 72 | 1 |
| BCL9L | 10 | 9 | 11 |  |
| JUN | 4 | 2 | 2 |  |
| AXIN1 | 1 |  | 7 |  |
| AXIN2 | 9 | 3 | 10 | 1 |

*Supplementary Table 13.* Number and types of variants included in the analysis of secondary Wnt regulators as previously determined by Cornish *et al*.[[2]](https://paperpile.com/c/CN9ksY/irCCg).
