# Supplementary material for: Mathematical Modeling Quantifies “Just-Right” APC Inactivation for Colorectal Cancer Initiation
Source: Cancer Res. 2025 Oct 15;85(24):5113–27. doi: 10.1158/0008-5472.CAN-25-0445 (PMC7618390; doi:10.1158/0008-5472.CAN-25-0445)
Supplement: Supplementary Figure 1 — Interdependence between the first and second hit of APC [file can-25-0445_supplementary_figure_1_suppsf1.docx]

###### **
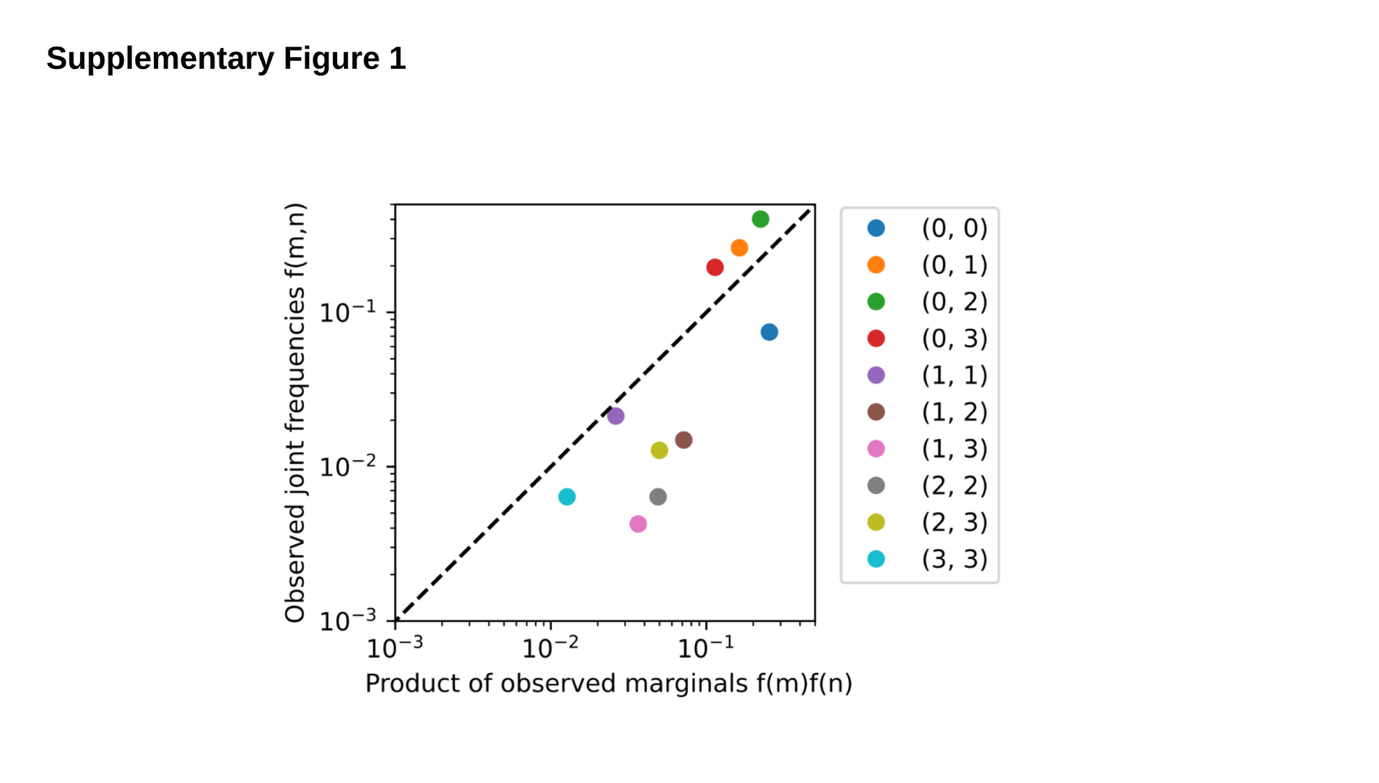
Supplementary Figure 1.** Interdependence between the first and second hit of APC.

The observed distribution of biallelic genotypes in CRCs in 100kGP (n=1,037), against the product of the marginal observed distributions of monoallelic mutations, evidences interdependence between the two mutations (chi=528.31, P< 0.0001). Different colors refer to different biallelic genotypes. Genotype *(M,N)* denotes a truncating mutation in region *R_M_* in one allele and a truncating mutation in region *R_N_* in the other allele. Regions are defined relative to the 20AAR domains, such that a single truncating mutation in region *R_i_* leaves *i* intact 20AAR repeats, where *i* can be 0,1,2 or 3.
