# Supplementary material for: Mathematical Modeling Quantifies “Just-Right” APC Inactivation for Colorectal Cancer Initiation
Source: Cancer Res. 2025 Oct 15;85(24):5113–27. doi: 10.1158/0008-5472.CAN-25-0445 (PMC7618390; doi:10.1158/0008-5472.CAN-25-0445)
Supplement: Supplementary Figure 2 — The cumulative distribution of APC mutations and the 15 and 20 AARs. [file can-25-0445_supplementary_figure_2_suppsf2.docx]

###### **
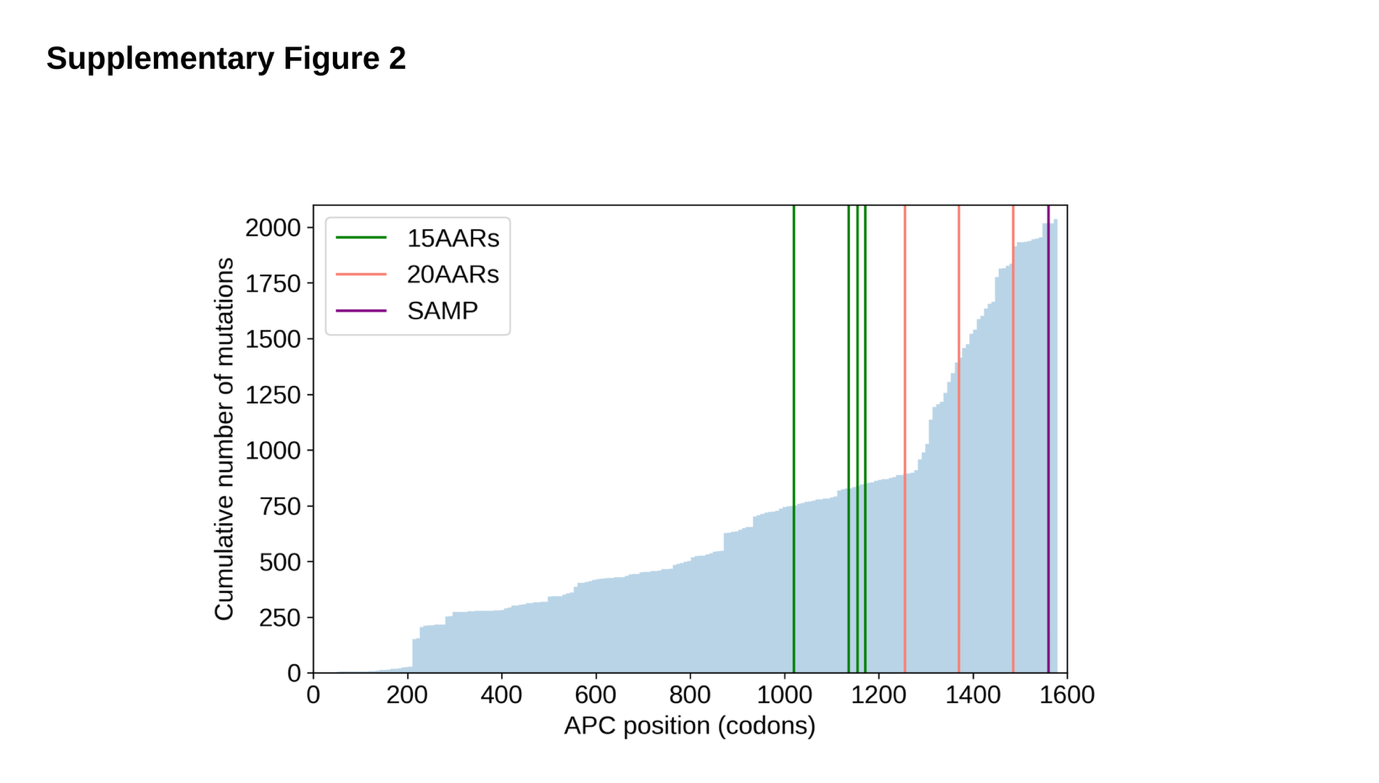
**

###### **Supplementary Figure 2.** The cumulative distribution of APC mutations and the 15 and 20 AARs.

The cumulative distribution of truncating mutations in MSS CRCS in the 100KGP cohort (n=1, with vertical lines indicating the position of the 15AARs (green), the 20AARs (pink) and the SAMP repeat (purple). The increased number of mutations around the 20AARs evidences their relevance in tumorigenesis. Whilst the first 15AAR has been demonstrated to be involved in β-catenin degradation mediated by truncated APC [[1]](https://paperpile.com/c/CN9ksY/9MChM), the slope of the cumulative mutation counts does not increase around the 15AAR, suggesting that there is no strong selection to truncate or retain it. This is further discussed in Supplementary Note 4.
