# Supplementary material for: Mathematical Modeling Quantifies “Just-Right” APC Inactivation for Colorectal Cancer Initiation
Source: Cancer Res. 2025 Oct 15;85(24):5113–27. doi: 10.1158/0008-5472.CAN-25-0445 (PMC7618390; doi:10.1158/0008-5472.CAN-25-0445)
Supplement: Supplementary Figure 3 — Truncating IDs and SBS on APC occur independently of each other in CRCs [file can-25-0445_supplementary_figure_3_suppsf3.docx]

###### **
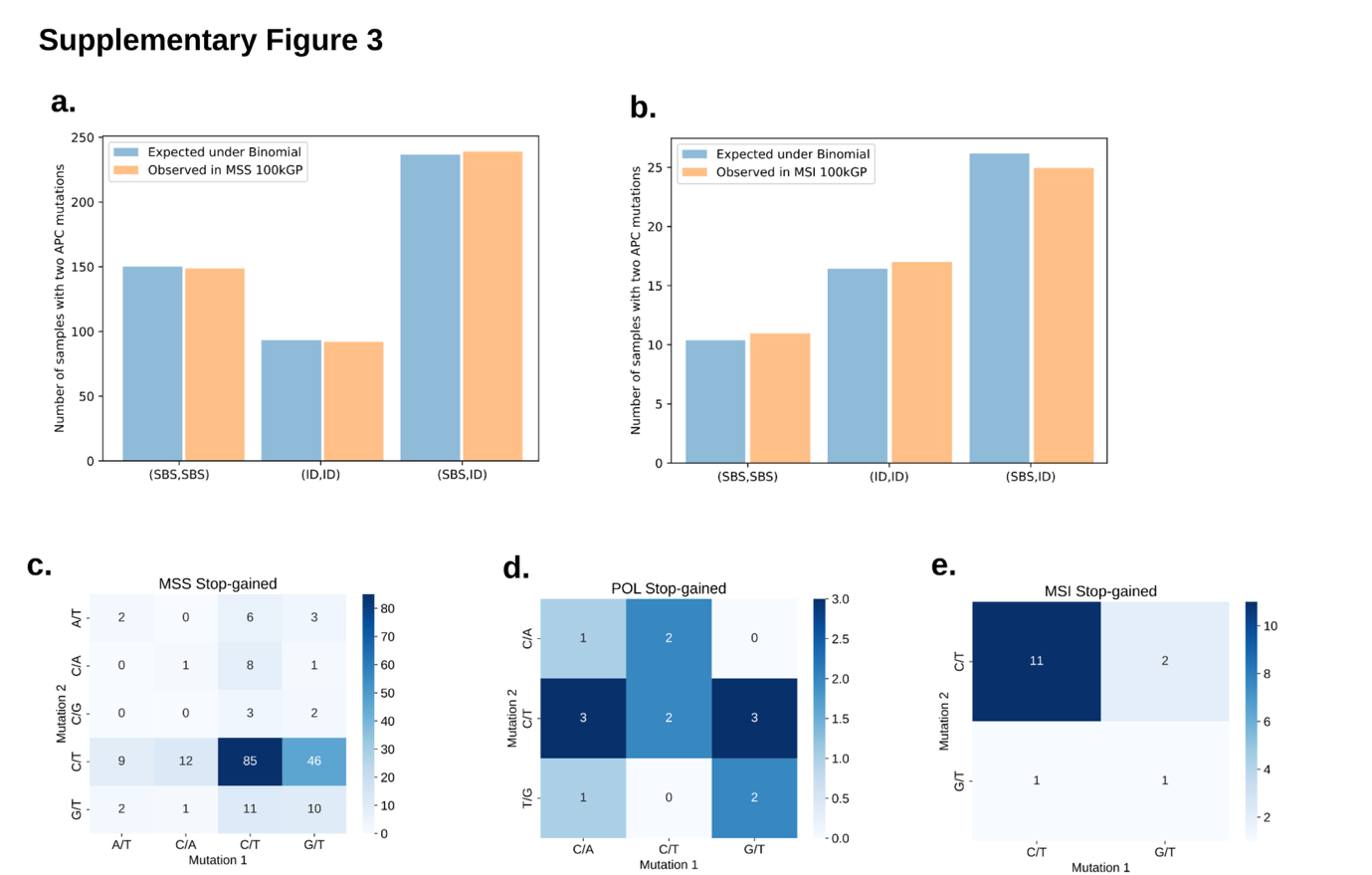
Supplementary Figure 3.** Truncating IDs and SBS on APC occur independently of each other in CRCs. (a-b) The observed number of pairs of APC mutations in MSS and MSI tumors with two SBS, two IDs or one SBS and one ID truncating mutations in 100kGP, is plotted next to the expectation under the assumption of independence between the mutation types, following a Binomial distribution with n=2, p=frequency of SBS. In both cases we fail to reject the null hypothesis of independence (MSS: chi=0.49, P=0.976; MSI: chi=0.104, P=0.949). (c-e) Contingency tables for the numbers of pairs of stop-gain mutation in MSS, POL and MSI tumors in 100kGP, where the coloring of the cell denotes the expected number under independence. In all cases we fail to reject the null hypothesis of independence (MSS: chi=29.9, P=0.187; MSI: chi=0.036, P=0.849; POL: chi=0.036, P=0.3658).
