# Supplementary material for: Mathematical Modeling Quantifies “Just-Right” APC Inactivation for Colorectal Cancer Initiation
Source: Cancer Res. 2025 Oct 15;85(24):5113–27. doi: 10.1158/0008-5472.CAN-25-0445 (PMC7618390; doi:10.1158/0008-5472.CAN-25-0445)
Supplement: Supplementary Figure 4 — Third hits in APC in 100kGP CRCs [file can-25-0445_supplementary_figure_4_suppsf4.docx]

###### **
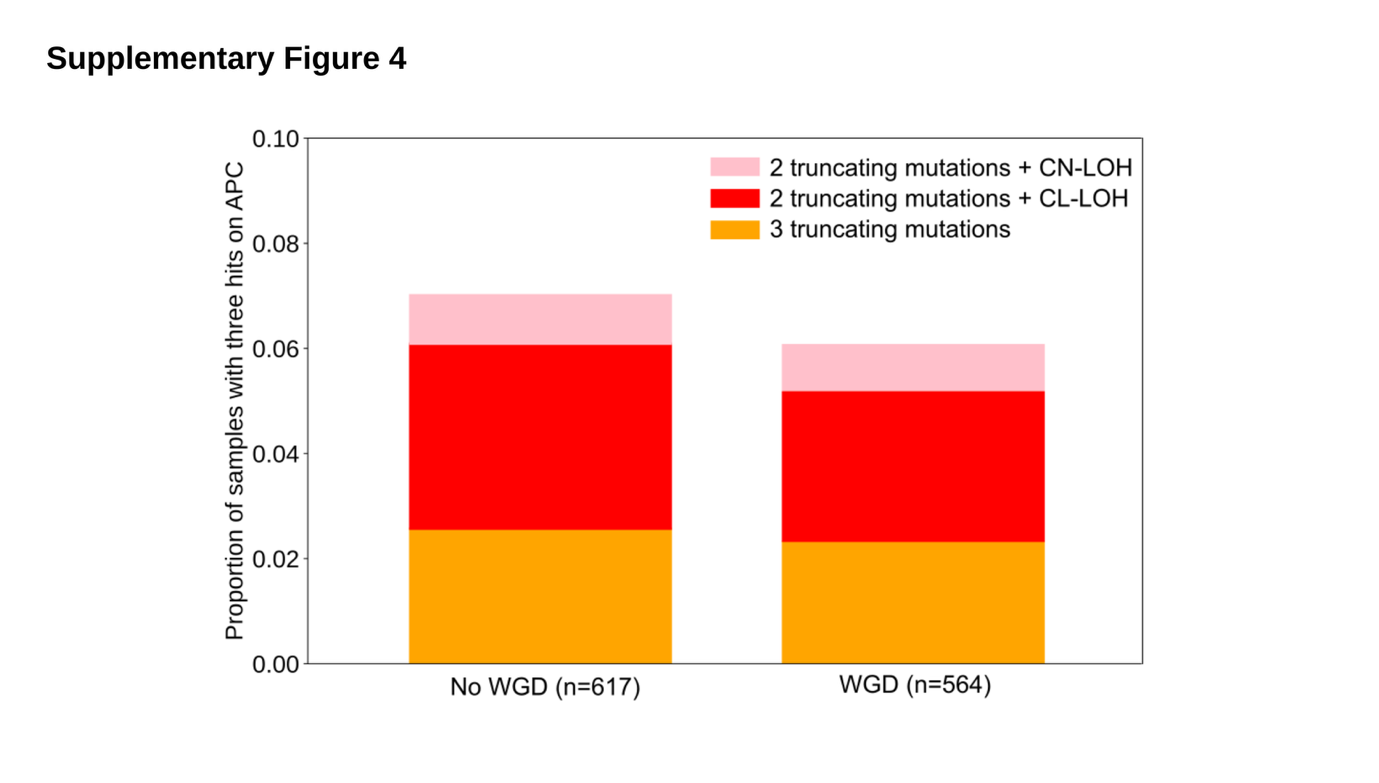
Supplementary Figure 4.** Third hits in APC in 100kGP CRCs.

Proportion of APC-inactivated MSS primary CRCs in the 100kGP cohort (n=1,181) with evidence for third hits of different types, segregated by samples with no whole genome duplication (WGD) (left) and with WGD (right). The overall frequency of MSS CRCs with identifiable third hits in *APC* is 6.2%. The prevalence of third hits is not statistically different in samples with WGD P=0.58, chi2 statistic). WGD status was determined by [[2]](https://paperpile.com/c/CN9ksY/irCCg).
