# Supplementary material for: Mathematical Modeling Quantifies “Just-Right” APC Inactivation for Colorectal Cancer Initiation
Source: Cancer Res. 2025 Oct 15;85(24):5113–27. doi: 10.1158/0008-5472.CAN-25-0445 (PMC7618390; doi:10.1158/0008-5472.CAN-25-0445)
Supplement: Supplementary Figure 5 — Progression probabilities of APC genotypes in cBioPortal Cohort [file can-25-0445_supplementary_figure_5_suppsf5.docx]

###### **
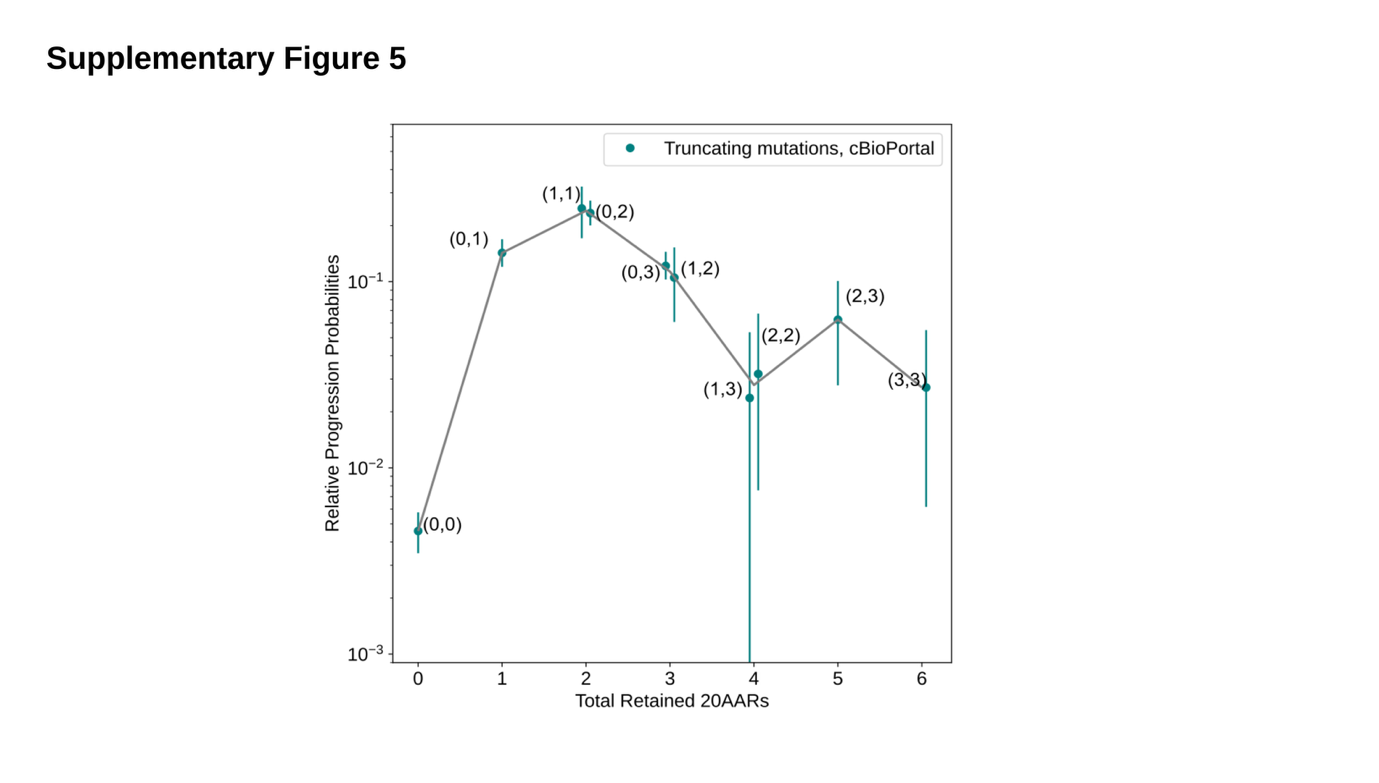
Supplementary Figure 5.** Progression probabilities of APC genotypes in cBioPortal Cohort.

The relative progression probability of different *APC* biallelic mutant genotypes, $\tilde{p}_{(M,N)}$, is plotted against the total number of 20AARs retained across both alleles. The frequencies of genotypes were calculated from sequence data of MSS primary CRCs in the cBioPortal cohort without copy-number alterations on *APC* (n=1,041, Methods). Whiskers for 95% confidence intervals (bootstrapping). The grey line is the weighted average of the progression probability over all genotypes which result in a given number of retained 20AARs. Genotype (*M,N*) denotes a truncating mutation in region *R_M_* in one allele and a truncating mutation in region *R_N_* in the other allele. Regions are defined relative to the 20AAR domains, such that a single truncating mutation in region *R_i_* leaves *i* intact 20AAR repeats, where *i* can be 0,1,2 or 3. The total number of 20AARs is given by *M*+*N*.
