# Supplementary material for: Mathematical Modeling Quantifies “Just-Right” APC Inactivation for Colorectal Cancer Initiation
Source: Cancer Res. 2025 Oct 15;85(24):5113–27. doi: 10.1158/0008-5472.CAN-25-0445 (PMC7618390; doi:10.1158/0008-5472.CAN-25-0445)
Supplement: Supplementary Figure 6 — The distribution of APC mutations in CRCs. [file can-25-0445_supplementary_figure_6_suppsf6.docx]

###### **
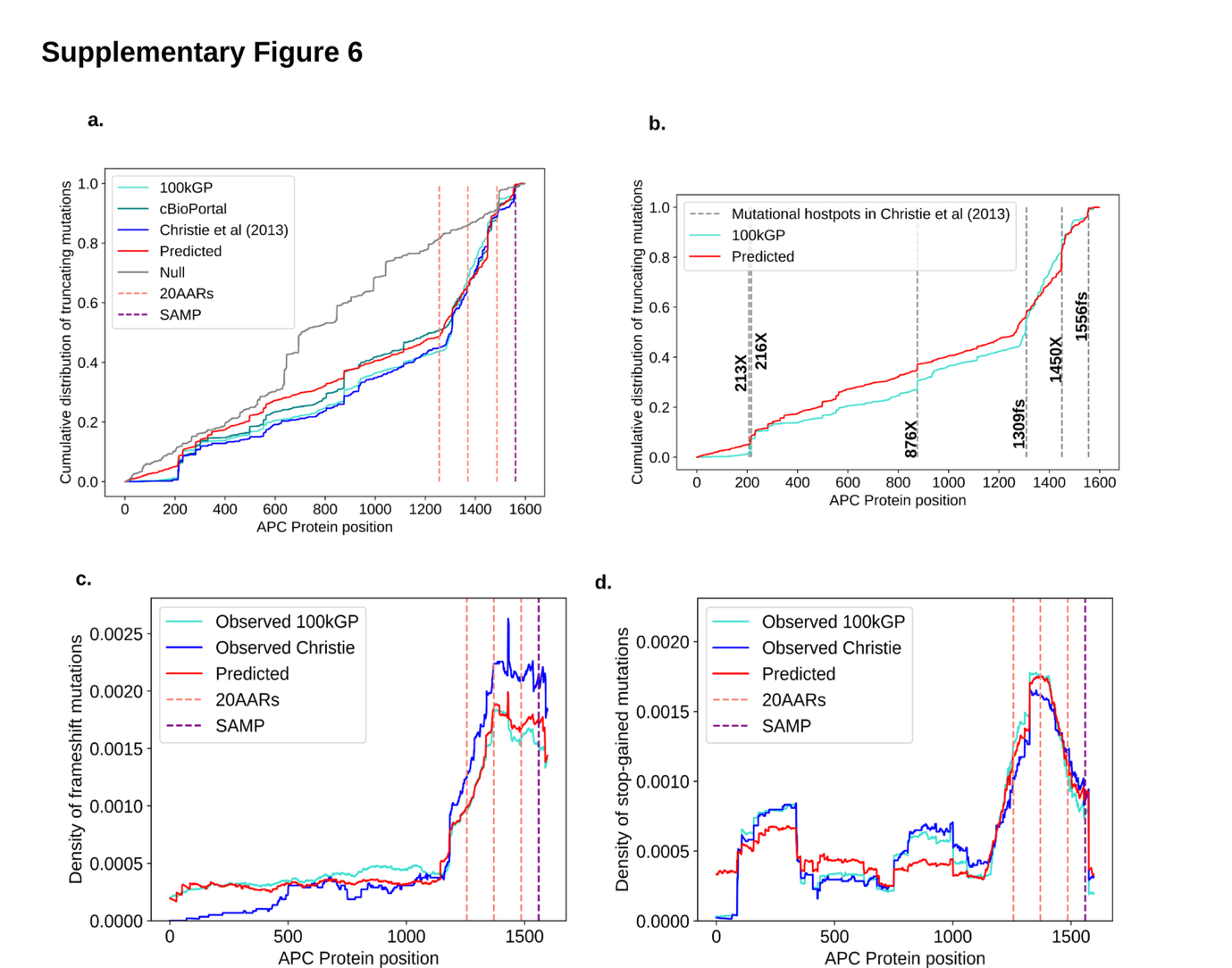
Supplementary Figure 6.** The distribution of APC mutations in CRCs.

(a) The cumulative distribution of truncating (stop gained and frameshift) mutations in *APC*, as observed in MSS CRC cohorts (cyan for 100kGP n=1,037, green for cBioPortal n=1,041 and dark blue for [[3]](https://paperpile.com/c/CN9ksY/8OlEm) n=630), predicted by the model with selection on the total 20AARs (red) and the null ‘Uniform risk’ model (grey). The null is calculated by simulating mutations occurring on *APC* according to the probability distribution calculated using the mutational processes active in the healthy colon. The predicted is calculated by multiplying the null by the progression probability of the number of retained 20AARs. (b) The cumulative distribution of truncating (stop gained and frameshift) mutations in *APC* observed in MSS CRCs in 100kGP and predicted by our model, with vertical lines indicating the mutational hotspots reported by Christie *et al* [*[2]*](https://paperpile.com/c/CN9ksY/irCCg), showing that our predictions and data recover the positions of mutational hotspots (corresponding to jumps in the cumulative distribution). (c-d) The density of frameshift and stop gained mutations of *APC*, as observed in CRC cohorts and predicted by our model, calculated as the rolling average from the cumulative distribution (window size 100 nucleotides). For frameshift mutations, the prediction and the distribution in 100kGP are in close agreement, and both differ from the distribution reported by Christie *et al*, likely due to advances in the resolution of indel calling. Most discrepancies are within the distribution of stop-gain mutations upstream of the first 20AAR. A clear difference is the absence of stop-gain mutations in the data upstream codon 200, likely due to translation re-initiation at a downstream AUG (e.g. in codons 183, 199 or 209), preventing stop-gain mutations in the N terminus driving cancer [[4]](https://paperpile.com/c/CN9ksY/OirYg). The predicted distribution also underestimates the number of stop-gain mutations in positions 216 and 876 (CGA>TGA) that could be driven by methylation, which is not accounted for in our model.
