# Supplementary material for: Mathematical Modeling Quantifies “Just-Right” APC Inactivation for Colorectal Cancer Initiation
Source: Cancer Res. 2025 Oct 15;85(24):5113–27. doi: 10.1158/0008-5472.CAN-25-0445 (PMC7618390; doi:10.1158/0008-5472.CAN-25-0445)
Supplement: Supplementary Figure 7 — Comparison of total retained 20AAR vs AXIN2 expression [file can-25-0445_supplementary_figure_7_suppsf7.docx]

###### **
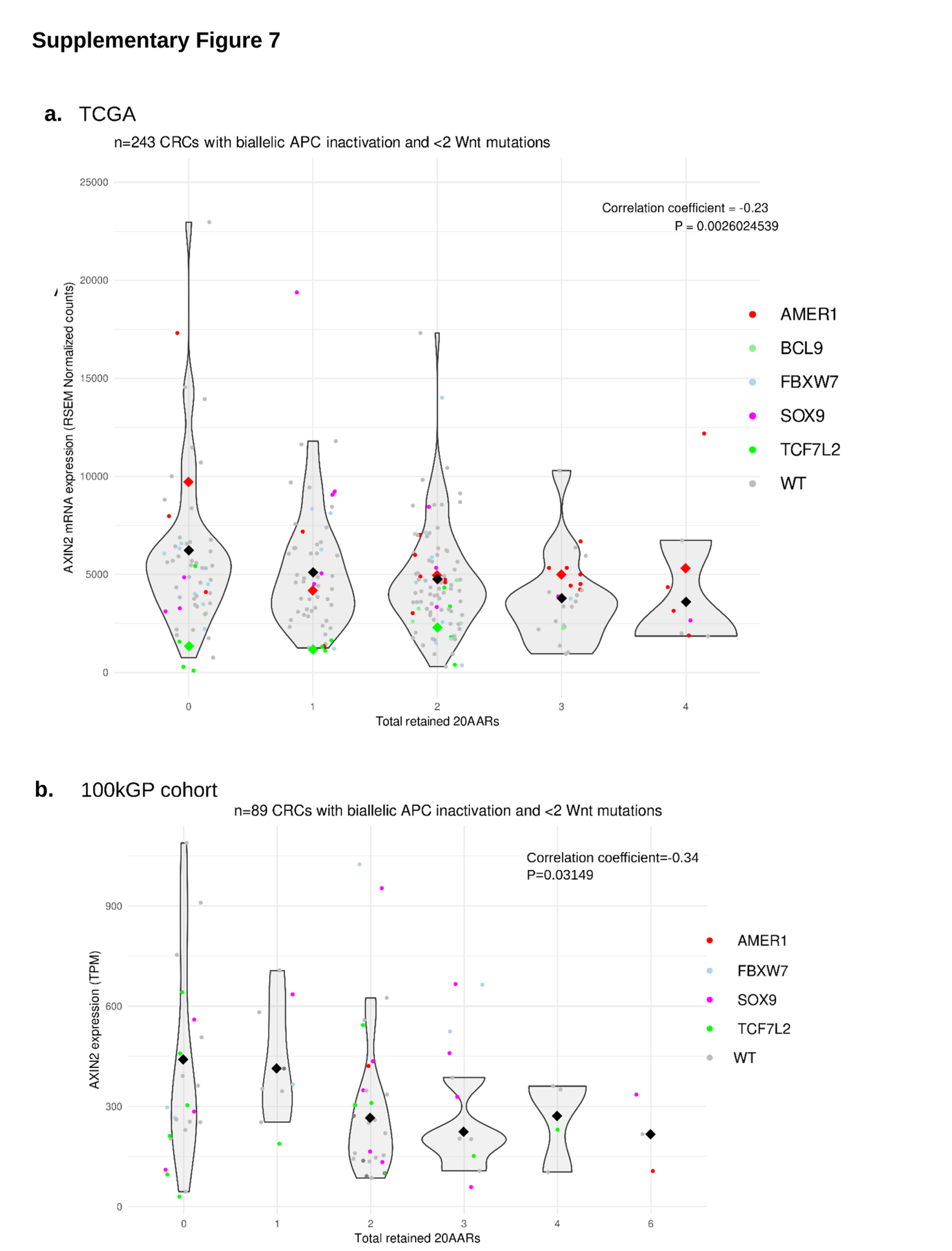
Supplementary Figure 7.** Comparison of total retained 20AAR vs AXIN2 expression.

(a) AXIN2 RNA expression (RSEM normalised counts) in CRCs with biallelic APC inactivation and mutations on secondary Wnt regulator from TCGA cohort and (b) AXIN2 RNA expression (TPM) for a subset of CRCs in the 100kGP cohort with biallelic APC inactivation and mutations on secondary Wnt regulators. Violin plots of the RNA expression of AXIN2 in CRCs with APC inactivation and no other mutations on Wnt regulators reported in CBioPortal, stratified by the total number of 20AARs retained, calculated as detailed in Methods M1. Coloured dots indicate the AXIN2 expression of tumours with driver mutations on a secondary Wnt regulator (red=AMER1, pink=SOX9, blue=FBXW7, green=TCF7L2, light green=BCL9). Grey dots indicate the AXIN2 expression of tumours with APC inactivation but no additional driver mutations on Wnt regulators. These are used to calculate the Pearson correlation between AXIN2 expression and total retained 20AARs (cBioPortal: n=168, cor=-0.23, P=2.6*10^-3^; 100kGP: n= 56, cor=-0.34, P=3.1*10^-2^). The diamond-shaped black dots indicate the mean of the samples with only APC inactivation.
