# Supplementary material for: Mathematical Modeling Quantifies “Just-Right” APC Inactivation for Colorectal Cancer Initiation
Source: Cancer Res. 2025 Oct 15;85(24):5113–27. doi: 10.1158/0008-5472.CAN-25-0445 (PMC7618390; doi:10.1158/0008-5472.CAN-25-0445)
Supplement: Supplementary Figure 8 — Site-specific differences in mutational signatures in the healthy colon. [file can-25-0445_supplementary_figure_8_suppsf8.docx]

###### **
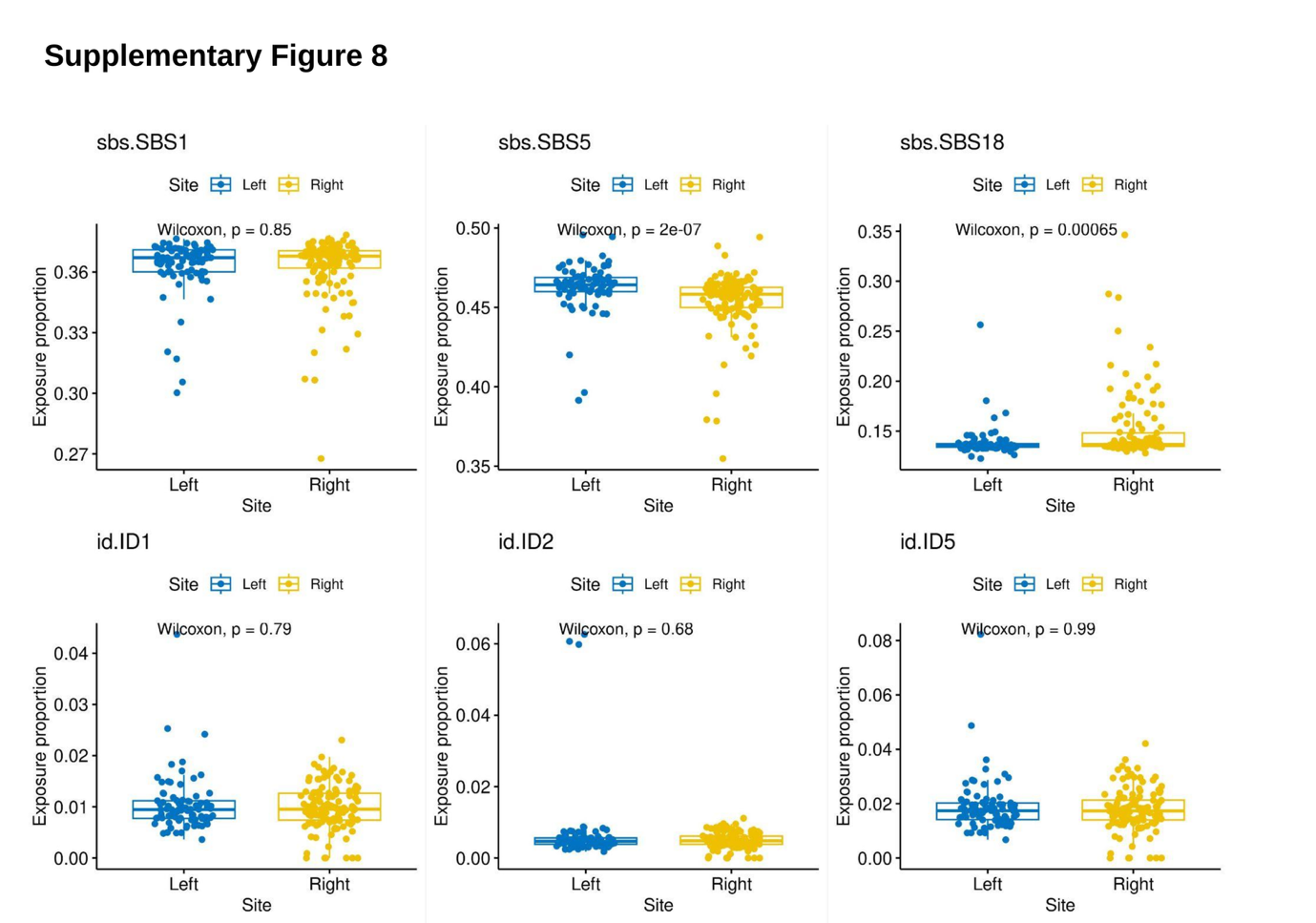
Supplementary Figure 8.** Site-specific differences in mutational signatures in the healthy colon.

For mutational signatures observed ubiquitously in the healthy colon, we show the proportion of signature exposures, calculated by Lee-Six *et al* [[5]](https://paperpile.com/c/CN9ksY/Ikxpy) for healthy colonic crypts labelled as Left colon (corresponding to distal) or Right colon (corresponding to proximal). Significant site-specific differences exist for SBS5 and SBS18, although the magnitude of the effect is relatively minor and so is unlikely to largely contribute to site-specificity of *APC* genotypes in CRCs.
