# Supplementary material for: Mathematical Modeling Quantifies “Just-Right” APC Inactivation for Colorectal Cancer Initiation
Source: Cancer Res. 2025 Oct 15;85(24):5113–27. doi: 10.1158/0008-5472.CAN-25-0445 (PMC7618390; doi:10.1158/0008-5472.CAN-25-0445)
Supplement: Supplementary Figure 9 — Differences in the progression probabilities by sex or age [file can-25-0445_supplementary_figure_9_suppsf9.docx]

###### **
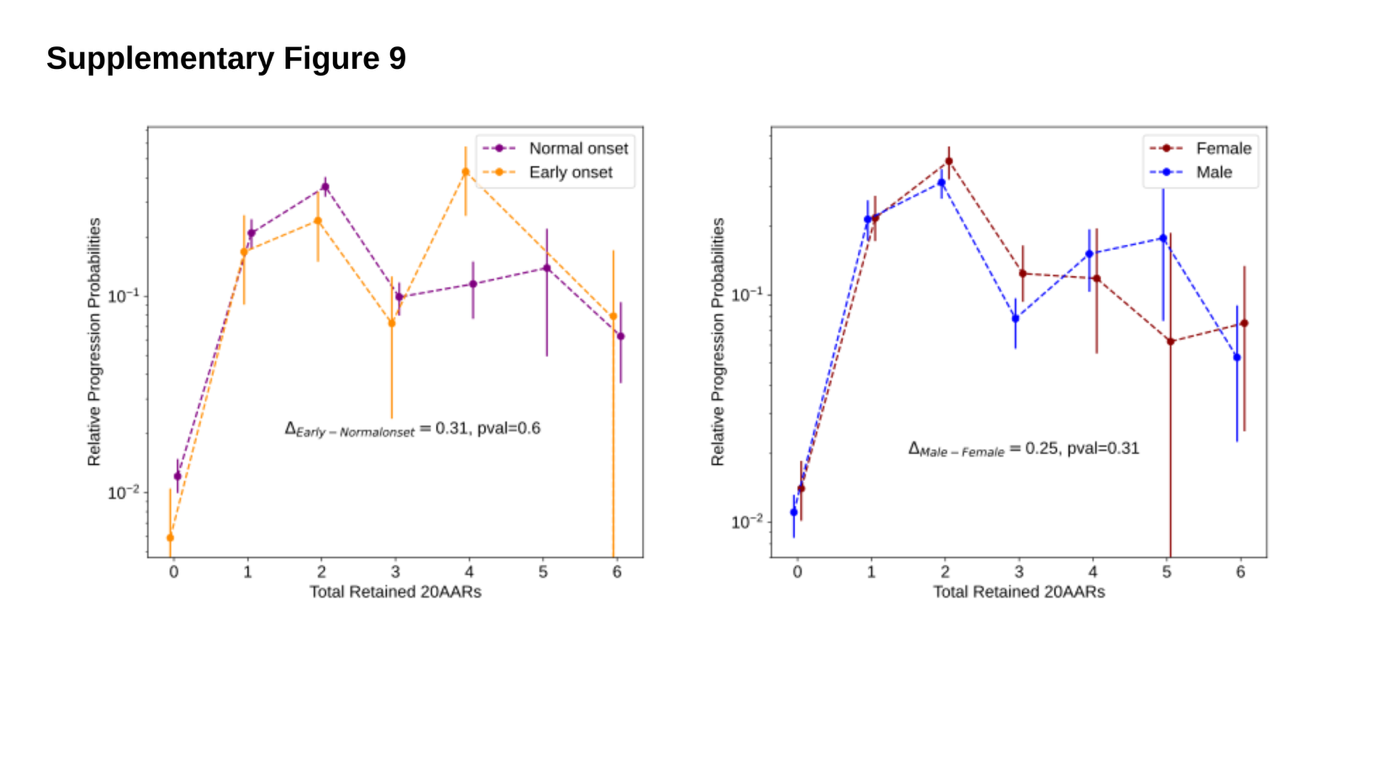
Supplementary Figure 9.** Differences in the progression probabilities by sex or age.

The relative progression probability versus total number of 20AARs retained over both alleles, for tumours in male (orange) versus female (purple) patients, and in patients with early onset (<50 years old at resection, purple) versus normal onset (>50 years old at resection, yellow). Whiskers on points indicate 95% confidence intervals (bootstrapping). The difference in the progression-weighted mean 20AARs number is indicated by Δ (Methods)_._ We find no statistically significant differences between tumours in male versus female patients (P=0.33, permutation test) nor in patients with early onset versus normal onset (P=0.73, permutation test).
