# Supplementary material for: Mathematical Modeling Quantifies “Just-Right” APC Inactivation for Colorectal Cancer Initiation
Source: Cancer Res. 2025 Oct 15;85(24):5113–27. doi: 10.1158/0008-5472.CAN-25-0445 (PMC7618390; doi:10.1158/0008-5472.CAN-25-0445)
Supplement: Supplementary Figure 10 — Age distribution and APC genotypes [file can-25-0445_supplementary_figure_10_suppsf10.docx]

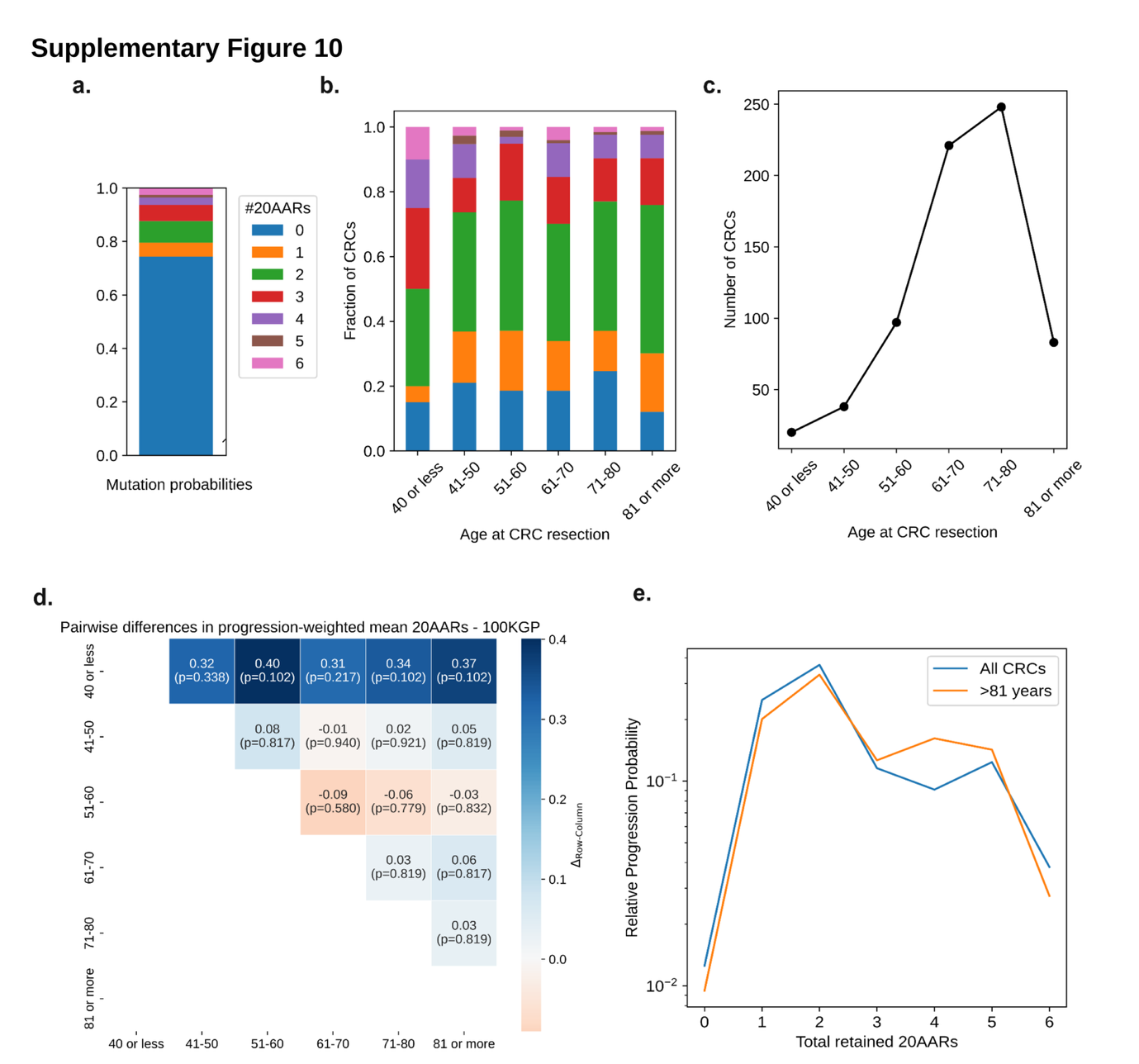


###### **Supplementary Figure 10.** Age distribution and APC genotypes.

(a) The mutation probabilities of double mutants with different numbers of 20AARs. (b) The fraction of CRCs in 100kGP with different numbers of 20AARs for different ranges of patient age at resection of the tumor. (c) The total number of CRCs in 100kGP for different ranges of patient age at resection of the tumor. (d) Pairwise differences in progression-weighted mean 20AAR number across patient groups with different ages calculated from the MSS cohort of CRCs in 100kG. P values indicated in the corresponding cells, obtained via permutation tests, corrected for multiple hypothesis testing (Bonferroni correction). (e) The relative progression probabilities calculated from the MSS cohort of CRCs in 100kGP (blue) and for the subset of patients >81 (orange).
