# Supplementary material for: Mathematical Modeling Quantifies “Just-Right” APC Inactivation for Colorectal Cancer Initiation
Source: Cancer Res. 2025 Oct 15;85(24):5113–27. doi: 10.1158/0008-5472.CAN-25-0445 (PMC7618390; doi:10.1158/0008-5472.CAN-25-0445)
Supplement: Supplementary Figure 11 — Arrivals of double APC mutant cells [file can-25-0445_supplementary_figure_11_suppsf11.docx]

###### **
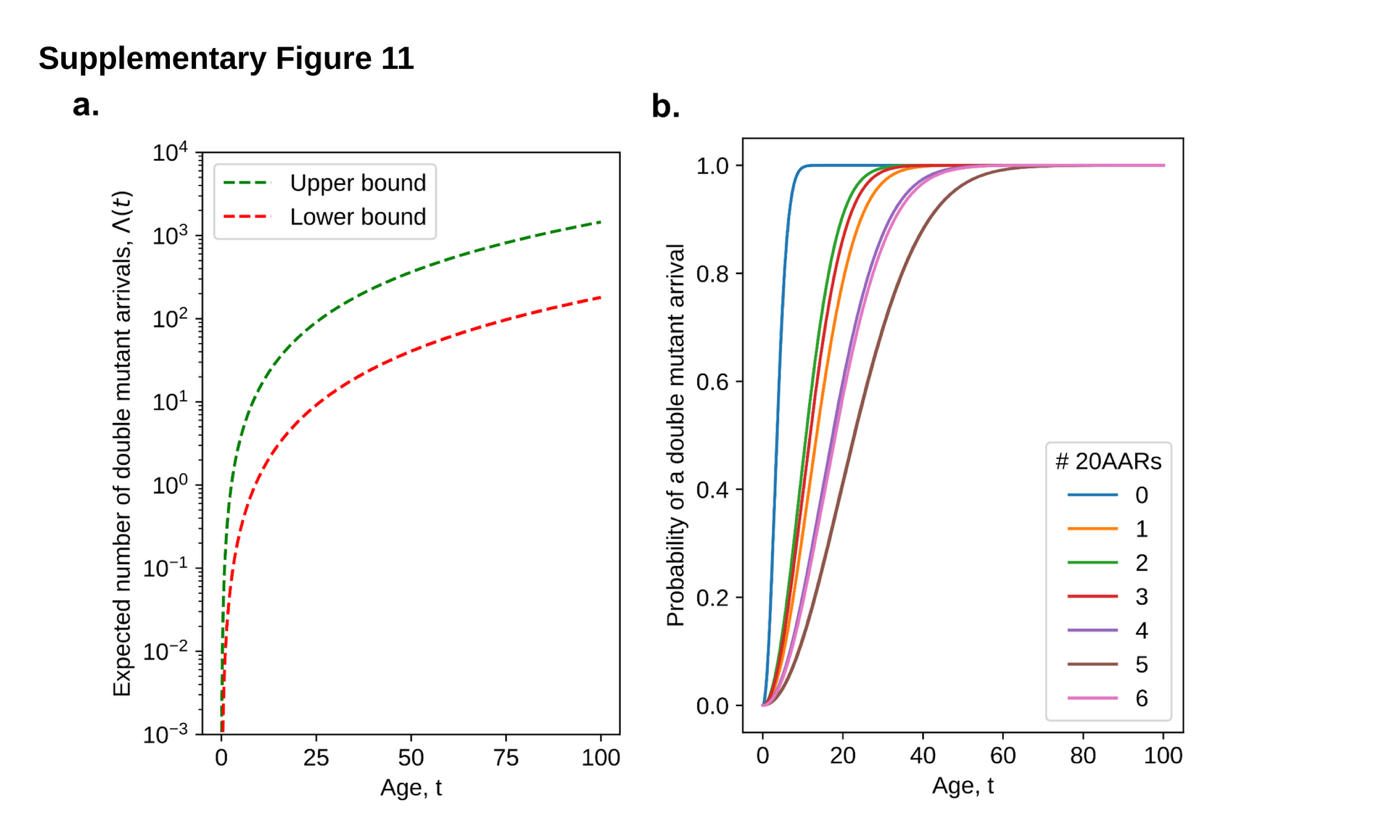
Supplementary Figure 11.** Arrivals of double APC mutant cells.

(a) The expected number of arrivals by a given age, $\Lambda(t)$, where the lower bound takes$n_{s}=5$ stem cells per colonic crypt and $N=10^{7}$ total crypts and the upper bound $n_{s}=7$ and $N=10^{8}$. (b) The probability of at least one arrival of a double mutant with different number of 20AARs by age t, $1-e^{-m_{X} \Lambda(t)}$, where $\Lambda(t)$ is given by Equation S3 in Supplementary Note 3 with $n_{s}=6$ and $N=5\cdot10^{7}$, and $m_{X}$ is the probability of a total retaining $X$ 20AARs.
