# Supplementary material for: Mathematical Modeling Quantifies “Just-Right” APC Inactivation for Colorectal Cancer Initiation
Source: Cancer Res. 2025 Oct 15;85(24):5113–27. doi: 10.1158/0008-5472.CAN-25-0445 (PMC7618390; doi:10.1158/0008-5472.CAN-25-0445)
Supplement: Supplementary Figure 12 — The distribution of CMS subtypes amongst CRCs with different 20AARs. [file can-25-0445_supplementary_figure_12_suppsf12.docx]

###### **
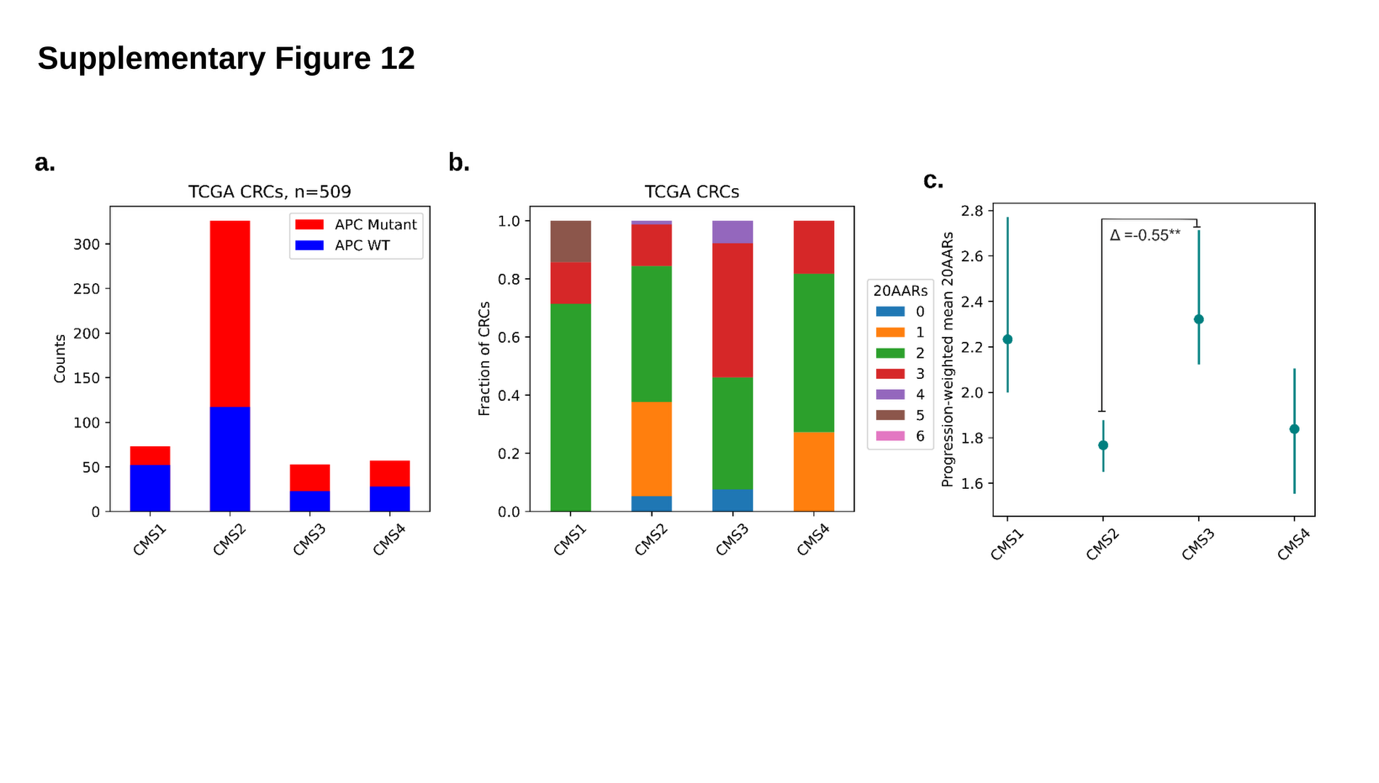
Supplementary Figure 12**. The distribution of CMS subtypes amongst CRCs with different 20AARs.

(a) The number of CRCs per CMS subtype in the subset of TCGA for which RNA seq and genomic data was available (n=509), with blue denoting the number of CRCs with no oncogenic *APC* mutations, and red denoting those with oncogenic *APC* mutations. CMS classification was called using R package CMSclassifier [[6]](https://paperpile.com/c/CN9ksY/R2ye) (Methods M1.4). (b) For CRCs with APC inactivation, the fraction of different numbers of retained 20AARs per CMS subtype. (c) Progression-weighted mean 20AARs of CRCs in both cohorts stratified by CMS subtype, with vertical lines denoting the 95% confidence intervals. The difference between the progression-weighted means of CMS2 and CMS3 in TCGA CRCs was found to be statistically significant (P=0.0048).
