# Supplementary material for: Mathematical Modeling Quantifies “Just-Right” APC Inactivation for Colorectal Cancer Initiation
Source: Cancer Res. 2025 Oct 15;85(24):5113–27. doi: 10.1158/0008-5472.CAN-25-0445 (PMC7618390; doi:10.1158/0008-5472.CAN-25-0445)
Supplement: Supplementary Figure 13 — Comparison of classification of secondary Wnt regulators based on retained number 20AARs and expression measures [file can-25-0445_supplementary_figure_13_suppsf13.docx]

###### **
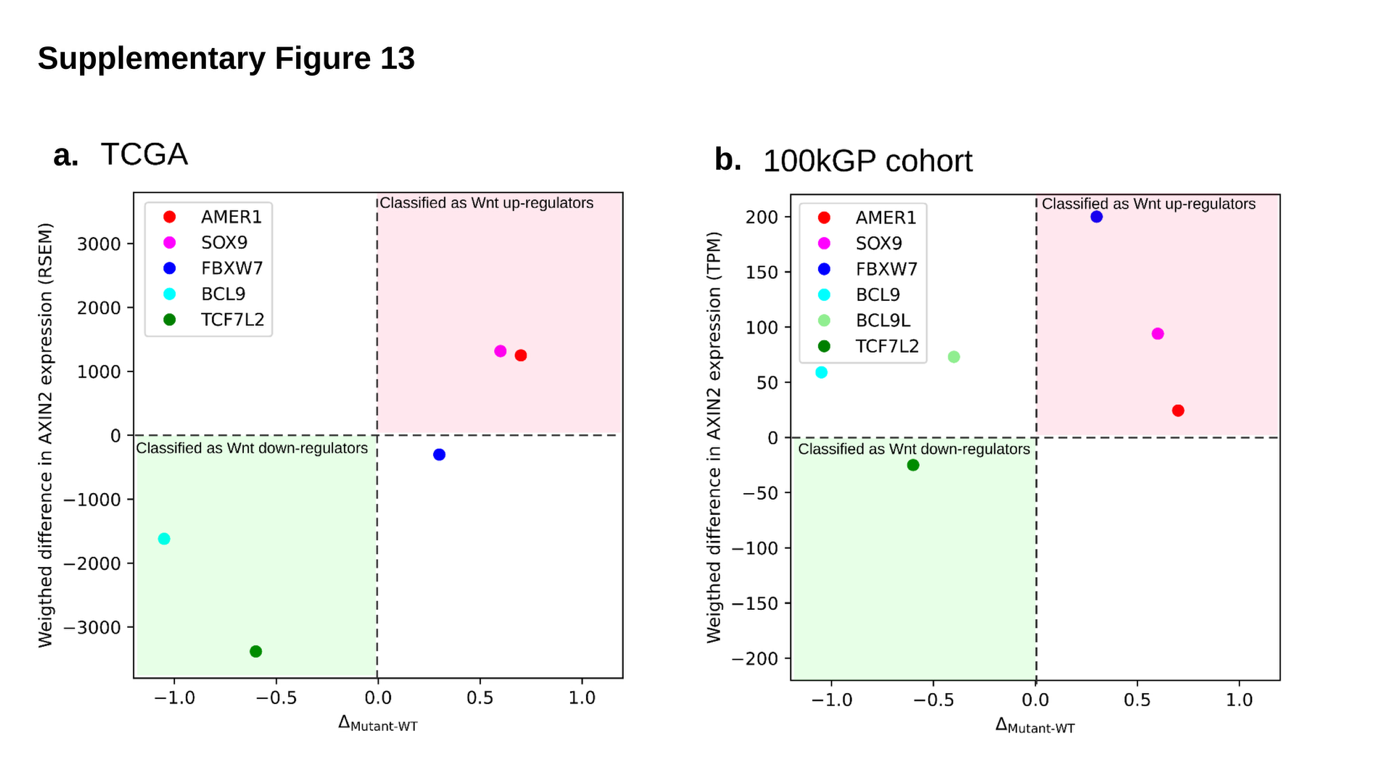
Supplementary Figure 13.** Comparison of classification of secondary Wnt regulators based on retained number 20AARs and expression measures.

The progression-weighted difference in mean 20AARs calculated from bulk sequence data of tumours in the 100kGP cohort (Figure 5d) against (a) the weighted difference in mean AXIN2 RNA expression (RSEM normalised counts, Mutant- WT) in CRCs with biallelic APC inactivation and mutations on secondary Wnt regulator from TCGA cohort (n=243) and (b) the weighted difference in mean AXIN2 RNA expression (TPM) for a subset of CRCs in the 100kGP cohort with biallelic APC inactivation and mutations on secondary Wnt regulators (n=89). The weighted difference in AXIN2 expression is obtained by weighting the per 20AAR differences (mutant - WT) by their relative frequencies (Supplementary Tables 11-12). For AMER1 and SOX9, the genetic data and the two cohorts of RNA sequence data are in concordance with an increased level of Wnt regulation in tumors with driver mutations (quadrant colored in red). For TCF7L2, the genetic data and the two cohorts of RNA sequence data are in concordance with a reduced level of Wnt regulation in tumors with driver mutations (quadrant colored in green).
