# Supplementary material for: Mathematical Modeling Quantifies “Just-Right” APC Inactivation for Colorectal Cancer Initiation
Source: Cancer Res. 2025 Oct 15;85(24):5113–27. doi: 10.1158/0008-5472.CAN-25-0445 (PMC7618390; doi:10.1158/0008-5472.CAN-25-0445)
Supplement: Supplementary Figure 14 — Signature correction in hypermutant CRCs [file can-25-0445_supplementary_figure_14_suppsf14.docx]

###### **
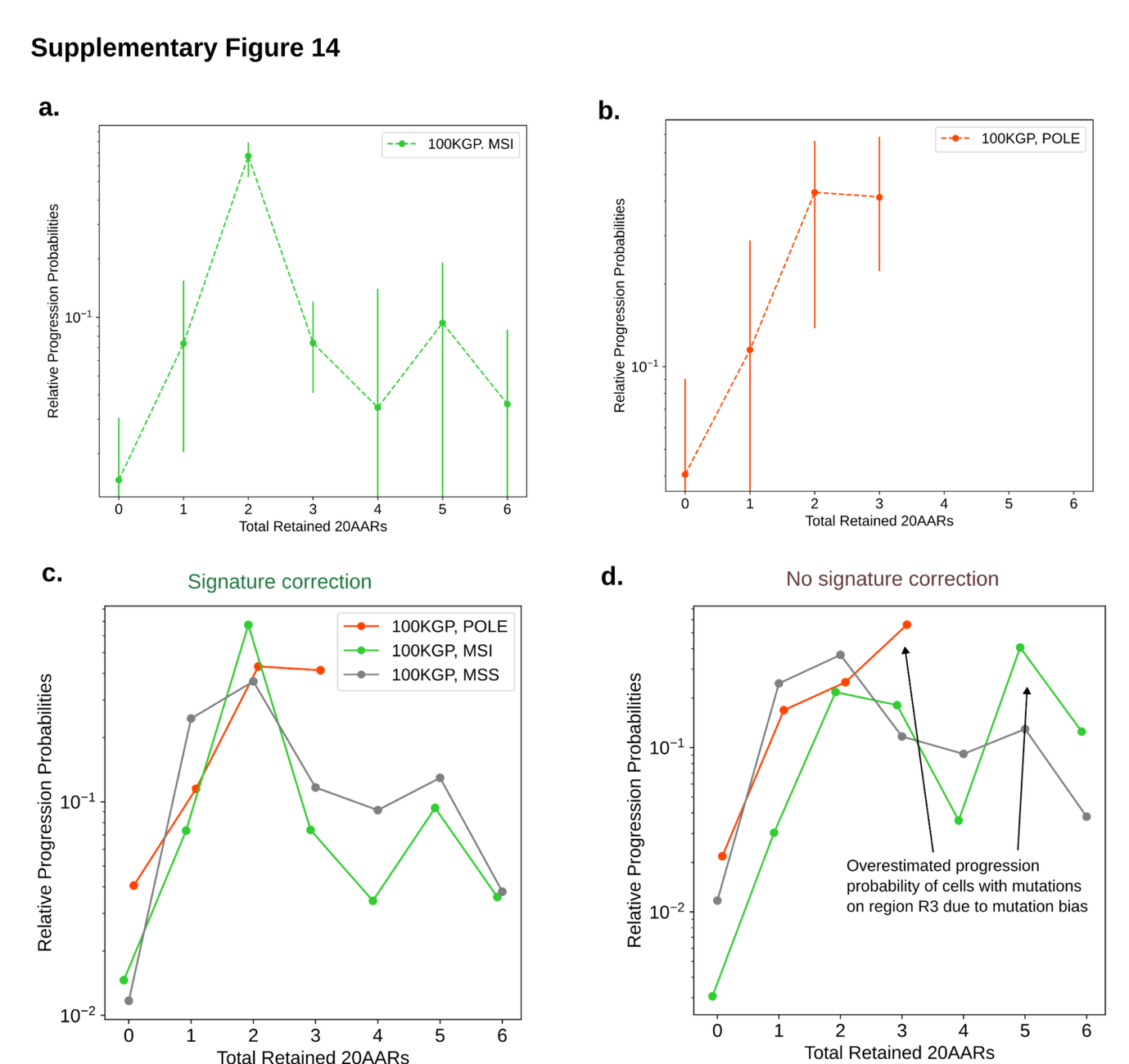
Supplementary Figure 14.** Signature correction in hypermutant CRCs.

The relative progression probabilities by total number of 20AARs retained in MSI (a) and POLE-mutant CRCs (b) in the 100kGP cohort, with bootstrapped 95% confidence intervals. (c) The relative progression probabilities by total number of 20AARs retained in POLE-mutant, MSI and MSS CRCs 100kGP cohort, display overall agreement, which can be quantified by no significant differences in the progression-weighted mean number of 20AARs (Δ_MSS-POLE_=-0.22, 95% CI=[-0.62, 0.15], Δ_MSS-MSI_=-0.29, 95% CI=[-0.67, 0.08], bootstrapping). (d) As in (c) but without correcting for hypermutant mutational signatures. We calculate larger differences in the progression-weighted mean number of 20AARs, that are statistically significant in the case of MSI tumours (Δ_MSS-POLE,nc_=-0.31, 95% CI=[-0.78, 0.2], Δ_MSS-MSI,nc_=-1.87, 95% CI=[-2.51, -0.81]). We highlight that the relative progression probability is larger for 5-6 total retained 20AARs for the MSI cohort without correction, due to an indel associated mutation bias to region R3.
