# Supplementary material for: Mathematical Modeling Quantifies “Just-Right” APC Inactivation for Colorectal Cancer Initiation
Source: Cancer Res. Author manuscript; Available in PMC 2025 Nov 25. (PMC7618390; doi:10.1158/0008-5472.CAN-25-0445)
Supplement: Supplementary file 1 [file EMS209718-supplement-Supplementary_Notes.pdf]

Supplementary Notes

1. An extended mathematical model of cancer progression of double mutant *APC* cells

We outline an extended model of APC-driven CRC initiation in which mutation and progression to cancer depend on the type X of the *APC* double mutant. We will proceed with minimal assumptions, allowing mutation arrival and progression to cancer to be time (or age) dependent, and eventually reconcile this with our simplification in the manuscript: that the relative differences in selection $\tilde{p}_X$ can be inferred from the fraction of frequency in CRCs f_X over the relative mutation probability m_X, $\tilde{p}_X \propto f_X/m_X$, where all terms are time-independent.

Cells with double allelic *APC* mutations appear in the colon as a Poisson process at rate $\lambda(t)$, that depends on age t. In Supplementary Note 3 we outline an approximation for $\lambda(t)$, however the same results would hold for other functional forms. Under the assumption that the first hit's effect is independent of its mutation type, the relative probability m_X of type X is a constant proportional to the product of the mutation probabilities of the two hits (Equation M1 in the Methods). Thus, double mutants of type X arrive at rate $m_X\,\lambda(t)$, where the relative mutation probability m_X is time-independent. Which ones will progress to CRC? We may assume that there is a time-independent component to the progression probability, $\tilde{p}_X$, as well as a component that depends on the time t at which the double mutant arrived and the age of the patient, T. We denote this by $q_X(t, T-t)$. Then, due to the splitting property of Poisson processes, the expected number of type X double mutants that result in a CRC diagnosed by age T is

$$\int_0^T m_X \lambda(t)\, \tilde{p}_X\, q_X(t,\, T-t)dt.$$

We estimate the frequency of type X CRCs by age T (amongst all other double mutant *APC* CRCs diagnosed by age T) as

$$f_X(T) = \frac{m_X \tilde{p}_X \int_0^T \lambda(t)\, q_X(t, T-t)dt}{\sum_y m_y \tilde{p}_y \int_0^T \lambda(t)\, q_y(t, T-t)dt}.$$

The observation in the data is that the frequency of genotypes does not depend on age (Supplementary Figure 8). For that to be the case, we need the right hand side to be independent of T. Thus, we define the relative progression probabilities $\tilde{p}_X$ such that

$$f_X(T) = \frac{m_X \tilde{p}_X \int_0^T \lambda(t)\, q_X(t, T-t)dt}{\sum_y m_y \tilde{p}_y \int_0^T \lambda(t)\, q_y(t, T-t)dt} \approx \frac{m_X \tilde{p}_X}{\sum_y m_y \tilde{p}_Y},$$

which is embedded in our assumption that any additional factors affecting progression to CRC act uniformly across *APC* genotypes. In the absence of further data, this approximation seems justified by the empirical time-independence of $f_X(T)$. Nonetheless, we next outline more detailed scenarios under which the time-independence assumption would hold.

For example, we may assume that the time-dependent component of the progression probability does not depend on the type of the mutation, that is, $q_X(t,\ T-t) = q(t,\ T-t)$ for all types X. Then,

$$f_X(T) = \frac{m_X \tilde{p}_X}{\sum_y m_y \tilde{p}_y} \frac{\int_0^T \lambda(t)\, q(t, T-t) dt}{\int_0^T \lambda(t)\, q(t, T-t) dt} = \frac{m_X \tilde{p}_X}{\sum_y m_y \tilde{p}_y}.$$

Thus, our definition of time-independent relative progression probability is exact, and we recover that $f_X \propto m_X \tilde{p}_X$ is time-independent as observed in the data. Note that the *absolute* progression probability of a genotype is age-dependent, namely

$$p_{M,N}(T) = \frac{f_{M,N}}{m_{M,N} \int_0^T \lambda(t)\, q(t, T-t) dt},$$

however the age-dependency cancels when we calculate the relative progression probability,

$$\tilde{p}_{M,N} = \frac{p_{M,N}(T)}{\sum_{i,j} p_{i,j}(T)} = \frac{f_{M,N}/m_{M,N}}{\sum_{i,j} f_{i,j}/m_{i,j}},$$

which is precisely Equation M2 in Methods with $C = C(T) = \frac{1}{\int_0^T \lambda(t)\, q(t, T-t) dt}$.

This scenario is biologically plausible: for example, if the progression probabilities denote the survival probability of the double mutant cell, with 'too high' or 'too low' Wnt activation resulting in cell death or senescence. In that case, the frequencies of CRCs of different types are independent of age, thus there is no 'first versus fittest' concern, and age does not confound the measurements of selection.

Instead of assuming time independent progression probabilities we could suppose that double mutants of type X that eventually progress to cancer take $\tau + \tau_X$ time to become detectable CRCs, where τ is the common time from initiation to detection of any CRC and τ_X is the differential time it takes for a type X cell to grow into a detectable CRC. That is, $q_X(t,\ s) = p_X$ if $s < \tau + \tau_x$ and 0 otherwise, and hence in the leading order

$$f_X(T) = \frac{m_X \tilde{p}_X \int_0^{T-\tau-\tau_X} \lambda(t)\, dt}{\sum_y m_y \tilde{p}_y \int_0^{T-\tau-\tau_y} \lambda(t)\, dt} \approx \frac{m_X \tilde{p}_X (T-\tau-\tau_x)^2}{\sum_y m_y \tilde{p}_y (T-\tau-\tau_y)^2} \approx \frac{m_X \tilde{p}_X}{\sum_y m_y \tilde{p}_y}$$

where we used our approximation $\lambda(t) = 0.1 \cdot t$ for the initiation rate (Supplementary Note 3) and that the progression time only weakly depends on type, $T - \tau >> \tau_X$. In particular, this suggests that measurements of selection in elder patients (large T) should be minimally confounded by age. From the data, if we consider the frequencies of 20AARs in patients >81 years old, we recover roughly the same curve of relative progression probabilities p_X as averaged across ages (Supplementary Figure 8d). In summary, whilst cancer progression is an age and time-dependent process, the CRC data supports approximating the relative progression probabilities of double mutant *APC* cells with different genotypes as time-independent.

2. A model of mutation accumulation in healthy crypts

Here we outline the derivation of Equation M9 to estimate the expected number of double mutant *APC* cells as a function of age. We consider a Poisson process model of mutation accumulation. The model assumes that the population of crypts is constant in time, and that single mutant crypts do not gain a growth advantage. Whilst these are common assumptions in the literature [7], we emphasize that they are not required to derive the progression probabilities outlined in Methods 2.2, which only rely on the assumption that the first *APC* mutation does not have differential phenotypic effects depending on the position or type of the mutation.

We model the accumulation of *APC* mutations in the healthy colon. The human colon consists of $N \approx 5 \cdot 10^7$ (range $10^7 - 10^8$) crypts, and each crypt harbours an average of $n_s \approx 6$ range (5-7) active stem cells [8–10]. Cells in the healthy colon acquire mutations at a rate of $\mu_{sbs} \approx 1.45 \cdot 10^{-8}$ substitutions per base-pair per year [5]. By combining mutational signatures with the *APC* genomic context, we estimate the rate of *APC* stop-gain and frameshift mutations at regions R_i, with a total rate in any region of $\mu_{APC} = 429 \cdot \mu_{sbs} \approx 6.22 \cdot 10^{-6}$ per cell per year (Methods 2.2). The first *APC* hit can also result from loss of an *APC* allele, which we estimate occurs at rate $\mu_{-} \approx 4.72 \cdot 10^{-6}$ per year (Methods 2.2, and Supplementary Note 1). We model the arrival of single mutant cells as a Poisson process with constant rate $N n_s (\mu_{APC} + \mu_{-})$ per year. Once a stem cell acquires a first *APC* mutation, its fixation probability in the crypt can be estimated by a Moran model result,

$$p_f = \frac{1-1/f}{1-1/f^{n_s}} \approx 0.02 ,$$

where f denotes the fitness of the stem cell with the mutation, estimated to be 1.63 [9]. We assume that the fixation probability is the same for all mutation types and that fixation is instantaneous. Thus, in total single mutant *APC* crypts arrive at rate $N n_s (\mu_{APC} + \mu_{-}) p_f$ per year, and the expected numbers of single mutant crypts by time t with a single *APC* mutation and a copy-loss are

$$c_{APC}(t) = N n_s \mu_{APC} p_f t \text{ and } c_{-}(t) = N n_s \mu_{-} p_f t, \quad \text{Eq S1}$$

respectively. We assume that stem cells in single mutant crypts effectively behave as wild type cells, and continue to accumulate *APC* mutations. If the first hit is a copy-loss event, the second hit must be a truncating mutation. If the first hit is a truncating mutation, the second hit can result from a second truncating mutation, a copy-loss event or a copy-neutral LOH event, which we estimate occur at rate $\mu_{x2} = 7.18 \cdot 10^{-5}$ per year (Methods 2.2 and Supplementary Note 1). Thus, the second hit arrives as a constant rate Poisson process within the population of stem cells in single mutant crypts. If we approximate the number of single mutant crypts at time t by its expected value, the total arrival rate of double mutant *APC* cells at time t is

$$\lambda(t) = n_s (\mu_{APC} + \mu_{-} + \mu_{x2}) c_{APC}(t) + n_s c_{-}(t) \quad \text{Eq S2}$$

Substituting the aforementioned parameters, we calculate that $\lambda(t) \approx 0.1\,t$. The expected number of double mutants to have arrived by time t is

$$\Lambda(t) = \int_0^t \lambda(s)ds \quad \text{Eq S3.}$$

In particular, we can calculate that $\Lambda(80) \approx 580$ double mutants of any type will arrive within the 80 years lifespan of an individual (Supplementary Figure 9). To calculate the rate of arrival of double mutants with genotype (M, N), we consider the rates of the relevant mutational path or, equivalently, we thin the Poisson arrivals by the probability that, given that a double mutant arrives, it has genotype (M, N) - given by our estimates $m_{(M,N)}$. Finally, the rate of arrival of double mutants with X total retained 20AARs is the sum of the rates of double mutants with genotypes that retain X 20AAARs (by superposition of Poisson processes). For example, the rate of arrival of double mutants with 0 20AARs by time t is

$$(m_{(0,0)} + m_{(0,-)} + m_{(0,x2)})\,\Lambda(t)$$

As plotted in Supplementary Figure 9, all types of mutants are expected to arrive within a lifespan. This does not consider the accumulation of third hits, which is justified by the paucity of triple mutants in the data as well as the small mutation rate relative to the rate of arrival of double mutants.

3. The 15AARs

The first 15AAR has been demonstrated to be involved in β-catenin degradation mediated by truncated APC [1]. However, we observed that the slope of the cumulative mutation counts in CRCs does not increase around the 15AAR positions (Supplementary Figure 2), suggesting that there is no strong selection to truncate or retain it. To further assess this, we calculated the fraction of mutations retaining the 15AAR expected out of mutations that retain 0 20AARs using the context-specific mutational processes. This fraction is estimated to be 0.188, whilst the observed fraction of tumors retaining the 15AAR, out of all those that retain 0 20AARs, is 0.239. Using these estimates, we calculated that the progression probability of retaining the 15AARs relative to probability of retaining other 0 20AAR mutations is 1.24 (95% bootstrap CI 0.95-1.55). Thus, the data is consistent with no significant advantage when retaining 15AAR relative to other 0 20AAR mutations.

4. Estimating region mutation probabilities from signature data and genomic context

This section provides further detail of our method to estimate the probability m_j that a truncating mutation falls in region R_j of *APC*, for $j \in \{0, 1, 2, 3\}$, described in Methods 2.2.1.

First, we estimate the probability of the truncating mutation being a stop-gain versus a frameshift as follows. The ratio of SBS to indels in healthy crypts was found to be 24:1 [5]. Considering the number of single-base substitutions that can result in a stop codon upstream of the SAMP repeat, we find that (737)/(3*4717) ≈ 5.2% of SBS result in a stop-gain mutation. The proportion of insertions and deletions in healthy colon crypts [5] that disrupt the reading frame is $\approx 88\%$. Thus, we estimate that the ratio of stop-gain to frameshifts in *APC* in normal tissue is approximately (24*5):(1*88) = 15:11, which gives that truncating mutations are stop-gain with probability $p_{stop\text{-}gain}$=15/26≈0.58 and frameshifts with probability p_{fshift}=11/26≈0.42. Notably, the frequency of stop-gain mutations amongst truncating *APC* mutation is similar in the 100kGP MSS cohort, 1194/1906≈0.63.

Next, we calculate the probability that a stop gained or a frameshift falls in region R_j . Following the COSMIC categorisation, we classify SBS mutations into the conventional six substitution subtypes as well as the nucleotides immediately 5' and 3' to the mutation, resulting in 96 SBS types (represented with central pyrimidine base). For indels, we adopted the COSMIC categorisation but omitted micro-homology ID classes (which account for <5% of IDs observed in healthy colonic crypts [5]), resulting in 71 ID types. We can then calculate the probability that a mutation of type x occurs in locus i as the following product

P(new mutation is of type x and occurs at locus i)=P(new mutation occurs at locus i given that it is of type x)·P(new mutation is of type x)

To calculate the first term of the product, for each mutation type x we count the number n_x of loci compliant with the mutation, defined as the loci in which the mutation could occur - considering also the complementary strand due to the convention of defining mutation types on pyrimidine centred substitutions. For example, for the sequence 'GCGT', the second position is compliant with G[C>A]G, G[C>G]G and G[C>T]G, whilst by considering the complementary strand, the third position is compliant with A[C>A]G, A[C>G]G and A[C>T]G. Then, for a given mutation type x, the probability that the mutation occurs in locus i is 0 if that locus is not compliant with the mutation, or $1/n_x$ if it is, which gives P(new mutation occurs at loci i given that it is type x)· This is because the chance that the new SBS falls at a given locus is the reciprocal of the number of loci in which the mutation of type x could occur.

We next estimated P(new mutation is of type x) separately for SBSs and indels under the following rationale: Mutations are caused by the mutational processes active in the normal colon, and given that process y causes a new mutation, the probability it is of type x can be estimated using mutational signatures. Thus,

$$\text{P(new mutation is of type x)}=\sum_{y}\text{P(mutation type x given process y)} \cdot \text{P(mutation caused by process y)}.$$

To estimate P(mutation caused by process y) we used signature data reported from sequencing 31 non-dysplastic healthy colonic crypts [5]. We considered ubiquitous signatures that were present in over 85% of samples, which for SBSs were SBS1, SBS5, SBS18 and for IDs, were ID1, ID2 and ID5. For SBS and IDs separately, the reported exposure vectors for each crypt were normalised such that the contributions from the ubiquitous signatures summed to 1, and we then averaged over crypt samples to create average normalised exposure vectors for SBS and IDs - which are estimates for P(mutation caused by process y). P(mutation type x given process y) is the weighting of type x for the mutational signature associated with y.

To calculate the probability that a new stop-gain occurs in region R_j, we summed P(new mutation is of type x and occurs at locus i in *APC*) for all loci i and all SBS mutation types that result in a stop-codon in R_j, and normalised such that

$$\sum_j P(\text{new stop-gain in } R_j) = 1.$$

An analogous procedure gives the probabilities for frameshift mutations by adding the indel types that result in a change of the reading frame. This gives P(new stop-gain in R_j) and P(new frameshift in R_j) for all regions.

Finally, the region probabilities were weighted by the relative probabilities of stop-gain or frameshift mutations ($p_{stop\text{-}gain}$ and p_{fshift} calculated above) normalized to get the probability m_j that, given that a new truncating mutation occurs, this falls in region R_j

$$m_j = (p_{stop\text{-}gain} \cdot P(\text{new stop-gain in } R_j) + p_{fshift} \cdot P(\text{new frameshift in } R_j)) \,/\, K$$

where the normalizing constant is

$$K = \sum_{k=0}^{3} (p_{stop\text{-}gain} \cdot P(\text{new stop-gain in } R_k) + p_{fshift} P(\text{new frameshift in } R_k)).$$

Taking the ratios of the regions R_j and R_0 we get m_j/m_0 for $j \in \{0, 1, 2, 3\}$ as desired.

5. The rate of loss of heterozygosity (LOH)

We estimated the rates of copy-loss and copy-neutral LOH at the *APC* locus of chromosome 5 (5q22.1–q22.3) in the healthy colon by assuming phenotypic equivalence between tumors with varied molecular causes of APC loss (Methods), resulting in estimates of $5.72*10^{-6}$ and $7.18*10^{-6}$/cell/year, respectively.

Paterson *et al*. [7] estimated the rate of LOH through a different strategy, obtaining a higher rate of $1.36*10^{-4}$/cell/year. Paterson *et al* used the ratio of MSI cancers with two inactivating mutations in *APC* to those with one inactivating mutation and one LOH event to be 1:7, as reported by Huang *et al*. [11] based on protein assays of n=55 CRCs. This ratio is in high discordance with the ratio in the 100kGP cohort of 10:1, based on a large number of whole-genome sequenced samples (n=2,023)[2]. Using the inference method of Paterson *et al.* with data from 100kGP we obtained a rate of LOH of $3.89*10^{-6}$/cell/year, which is comparable to our estimate.

6. The third 20AAR

Our finding that most variability in the progression probabilities of APC biallelic genotypes is explained by the total 20AARs retained (Figure 3d), independent of the mutational process, is seemingly in discrepancy with previous work that studied the binding affinity of β-catenin with the different 20AARs and found that the 3rd 20AAR binds strongest[12,13]. Here we discuss this further.

The aforementioned studies were *in vitro* and assessed the binding between β-catenin and a single species of truncated APC. We argue that this might be hard to extrapolate to the biological context within tumors, where a combination of truncated APC proteins with different lengths are being expressed (from the two alleles), potentially diluting the effect. In our data, we find that double mutants that retain the 3rd 20AAR have similar progression probabilities to double mutants that have the same total number but do not retain the 3rd 20AAR (no statistically significant differences between the progression probabilities of (2,1), (3,0) and (3,-), and of (2,2) and (3,1), Figure 3d). However, the point estimates are lower for the genotypes that retain the 3rd 20AAR, consistent with its binding stronger. Moreover, (3,-) has a considerably lower progression probability than either (2,1) or (3,0) which is consistent with our explanation that biallelic expression dilutes the signal of the strong 3rd 20AAR binding. Furthermore, other experimental work has shown that the APC domains cooperatively enhance β-catenin recruitment and phosphorylation[14,15], suggesting that their role in Wnt regulation might extend beyond directly binding to β-catenin.

Thus, whilst the 3rd 20AAR might bind more strongly, resulting in differences across genotypes that retain the same number of 20AARs, this effect is not large enough to result in a statistically significant signal in the cancer data. Across all genotypes, independent cancer datasets support that the relative progression probability of double mutant *APC* colonic cells is mainly dependent on the total number of retained 20AARs.

The data and scripts used are available at https://github.com/xellbrunet/APC_Analysis_Public.

Supplementary References

1. Kohler EM, Brauburger K, Behrens J, Schneikert J. Contribution of the 15 amino acid repeats of truncated APC to beta-catenin degradation and selection of APC mutations in colorectal tumors from FAP patients. Oncogene. 2010;29: 1663–1671.

2. Cornish AJ, Gruber AJ, Kinnersley B, Chubb D, Frangou A, Caravagna G, et al. The genomic landscape of 2,023 colorectal cancers. Nature. 2024;633: 127–136.

3. Christie M, Jorissen RN, Mouradov D, Sakthianandeswaren A, Li S, Day F, et al. Different APC genotypes in proximal and distal sporadic colorectal cancers suggest distinct WNT/β-catenin signalling thresholds for tumourigenesis. Oncogene. 2013;32: 4675–4682.

4. Lindeboom RGH, Supek F, Lehner B. The rules and impact of nonsense-mediated mRNA decay in human cancers. Nat Genet. 2016;48: 1112–1118.

5. Lee-Six H, Olafsson S, Ellis P, Osborne RJ. The landscape of somatic mutation in normal colorectal epithelial cells. Nature. 2019. Available: https://www.nature.com/articles/s41586-019-1672-7

6. Guinney J, Dienstmann R, Wang X, de Reyniès A, Schlicker A, Soneson C, et al. The consensus molecular subtypes of colorectal cancer. Nature medicine. 2015;21. doi:10.1038/nm.3967

7. Paterson C, Clevers H, Bozic I. Mathematical model of colorectal cancer initiation. Proc Natl Acad Sci U S A. 2020;117: 20681–20688.

8. Tomasetti C, Vogelstein B. Cancer etiology. Variation in cancer risk among tissues can be explained by the number of stem cell divisions. Science. 2015;347: 78–81.

9. Vermeulen L, Morrissey E, van der Heijden M, Nicholson AM, Sottoriva A, Buczacki S, et al. Defining stem cell dynamics in models of intestinal tumor initiation. Science. 2013;342: 995–998.

10. Baker A-M, Cereser B, Melton S, Fletcher AG, Rodriguez-Justo M, Tadrous PJ, et al. Quantification of crypt and stem cell evolution in the normal and neoplastic human colon. Cell Rep. 2019;27: 2524.

11. Huang J, Papadopoulos N, McKinley AJ, Farrington SM, Curtis LJ, Wyllie AH, et al. APC mutations in colorectal tumors with mismatch repair deficiency. Proc Natl Acad Sci U S A. 1996;93: 9049–9054.

12. Kohler EM, Derungs A, Daum G, Behrens J, Schneikert J. Functional definition of the mutation cluster region of adenomatous polyposis coli in colorectal tumours. Hum Mol Genet. 2008;17: 1978–1987.

13. Liu J, Xing Y, Hinds TR, Zheng J, Xu W. The third 20 amino acid repeat is the tightest binding site of APC for beta-catenin. J Mol Biol. 2006;360: 133–144.

14. Ranes M, Zaleska M, Sakalas S, Knight R, Guettler S. Reconstitution of the destruction complex defines roles of AXIN polymers and APC in β-catenin capture, phosphorylation, and ubiquitylation. Mol Cell. 2021;81: 3246–3261.e11.

15. Novellasdemunt L, Foglizzo V, Cuadrado L, Antas P, Kucharska A, Encheva V, et al. USP7 is a

tumor-specific WNT activator for APC-mutated colorectal cancer by mediating β-catenin deubiquitination. Cell Rep. 2017;21: 612–627.

16. Gao J, Aksoy BA, Dogrusoz U, Dresdner G, Gross B, Sumer SO, et al. Integrative analysis of complex cancer genomics and clinical profiles using the cBioPortal. Sci Signal. 2013;6: l1.

17. de Bruijn I, Kundra R, Mastrogiacomo B, Tran TN, Sikina L, Mazor T, et al. Analysis and Visualization of Longitudinal Genomic and Clinical Data from the AACR Project GENIE Biopharma Collaborative in cBioPortal. Cancer Res. 2023. doi:10.1158/0008-5472.CAN-23-0816

18. Robinson PS, Coorens THH, Palles C, Mitchell E, Abascal F, Olafsson S, et al. Increased somatic mutation burdens in normal human cells due to defective DNA polymerases. Nat Genet. 2021;53: 1434–1442.
